# Brain aging patterns among nine neurological disorders: A case-control study

**DOI:** 10.1371/journal.pmed.1004860

**Published:** 2026-07-21

**Authors:** Chuang Liang, Godfrey Pearlson, Juan Bustillo, Peter Kochunov, Jiayu Chen, Xiangrong Zhang, Rongtao Jiang, Kent E. Hutchison, Jing Sui, Zening Fu, Xiao Yang, Yuhui Du, Daoqiang Zhang, Shile Qi, Vince D. Calhoun

**Affiliations:** 1 Department of Artificial Intelligence, Nanjing University of Aeronautics and Astronautics, Nanjing, Jiangsu, China; 2 Key Laboratory of Brain-Machine Intelligence Technology, Ministry of Education, Nanjing University of Aeronautics and Astronautics, Nanjing, Jiangsu, China; 3 Olin Neuropsychiatry Research Center, Institute of Living, Hartford, Connecticut, United States of America; 4 Departments of Neurosciences and Psychiatry and Behavioral Sciences, University of New Mexico, Albuquerque, New Mexico, United States of America; 5 Department of Psychiatry, University of Maryland School of Medicine, Baltimore, Maryland, United States of America; 6 Tri-institutional Center for Translational Research in Neuroimaging and Data Science (TReNDS) Georgia State University, Georgia Institute of Technology, Emory University, Atlanta, Georgia, United States of America; 7 Department of Psychiatry, The Affiliated Brain Hospital of Nanjing Medical University, Nanjing, Jiangsu, China; 8 State Key Laboratory of Cognitive Neuroscience and Learning, Beijing Normal University, Beijing, China; 9 Department of Psychology and Neuroscience, University of Colorado Boulder, Boulder, Colorado, United States of America; 10 Huaxi Brain Research Center, West China Hospital of Sichuan University, Chengdu, China; 11 School of Computer and Information Technology, Shanxi University, Taiyuan, Shanxi, China; University of Cambridge, UNITED KINGDOM OF GREAT BRITAIN AND NORTHERN IRELAND

## Abstract

**Background:**

The difference between neuroimaging-predicted brain age and chronological age, the predicted age difference (PAD), has been studied as a potential biomarker reflecting individual brain health. Although previous large-scale studies have shown that brain age deviations occur across multiple disorders, cross-disorder comparisons of PAD within a unified framework, together with identification of the neuroimaging features associated with these differences and their related gene expression profiles, remain limited. Our aims are to systematically compare brain aging across multiple common brain disorders and explore the brain patterns and biological processes underlying these differences.

**Methods and findings:**

In this study, structural MRI data from 45,900 healthy controls (HCs) and 2,698 patients with developmental disorders (attention-deficit/hyperactivity disorder [ADHD] and autism spectrum disorder [ASD]), addiction (alcohol use disorder [AUD], tobacco use disorder [TUD], and AUD&TUD-A&TUD), dementia (Alzheimer’s disease [AD], and mild cognitive impairment [MCI]) or other psychiatric disorders (schizophrenia [SZ], bipolar disorder [BP], and major depressive disorder [MDD]), were collected to generate PAD, along with transcriptome data. Then, we calculated the PAD difference between patient and HC as Cohen’s *d* effect sizes, derived from a linear model that accounted for age, age^2^, sex, and site, and further identified the interpretable brain patterns associated with the PAD difference for each diagnostic group. Finally, enrichment analyses was conducted to identify the biological function of genes relatively over- or underexpressed in association with these patterns. Results showed that while PAD was consistently greater across disorders, different brain disorders showed different degrees of abnormality, the highest effects in dementia (AD: *d* = 0.97, 95% confidence interval (CI) [0.82,1.13]; *p* < 0.001 and MCI: *d* = 0.45, 95% CI [0.34,0.56]; *p* < 0.001), followed by addiction (A&TUD: *d* = 0.84, 95% CI [0.44,1.23]; *p* < 0.001, TUD: *d* = 0.72, 95% CI [0.49,0.96]; *p* < 0.001, and AUD *d* = 0.62, 95% CI [0.39,0.84]; *p* < 0.001) and *p*sychiatric disorders (SZ: *d* = 0.53, 95% CI [0.30,0.76]; *p* < 0.001, BP: *d* = 0.46, 95% CI [0.22,0.69]; *p* < 0.001 and MDD: *d* = 0.28, 95% CI [0.11,0.46]; *p* < 0.001), but not different from ex*p*ected in develo*p*mental disorders (ASD: *d* = 0.06, 95% CI [−0.04,0.16]; *p* = 0.36) and ADHD: *d* = 0.01, 95% CI [−0.14,0.15]; *p* = 0.98). Furthermore, higher PAD values in patient grou*p*s were linked to s*p*ecific spatial brain patterns, including the frontotemporal network in psychiatric disorders, default mode network-salience network-putamen-thalamus in addiction and fronto-occipital network in dementia. Prefrontal cortex involvement was common across disorders, and disorder-specific brain patterns associated genes were enriched in different biological processes. A limitation of our study is that psychiatric disorders and addiction have high comorbidity, and these potential confounders were not considered.

**Conclusions:**

In summary, the different brain aging patterns, each based around specific underlying circuits, may serve as neuroimaging biomarkers for understanding the neural aging mechanisms in commonly occurring brain disorders. Future studies should test whether these disorder-specific brain aging patterns can serve as useful biomarkers to guide critical clinical decision-making.

## Author summary

**Why was this study done?:**

- The difference between brain age estimated from MRI scans and actual age (PAD) can reflect brain health.
- Previous studies have shown that many common brain disorders are linked to accentuated brain aging (increased PAD compared to healthy control (HC)).
- However, it remains unclear whether brain aging follows similar or distinct patterns across different disorders and how these differences can be interpreted biologically.
- We aimed to systematically compare brain aging across multiple common brain disorders and explore the brain patterns and biological processes underlying these differences.

**What did the researchers do and find?:**

- We analyzed brain imaging data from 45,900 healthy individuals and 2,698 patients with developmental disorders, addiction, dementia, or other psychiatric disorders, to compare the PAD difference between patient and HC and identify the brain regions and biological processes underlying these differences.
- We found that brain age was generally higher in patients, with the largest increases observed in dementia, followed by addiction and psychiatric disorders, but not in developmental disorders.
- Different disorders showed distinct brain regions associated with these differences, with a shared involvement of the prefrontal cortex, and these related genes were linked to different biological functions.

**What do these findings mean?:**

- Accentuated brain aging is a common result for many disorders, with the prefrontal cortex may underlie the important neural processes with respect to the universality of accentuated brain aging.
- The biological processes of genes related to identified brain patterns can help us to better understand brain alterations related to aging at a molecular level.
- In the future, the brain patterns we identified for each disorder could be tested for their usefulness as biomarkers to guide critical clinical decision-making.
- The main limitation is that we did not account for comorbidities between psychiatric disorders and addiction, which may have introduced potential confounding effects and influenced the observed results.

## Abstract 3

In this case-control study, Chuang Liang and colleagues systematically compare brain aging patterns and biological processes across multiple common brain disorders.

## Introduction

The difference between age predicted from structural or functional neuroimaging data and chronological age, predicted age difference (PAD), can be used as an index to quantify individuals’ deviation from a normative brain aging [1,2]. A positive PAD value indicates that an individual’s brain age is greater than their chronological age, termed accentuated aging, while a negative PAD implies a delay from expected aging. Brain age prediction based on machine learning techniques has been widely used in investigating brain disorders to assess whether these disorders cause deviations in brain aging pattern [3–5]. These observations can provide a starting point for understanding the underlying neuropathological mechanisms of brain disorders.

Previous studies have demonstrated the phenomena of accentuated brain aging or delayed development in a variety of commonly occurring brain disorders. Brain age prediction based on gray matter in Alzheimer’s disease (AD) revealed that the pathological structural atrophy in AD is associated with accentuated aging, with PAD of +10 years [6]. Mild cognitive impairment (MCI), a possible precursor to AD, is also associated with increased PAD, which has been shown increase accuracy of predicting conversion of MCI to AD and may serve as a biomarker for early AD risk screening [7]. The large, worldwide Enhancing Neuro-Imaging Genetics through Meta-analysis (ENIGMA)-schizophrenia (SZ) also reported a significantly higher PAD in SZ, compared to healthy controls (HCs) [8]. Another large dataset-based structural brain age prediction study reported a moderate increase in PAD in bipolar disorder (BP) compared to HC [9]. In major depressive disorder (MDD), accentuated brain aging seems to be stage-dependent, occurring at illness onset and disappearing as the illness further advances [10]. Moreover, higher brain PAD is associated with health-related lifestyle factors, and consistently reported in individuals with alcohol use disorder (AUD) and tobacco use disorder (TUD) [11]. Attention-deficit/hyperactivity disorder (ADHD) and autism spectrum disorder (ASD) typically emerge in childhood with disorder-related brain changes being highly age-dependent, meaning that patterns of accentuated aging, typical development, or delayed development may switch between different age groups [12–14].

Although a number of brain disorders have been investigated using PAD metrics, several issues remain to be addressed. First, while some studies have included relatively large multi-disorder samples, sample size constraints remain a persistent challenge in brain age prediction research. This limits generalizability and repeatability and may be unable to uncover the full spectrum of brain aging variations across different brain disorders. Second, predicted brain age is typically studied as a single whole-brain measure, thereby neglecting specific spatial brain patterns underlying the brain age prediction, leading to limited biological interpretability of the PAD. Third, although genetic differences have been demonstrated to explain a portion of the inter-individual variability in PAD [15], the genetic mechanisms underlying specific brain patterns associated with PAD difference in common brain diseases remain unclear. To address the above limitations, we combined transcriptome and structural neuroimaging data to uncover the deviation from normative brain aging, examine spatial brain patterns associated with the PAD difference and related gene expression profiles among common brain disorders in large samples. This is an important step towards utilizing PAD as potential biomarkers to assist clinicians in disentangling shared and specific pathophysiological processes of common brain disorders.

**Fig 1:**
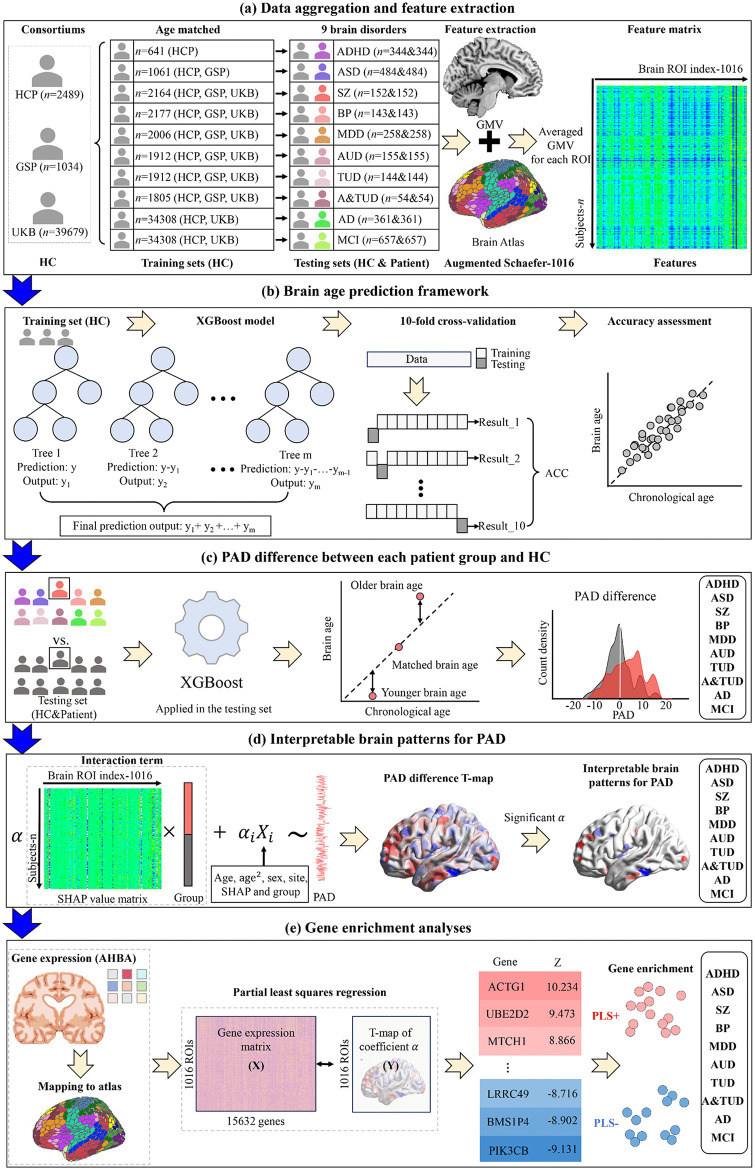
Flowchart of the study design. **(a)** The averaged gray matter volume (GMV) from each region of interest (ROI, augmented Schaefer-1016 brain atlas) were extracted from testing (ADHD, ASD, SZ, BP, MDD, AUD, TUD, A&TUD, AD, and MCI) and age-range matched training (healthy participants from HCP, GSP, and UKB) sets. **(b)** The performance of the extreme gradient boosting (XGBoost) model was verified on training set by 10-fold cross-validation. **(c)** The XGBoost model was applied to the testing set to generate individual brain age predictions and the PAD difference between specific patient group and HC was compared. **(d)** Identifying the brain patterns associated with the PAD difference in different diagnostic groups by incorporating interaction terms (Shapley Additive Explanations-SHAP × group) in multiple linear regression models. The age-corrected PAD was the dependent variable, while age, age^2^, sex, site, group, SHAP value, and SHAP × group were the independent variables. **(e)** Enrichment analyses on the genes that relatively over- or underexpressed in association with PAD difference T-map. ADHD, attention-deficit/hyperactivity disorder; ASD, autism spectrum disorder; SZ, schizophrenia; BP, bipolar disorder; MDD, major depressive disorder; AUD, alcohol use disorder; TUD, tobacco use disorder; A&TUD, AUD and TUD; AD, Alzheimer’s disease; MCI, mild cognitive impairment; HC, healthy control; HCP, Human Connectome Project; GSP, Brain Genomics Superstruct Project; UKB, UK Biobank; PAD, predicted age difference.

**Fig 2:**
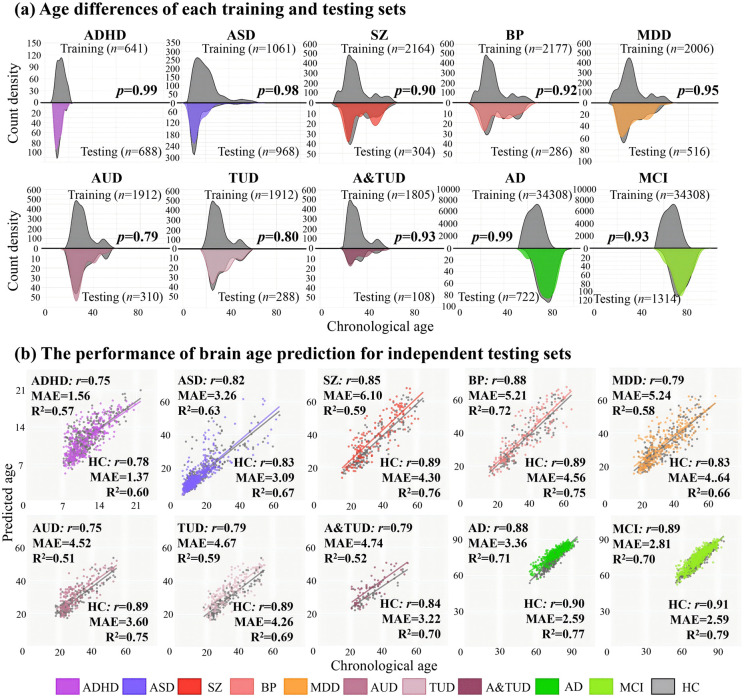
Age distributions and the performance of brain age predictions. **(a)** Age distributions of each training (up) and testing (down) sets. The gray shading under each diagnostic group represents its age-, sex-, and number-matched control group. *P* value represents the group difference of chronological age between the patient and HC calculated by two-sample *t* test. **(b)** Pearson correlations between the predicted brain age and chronological age, along with MAE and *R*^2^, in independent testing sets. The solid line indicates the linear regression fit. ADHD, attention-deficit/hyperactivity disorder; ASD, autism spectrum disorder; SZ, schizophrenia; BP, bipolar disorder; MDD, major depressive disorder; AUD, alcohol use disorder; TUD, tobacco use disorder; A&TUD, AUD and TUD; AD, Alzheimer’s disease; MCI, mild cognitive impairment; HC, healthy control; MAE, mean absolute error; *R*^2^, coefficient of determination.

In this study, structural magnetic resonance imaging (sMRI) data including HCs (*n* = 45,900) and common brain disorders (344 ADHDs, 484 ASDs, 152 SZs, 143 BPs, 258 MDDs, 155AUDs, 144 TUDs, 361 ADs, and 657 MCIs) were used to generate the individual brain age predictions in each diagnostic group (including patients and the matched HCs, Fig 1a and 1b). Our aims including: (1) comparing PAD difference across different diagnostic groups, and among age and sex subgroups (Fig 1c); (2) validating PAD difference for consistency across datasets, atlas resolutions, and prediction models in each diagnostic groups; (3) identifying brain patterns associated with the PAD difference in each diagnostic group and evaluating the associations between the PAD and symptoms (Fig 1d); 4) identifying the biological function of genes relatively over or under expressed in association with identified brain patterns (Fig 1e). By using large imaging samples and incorporating both clinical and genetic analyses, we sought to systematically and comprehensively investigate the deviation from normative brain aging and the underlying neuromolecular mechanisms associated with the PAD difference for 9 common brain disorders. We hypothesized that common brain disorders exhibit distinct degrees of accentuated brain aging, each associated with a specific spatial brain pattern linked to unique gene expression profiles.

## Methods

### Ethics statement

For the Human Connectome Project (HCP) dataset, ethical approval was granted by the Washington University Institutional Review Board (IRB, 201204036). The Brain Genomics Superstruct Project (GSP) was approved by the Partners HealthCare IRB and the Harvard University Committee on the Use of Human Subjects in Research. The UK Biobank (UKB) study was conducted under a protocol approved by the North West Multi-centre Research Ethics Committee (reference: 16/NW/0274). All data included in the ADHD-200 dataset were obtained under IRB approvals at the respective contributing institutions, including Peking University (PKU), Kennedy Krieger Institute (KKI), NeuroIMAGE, New York University Child Study Center (NYU), Oregon Health and Science University (OHSU), University of Pittsburgh, Washington University in St. Louis, and Brown University. According to the International Neuroimaging Data-Sharing Initiative (INDI) protocol, no additional IRB approval was required for secondary analyses of de-identified publicly available data. The original studies included in the Autism Brain Imaging Data Exchange (ABIDE II) dataset were approved by the IRBs at each participating site (full site list available at https://fcon_1000.projects.nitrc.org/indi/abide/abide_II.html). In accordance with INDI data usage policies, no additional IRB approval was required for secondary analyses of de-identified publicly available data. The Bipolar-Schizophrenia Network for Intermediate Phenotypes (BSNIP-1) study protocol was approved by the IRBs of Hartford Hospital, the University of Texas Southwestern Medical School, the University of Maryland, the University of Chicago, Wayne State University, and Harvard University. The MDD dataset was approved by the Ethics Committees of Beijing Anding Hospital, West China Hospital of Sichuan University, the First Affiliated Hospital of Zhejiang University, and Henan Mental Hospital of Xinxiang. The AUD and TUD datasets were approved by the University of New Mexico Human Research Review Committee. The Alzheimer’s Disease Neuroimaging Initiative (ADNI) protocol was approved by the Institutional Review Boards of all participating institutions. Detailed information regarding the participating ethics committees and institutional review boards is available at https://adni.loni.usc.edu/about/governance/#core-details. Written informed consents were obtained after providing a complete description of the research process to each participant. For participants younger than 18 years of age, written informed consent was obtained from their parents or legal guardians, with assent obtained from the participants where applicable.

### Participants

Neuroimaging data for the training of brain age prediction were obtained from 3 consortia, including HCP (*n* = 2,489) [16], GSP (*n* = 1,034) [17], and UKB (*n* = 39,679) [18]. ADHD (*n* = 344) and age-gender-quantity matched HC (*n* = 344) were obtained from ADHD-200 project [19]; ASD (*n* = 484) and matched HC (*n* = 484) from ABIDE II [20]; SZ (*n* = 152) and matched HC (*n* = 152), BP (*n* = 143) and matched HC (*n* = 143) from BSNIP-1 [21]; MDD (*n* = 258) and matched HC (*n* = 258) from Beijing Anding Hospital, West China Hospital of Sichuan, First Affiliated Hospital of Zhejiang and Henan Mental Hospital of Xinxiang [22]; AUD (*n* = 155) and TUD (*n* = 144) from Albuquerque [23]; AD (*n* = 361) and matched HC (*n* = 361), MCI (*n* = 657) and matched HC (*n* = 657) from ADNI [24]. The diagnosis of ADHD, ASD, SZ, BP, and MDD were based on the Structured Clinical Interview for Diagnostic and Statistical Manual of Mental Disorders (DSM-IV) [25]. Participants with Alcohol Use Disorder Identification Test (AUDIT) > 7/Fagerström Test for Nicotine Dependence (FTND) > 7 were categorized as AUD/TUD [26,27] (A&TUD were from AUD and TUD with AUDIT > 7 and FTND > 7). The diagnosis of MCI and AD was based on the National Institute of Neurological and Communicative Disorders and Stroke and the Alzheimer’s Disease and Related Disorders Association (NINCDS/ADRDA) criteria [28]. More detailed information on the diagnostic criteria and medication status for healthy participants and patients can be found in Text A in S1 Appendix. Demographic information of individuals participated in this study can be found in Table 1. The scatterplot of ages used in each control subgroup can be found in Fig A in S3 Appendix. Gray matter volume (GMV) from sMRI generated by Statistical Parametric Mapping version 12 (SPM12) was used for brain age prediction. Details on imaging parameters and preprocessing pipeline can be found in Text B in S1 Appendix and Table A in S2 Appendix. In addition, details on the impact of 1.5T scanner acquisitions on predictive performance and PAD difference can be found in Text C in S1 Appendix and Tables B and C in S2 Appendix. The research program was approved by the institution review board at each local site. Written informed consents were obtained after providing a complete description of the research process to each participant.

**Table 1:** Demographic information of individuals participated in this study.

Datasets	Group	Number	Age: min–max (mean±sd)	Gender (M/F)
**HCP**	HC	*n* = 2,489	5–95(34.23 **±** 19.92)	1126/1363
**GSP**	HC	*n* = 1,034	19–35; (21.53 **±** 2.95)	431/603
**UKB**	HC	*n* = 39,679	44–82; (64.12 **±** 7.54)	18680/20999
**ADHD-200**	ADHD	*n* = 344	7–22; (11.43 **±** 2.86)	268/76
	HC	*n* = 344	7–22; (11.41 **±** 2.62)	271/73
**ABIDE II**	ASD	*n* = 484	5–64; (14.53 **±** 8.63)	408/76
	HC	*n* = 484	5–64; (14.54 **±** 8.99)	401/83
**BSNIP-I**	SZ	*n* = 152	15–64; (34.85 **±** 11.98)	100/52
	BP	*n* = 143	15–65; (36.20 **±** 13.17)	47/96
	HC	*n* = 295	15–65; (35.43 **±** 11.75)	138/157
**Depression**	MDD	*n* = 258	15–64; (32.44 **±** 10.73)	97/161
	HC	*n* = 258	15–64; (32.38 **±** 10.54)	98/160
**Addiction**	AUD	*n* = 155	18–55; (30.20 **±** 8.29)	109/46
	TUD	*n* = 144	18–55; (33.24 **±** 9.35)	96/48
	A&TUD	*n* = 54	21–55; (32.5 **±** 8.70)	40/14
	HC	*n* = 299	18–55; (31.67 **±** 9.34)	203/96
**ADNI**	AD	*n* = 361	55–95; (75.91 **±** 6.82)	195/166
	MCI	*n* = 657	55–95; (74.00 **±** 7.82)	302/355
	HC	*n* = 1,018	55–95; (74.70 **±** 7.00)	481/537

HCP, Human Connectome Project; GSP, Brain Genomics Superstruct Project; UKB, UK Biobank; ABIDE II, Autism Brain Imaging Data Exchange; BSNIP-I, Bipolar and Schizophrenia Network for Intermediate Phenotypes; ADNI, Alzheimer’s Disease Neuroimaging Initiative; ADHD, attention-deficit/hyperactivity disorder; ASD, autism spectrum disorder; SZ, schizophrenia; BP, bipolar disorder; MDD, major depressive disorder; AUD, alcohol use disorder; TUD, tobacco use disorder; AD, Alzheimer’s disease; MCI, mild cognitive impairment; HC, healthy control; M/F, male/female; sd, standard deviation.

### Brain age prediction with age correction

Age-range matched training HCs were selected from HCP, GSP, and UKB (641 for ADHD, 1,061 for ASD, 2,164 for SZ, 2,177 for BP, 2006 for MDD, 1912 for AUD, 1912 for TUD,1805 for A&TUD, 34,308 for AD and MCI) generating 10 training sets for each diagnostic group. Patients were not included in the training or cross-validation procedures. Details on samples construction for training and testing can be found in Fig B in S3 Appendix. The sample overlap between the corresponding training sets of different diagnostic groups can be found in Table D in S2 Appendix. Schaefer atlas [29] with 1,000 cortical regions of interest (ROIs) plus 16 subcortical ROIs from Melbourne subcortical brain atlas [30] were combined to generate 1016-ROIs as the “augmented Schaefer (AS)-1016” [31]. Averaged GMV based on augmented Schaefer-1016 brain atlas were used as features in brain age prediction (Fig 1a). The extreme gradient boosting (XGBoost) algorithm based on gradient boosted decision trees was chosen to predict the brain age, with chronological age of individuals in the healthy training set serving as the prediction target. The parameters were optimized using a 10-fold cross-validation conducted within the healthy training set. The optimal number of training iterations was determined by evaluating the prediction error across 2,000 rounds and early stopping was applied if performance did not improve for 20 consecutive rounds [32]. The hyperparameters of XGBoost were set up with a maximum depth of 4 and learning rate of 0.05, with other parameters set to default. The performance of predictive model was estimated by correlating the predicted brain age and chronological age, as well as by computing the mean absolute error (MAE) and coefficient of determination (*R*^2^), in the healthy training set. After optimization, the trained models were applied to independent patient samples to generate predicted brain age and calculate the PAD (deviation between predicted brain age and chronological age, Fig 1b and 1c).

Accuracy of brain age prediction suffers from an age-dependent bias, which leads to the uncertainty in clinical interpretation [33]. Thus, a bias-adjustment scheme was conducted during brain age prediction [34]. Specifically, this scheme relies on the slope (*α*) and intercept (*β*) of a linear regression model of PAD against chronological age from the training set. For each test sample with chronological age of Ω, the offset is defined in equation (1).


$$\begin{array}{c} offset=\alpha \cdot \Omega +\beta \end{array}$$ (1)

Then, the offset is subtracted from the individual estimated brain age to achieve a bias-free brain age estimation.

### PAD group difference comparison

Group effects of PAD between each patient group and HC were estimated by computing the Cohen’s *d* effect sizes using a linear model [35] accounting for age, age^2^, sex, and site (Fig 1c), as shown in equation (2).


$$\begin{array}{c} d=\frac{t(n_{\text{1}}+n_{\text{2}})}{\sqrt{n_{\text{1}}n_{\text{2}}}\sqrt{df}} \end{array}$$ (2)

where *t* denotes contrast statistics, *n*_1_ and *n*_2_ are the numbers of sample size in two groups, and *df* represents the degrees of freedom used for a corresponding *t* value in a linear model.

### Interpretable brain patterns of PAD

Shapley Additive Explanations (SHAP) [36] is a game-theoretic approach for additive feature attribution that was used to determine the marginal contributions from predictions. Specifically, the changes in model outcome relative to the baseline can be apportioned to individual features, as shown in equation (3).


$$\begin{array}{c} f\left(x_{i}\right)-E_{train}\left[f(x)\right]=\sum_{j=\text{1}}^{M=\text{1}\text{0}\text{1}\text{6}}\emptyset_{ij} \end{array}$$ (3)

where *f*(*x*_*i*_) represents the predicted brain age for a single testing sample *x*_*i*_; *E*_*train*_[*f*(*x*)] represents the fixed baseline (average chronological age in training set); *M* represents the number of input features and $\emptyset_{ij}$ is the SHAP value which represents the contribution of feature *j* to the model prediction of participant *i*. Moreover, to mitigate potential bias arising from correlations among input features, SHAP values were computed using TreeSHAP with the interventional feature perturbation scheme, which anchors the perturbation process in the empirical data distribution rather than relying on the independence assumption used in conventional perturbation strategies, thereby reducing attribution bias when features are correlated [37]. Then, a SHAP value matrix with sample size × number of input features (*n* × *M*) for testing set was generated. We incorporate interaction terms (SHAP × group) in multiple linear regression models to identify the brain features for PAD difference in each diagnostic group, where the age-corrected PAD was the dependent variable, while age, age^2^, sex, site, group, SHAP value, and SHAP × group were the independent variables. Subsequently, PAD difference T-map was obtained according to statistical *t*-values of the interaction coefficien*t*s. The brain regions with significant interaction coefficients (*p*_*FDR*_ < 0.05) were considered as the brain regions associated with the PAD difference (Fig 1d).

### Correlation between PAD and symptoms

The linear models accounting for age, age^2^, sex, and site were used to correlate PAD with symptoms in patient groups. Symptom scores are available for SZ including positive and negative scores of the Positive and Negative Syndrome Scale (PANSS); BP including Montgomery-Asberg Depression Rating Scale (MADRS) and Young Mania Rating Scale (YMRS); MDD including Hamilton Depression Rating Scale (HDRS); AUD including AUDIT; TUD including FTND, and AD and MCI including the Mini-Mental State Examination (MMSE). Then, the *t*-statistics of the linear models were converted to *r*, as shown in equation (4).


$$\begin{array}{c} =\frac{t}{\sqrt{t^{\text{2}}+df}} \end{array}$$ (4)

where *df* represents the degrees of freedom used for a corresponding *t* value in the linear model. Thus, correlation taking covariates into account reflect a partial correlation between PAD and symptoms.

### Gene enrichment analyses

Post-mortem gene expression data from Allen Institute for Brain Science (AIBS) and PAD difference T-map were used to identify the biological function of genes relatively over- or underexpressed in association with this brain pattern. Specifically, we processed the regional microarray expression data from the Allen Human Brain Atlas (AHBA) [38] using the abagen toolbox (https://github.com/netneurolab/abagen) to map the transcriptomic data onto 1,016 parcellated brain regions from the augmented Schaefer-1016 (details on transcription data processing can be found in Text D in S1 Appendix) to generate regional gene expression matrix (1,016 ROIs × 15,632 genes) as the predictive variable. PAD difference T-map (1,016 ROIs × 1) of each diagnostic group was used as response variable to identify the gene expression component related to these T-maps by partial least square (PLS) regression. The statistical significance of the variance explained by each component was assessed by permuting the response variables 1,000 times. The significance component with the highest explained variance for PAD difference T-map (PLS_T_) was selected for subsequent analysis. The error in estimating PLS_T_ weight for each gene was evaluated by bootstrapping, and the ratio of each gene’s weight to its bootstrap standard error was used to calculate the *Z* scores. Genes with *Z* > 3 and *Z* <−3 (*p*_*FDR*_ < 0.05) were defined as PLS_T_+ and PLS_T_− gene sets, respectively. Metascape [39] (https://metascape.org/gp/index.html) was used to identify gene ontology (GO) terms that significantly enriched in these gene sets. Redundancy elimination and visualization of GO term lists (REViGO) [40] was used to summarize and visualize gene ontology (http://revigo.irb.hr) by removing redundant terms (Fig 1e).

### Artificial intelligence statement

We confirm that no artificial intelligence tools or technologies were used in the conduct of the study or in the preparation of the article content.

This study is reported as per the Strengthening the Reporting of Observational Studies in Epidemiology (STROBE) guideline (S1 Checklist). This study had no prospective protocol or analysis plan.

## Results

### The performance of brain age prediction

The sample size and age distributions of training and testing sets for each diagnostic group are shown in Fig 2a, with no group differences (*p* = 0.79–0.99, two-sample *t* test) in age distribution between patient and HC. For each diagnostic group, an independent XGBoost model was built to predict brain age from averaged GMV extracted from augmented Schaefer-1016 brain atlas (1,000 cortical and 16 subcortical ROIs) through 10-fold cross-validation (details can be found in “Brain age prediction with age correction” section). Results showed excellent predictive performance of the model (without age correction: *r* = 0.68–0.88, mean absolute error-MAE = 1.65–4.97, coefficient of determination-*R*^2^ = 0.45–0.76; after age correction: *r* = 0.89–0.93, MAE = 1.15–3.75, *R*^2^ = 0.76–0.84, Fig C in S3 Appendix), with consistently high performance and low coefficient of variation across folds (without age correction:0.9%–12.1%, after age correction:0.5%–10.5%, Tables E and F in S2 Appendix, details can be found in Text E in S1 Appendix), indicating the effectiveness of the brain age prediction model and highlighting the necessity of age-bias correction. Then, the group-specific XGBoost models were applied to the corresponding independent 9 diagnostic groups to generate the predicted brain age, which was then correlated with age. Results showed that the correlation between predicted brain age and chronological age, as well as the MAE and *R*^2^, were consistently better in HC than in patients across all clinical groups (*r* = 0.75, MAE = 1.56, *R*^2^ = 0.57 for ADHD, and *r* = 0.77 MAE = 1.37, *R*^2^ = 0.60 for HC; *r* = 0.82, MAE = 3.26, *R*^2^ = 0.63 for ASD and *r* = 0.83, MAE = 3.09, *R*^2^ = 0.67 for HC; *r* = 0.85, MAE = 6.10, *R*^2^ = 0.59 for SZ and *r* = 0.89, MAE = 4.56, *R*^2^ = 0.75 for HC; *r* = 0.88, MAE = 5.21, *R*^2^ = 0.72 for BP and *r* = 0.89, MAE = 4.56, *R*^2^ = 0.75 for HC; *r* = 0.79, MAE = 5.24, *R*^2^ = 0.58 for MDD and *r* = 0.83, MAE = 4.64, *R*^2^ = 0.66 for HC; *r* = 0.75, MAE = 4.52, *R*^2^ = 0.51 for AUD and *r* = 0.89, MAE = 3.60, *R*^2^ = 0.75 for HC; *r* = 0.79, MAE = 4.67, *R*^2^ = 0.59 for TUD and *r* = 0.89, MAE = 4.26, *R*^2^ = 0.69 for HC; *r* = 0.79, MAE = 4.74, *R*^2^ = 0.52 for A&TUD and *r* = 0.84, MAE = 3.22, *R*^2^ = 0.70 for HC; *r* = 0.88, MAE = 3.36, *R*^2^ = 0.71 for AD and *r* = 0.90, MAE = 2.59, *R*^2^ = 0.77 for HC; *r* = 0.89, MAE = 2.81, *R*^2^ = 0.70 for MCI and *r* = 0.91, MAE = 2.59, *R*^2^ = 0.79 for HC, Fig 2b). We also investigated the impact of sample size on the performance of brain age prediction and observed that the larger training sample size the better performance and the smaller variability (Fig D in S3 Appendix).

### PAD difference for each diagnostic group

**Fig 3:**
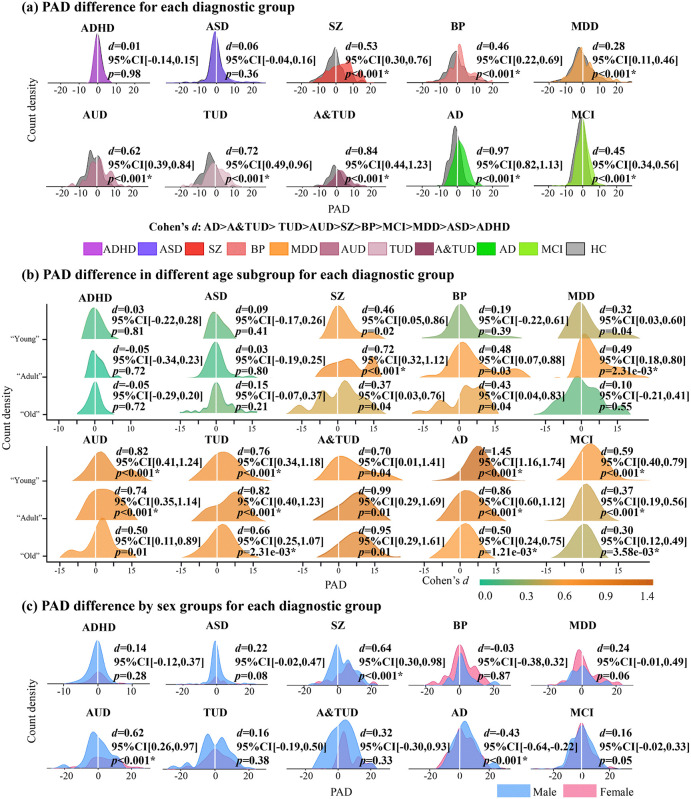
PAD difference by diagnosis, age, and sex. **(a)** PAD difference between patient and HC. Cohen’s *d* accounting for age, age^2^, sex and site, 95% confidence interval (CI), and two-sided *p* values are provided. (**b)** PAD difference between patient and HC in different age subgroups. **(c)** PAD difference between male and female. All *p* values are calculated using linear model-based *t*-tests. (*) is used to indicate results which pass false discovery rate (FDR) correction for multiple comparisons. ADHD, attention-deficit/hyperactivity disorder; ASD, autism spectrum disorder; SZ, schizophrenia; BP, bipolar disorder; MDD, major depressive disorder; AUD, alcohol use disorder; TUD, tobacco use disorder; A&TUD, AUD and TUD; AD, Alzheimer’s disease; MCI, mild cognitive impairment; HC, healthy control; CI, confidence interval.

The PAD difference between patient and HC was calculated using linear regression accounting for age, age^2^, sex, and site as covariates. The PAD was higher in all the diagnostic groups than HC (Fig 3a), in which strong difference effects was observed in AD (*d* = 0.97, 95% confidence interval (CI) [0.82,1.13]; *p* < 0.001*) and A&TUD (*d* = 0.84, 95% CI [0.44,1.23]; *p* < 0.001*), moderate effects in TUD (*d* = 0.72, 95% CI [0.49,0.96]; *p* < 0.001*), AUD (*d* = 0.62, 95% CI [0.39,0.84]; *p* < 0.001*), SZ (*d* = 0.53, 95% CI [0.30,0.76]; *p* < 0.001*), BP (*d* = 0.46, 95% CI [0.22,0.69]; *p* < 0.001*) and MCI (*d* = 0.45, 95% CI [0.34,0.56]; *p* < 0.001*), small effects in MDD (*d* = 0.28, 95% CI [0.11,0.46]; *p* < 0.001*), and negligible effects in ASD (*d* = 0.06, 95% CI [−0.04,0.16]; *p* = 0.36) and ADHD (*d* = 0.01, 95% CI [−0.14,0.15]; *p* = 0.98). In addition, fraction am*p*litude of low-frequency fluctuation (fALFF) from functional MRI (fMRI) was also used to predict brain age under the same prediction framework (Details on fMRI preprocessing pipeline can be found in Text B in S1 Appendix). Results showed that no PAD difference was observed in almost all the diagnostic groups (Fig E in S3 Appendix).

The PAD difference between patient and HC in different age range was also investigated by dividing into “young,” “adult,” and “old” subgroups (Fig F in S3 Appendix and Tables G–I in S2 Appendix). Results (Fig 3b) showed that increased PAD relative to HC was most prominently observed in “young” dementia (including AD: *d* = 1.45, 95% CI [1.16,1.74]; *p* < 0.001* and MCI: *d* = 0.59, 95% CI [0.40,0.79]; *p* < 0.001*) and AUD (*d* = 0.82, 95% CI [0.41,1.24]; *p* < 0.001*), and “adult” psychiatric disorders (including SZ: *d* = 0.72, 95% CI [0.32,1.12]; *p* < 0.001*, BP: *d* = 0.48, 95% CI [0.07,0.88]; *p* = 0.03 and MDD: *d* = 0.49, 95% CI [0.18,0.80]; *p* = 2.31e − 03*) and addiction (including TUD: *d* = 0.82, 95% CI [0.40,1.23]; *p* < 0.001* and A&TUD: *d* = 0.99, 95% CI [0.29,1.69]; *p* = 0.01), with no PAD difference in “young” BP (*d* = 0.19, 95% CI [−0.22,0.61]; *p* = 0.39), “old” MDD (*d* = 0.10, 95% CI [−0.21,0.41]; *p* = 0.55) and develo*p*mental disorders (including ADHD and ASD). We further examined age × group interaction effects on PAD (details can be found in Text F in S1 Appendix) and identified significant interactions for AD and MCI (Table J in S2 Appendix), with estimated divergence ages of approximately 57 years for AD and 68 and 80 years for MCI (Fig G in S3 Appendix). Moreover, PAD difference by sex was also calculated. Results (Fig 3c) showed that female PAD was significantly higher than male in SZ (*d* = 0.64, 95% CI [0.30,0.98]; *p* < 0.001*) and AUD (*d* = 0.62, 95% CI [0.26,0.97]; *p* < 0.001*), while lower than male in AD (*d*=−0.43, 95% CI [−0.64, −0.22]; *p* < 0.001*), with no sex difference in other brain disorders.

### PAD difference sequence pattern validations across datasets, atlas resolutions, and prediction models

**Fig 4:**
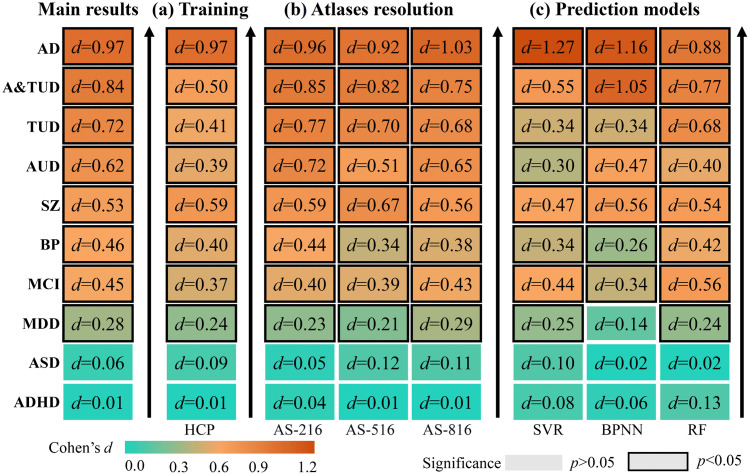
PAD difference sequence pattern validation across datasets, atlas resolutions, and different prediction models. **(a)** Full age range HCP was used as training sets to test PAD difference. **(b)** PAD difference under different scales of brain atlas, where Schaefer-216/516/816 were used to extract GMV. **(c)** PAD difference across different prediction models, where SVR, BPNN, and RF were used to predict brain age. Color bar indicates Cohen’s *d* accounting for age, age^2^, sex, and site. All *p* values are calculated using linear model-based *t*-tests. Significant differences (*p* < 0.05) are marked with a black box. HCP, Human Connectome Project; AS, augmented Schaefer; SVR, support vector regression; BPNN, back propagation neural network; RF, random forest; ADHD, attention-deficit/hyperactivity disorder; ASD, autism spectrum disorder; SZ, schizophrenia; BP, bipolar disorder; MDD, major depressive disorder; AUD, alcohol use disorder; TUD, tobacco use disorder; A&TUD, AUD and TUD; AD, Alzheimer’s disease; MCI, mild cognitive impairment.

We validated the robustness of PAD difference sequence pattern among diagnostic categories (dementia > addiction > psychiatric disorders > developmental disorders) when using different training sets (full age range HCP), scales of brain atlas (Schaefer-216/516/816) and prediction models (support vector regression: SVR; back propagation neural network: BPNN; and random forest: RF). Details on the implementation of all these datasets, atlases, and predictive models can be found in Text G in S1 Appendix. Ten-fold cross-validation confirmed the predictive performance of all models (Fig H in S3 Appendix), which were used to predict the brain age in the diagnostic groups, followed by calculating the PAD difference between patient and HC. The Spearman rank correlation coefficient *β* was used to evaluate the consistency of the PAD difference sequence pattern among diagnostic categories across datasets, brain atlas resolutions, and prediction models (details can be found in Text H in S1 Appendix). Results showed that the identified PAD difference sequence pattern among diagnostic group were highly consistent across training sets (*β* = 0.90, *p* = 8.80e − 04, Fig 4a and Table K in S2 Appendix), brain atlas resolutions (*β* = 1.00/0.98/0.99 and all *p* < 1.00e − 16 for AS-216/516/816, Fig 4b and Table K in S2 Appendix) and prediction models (*β* = 0.83/0.91/0.88 and *p* = 3.18e − 03/3.07e − 04/1.98e − 03 for SVR/BPNN/RF, Fig 4c and Table K in S2 Appendix).

### Brain patterns associated with the PAD difference

**Fig 5:**
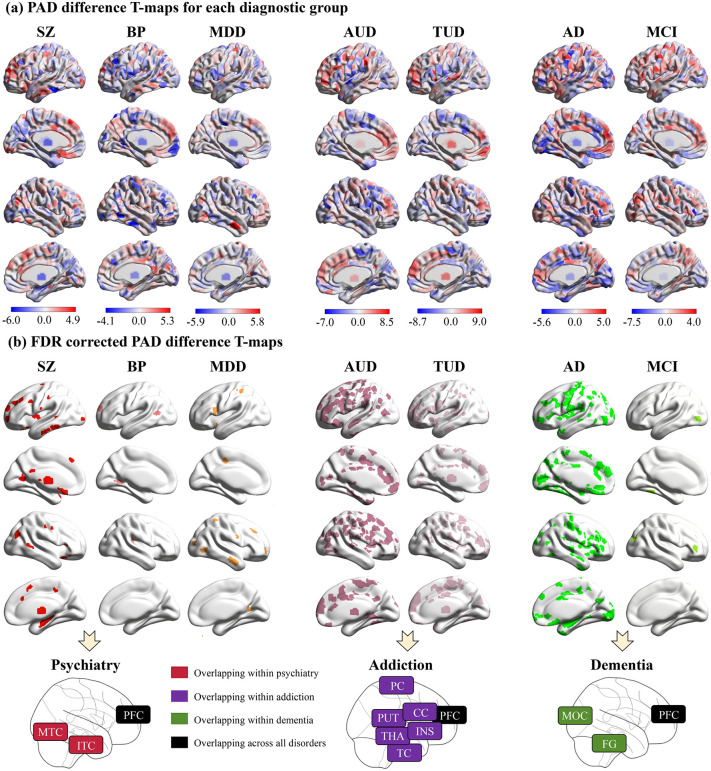
Brain patterns associated with the PAD difference. **(a)** PAD difference T-maps for each diagnostic group. Color bar indicates statistical *t*-values of the interaction coefficients, and red/blue regions represent stronger positive/negative associations between the contribution of the identified brain features to predicting brain age and PAD in patient groups than in the HC. **(b)** The FDR corrected PAD difference T-maps. The deep red, deep purple, deep green, and black box represent the shared brain regions within psychiatric disorders (including SZ, BP, and MDD), addiction (including AUD and TUD), dementia (including AD and MCI), and among all patient groups. SZ, schizophrenia; BP, bipolar disorder; MDD, major depressive disorder; AUD, alcohol use disorder; TUD, tobacco use disorder; AD, Alzheimer’s disease; MCI, mild cognitive impairment; PAD, predicted age difference; FDR, false discovery rate; MTC, middle temporal cortex; ITC, inferior temporal cortex; PFC, prefrontal cortex; PC, precentral cortex; CC, cingulate cortex; PUT, putamen; INS, insula; THA, thalamus; TC, temporal cortex; MOC, middle occipital cortex; FG, fusiform gyrus.

To identify brain features contributed to PAD difference in each diagnostic group (ADHD and ASD were excluded due to no PAD difference, A&TUD were excluded to prevent redundancy), we first calculated the SHAP value for each of the 1,016 features. Then, we estimated a multiple linear regression model with age-corrected PAD as the dependent variable, and age, age^2^, sex, site, group, SHAP value, and the interaction term SHAP × group as independent variables. The SHAP × group interaction captures the influence of each brain feature on PAD difference between patient and HC, and PAD difference T-maps were derived from the t-statistics of the corresponding interaction coefficients. PAD difference T-maps for each diagnostic group were displayed in Fig 5a with false discovery rate (FDR) corrected in Fig 5b, whose reproducibility across different scales of brain atlas was validated (Fig I in S3 Appendix). Prefrontal cortex (PFC), middle temporal cortex (MTC), and inferior temporal cortex (ITC) were the shared brain regions with significant SHAP × group interaction within psychiatric group. PFC, superior/middle/inferior temporal cortex (STC/MTC/ITC), superior/middle occipital cortex (SOC/MOC), thalamus, and insula were identified in SZ; PFC, MTC, ITC, and lingual gyrus (LG) in BP; and PFC, MTC, ITC, and MOC in MDD. Within addiction group, PFC, precentral cortex (PC), cingulate cortex (CC), insula, putamen, thalamus, and temporal cortex (TC) were the identified shared brain regions. PFC, PC, CC, insula, putamen, thalamus, STC, MTC, ITC, MOC, inferior occipital cortex (IOC), LG, superior parietal cortex (SPC), and fusiform gyrus (FG) were identified in AUD; PFC, PC, CC, insula, putamen, thalamus, STC, MTC, and ITC in TUD. Within dementia group, PFC, MOC, and FG were the shared brain regions. PFC, SOC, MOC, FG, hippocampus, para-hippocampus, MTC, ITC, PC, anterior cingulate cortex (ACC), amygdala, caudate, insula, and thalamus were identified in AD; PFC, MOC, and FG for MCI. The identified brain regions are summarized in Tables L–N in S2 Appendix. Note that PFC was consistently identified for PAD difference across psychiatric disorders, addiction, and dementia. Moreover, the bootstrapping analysis showed that the identified brain features consistently exhibited high selection frequency across 1,000 resampling conditions, indicating their robustness and stability (details on bootstrapping analysis can be found in Text I in S1 Appendix and Fig J in S3 Appendix).

### Relationships between PAD and symptoms

**Fig 6:**
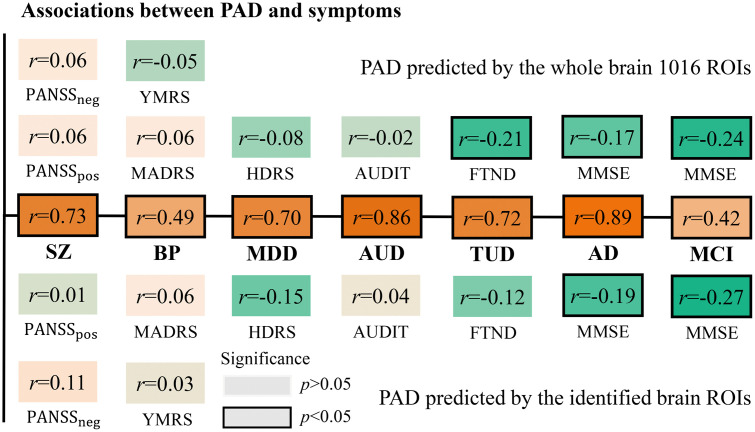
Correlation between PAD and symptoms. Upper: correlation between the whole brain PAD (predicted by the whole brain 1,016 ROIs) and symptoms. Bottom: correlation between the identified brain PAD (predicted by the identified brain ROIs) and symptoms. All *p* values are calculated using linear model-based *t*-tests. Significant associations (*p* < 0.05) are marked with a black box. SZ, schizophrenia; BP, bipolar disorder; MDD, major depressive disorder; AUD, alcohol use disorder; TUD, tobacco use disorder; AD, Alzheimer’s disease; MCI, mild cognitive impairment; PAD, predicted age difference; ROIs, regions of interest; PANSS_pos/neg_, positive and negative scores of the Positive and Negative Syndrome Scale; MADRS, Montgomery-Asberg Depression Rating Scale; YMRS, Young Mania Rating Scale; HDRS, Hamilton Depression Rating Scale; AUDIT, Alcohol Use Disorder Identification Test; FTND, Fagerström Test for Nicotine Dependence; MMSE, Mini‐Mental State Examination.

Partial correlation between PAD and symptoms in each diagnostic group were calculated by linear models accounting for age, age^2^, sex, and site. Results (Fig 6) showed that PAD was negatively correlated with MMSE in AD (*r*=−0.17, *p* = 0.02) and MCI (*r*=−0.24, *p* = 8.12e − 06), and with FTND in TUD (*r*=−0.21, *p* = 0.01). However, no association (*p* > 0.05) was observed between PAD and symptom in other brain disorders (including SZ, BP, MDD, and AUD). The high degree of correlations between the whole brain PAD (predicted by the whole brain 1,016 ROIs) and the identified brain PAD (predicted by the identified brain ROIs) in each diagnostic group (*r* = 0.73 for SZ, *r* = 0.49 for BP, *r* = 0.70 for MDD, *r* = 0.86 for AUD, *r* = 0.72 for TUD, *r* = 0.89 for AD, *r* = 0.42 for MCI) confirmed the effectiveness of the identified regions. The associations between the whole brain PAD and symptoms were largely replicated in the identified brain PAD, with negative association with MMSE in AD (*r*=−0.19, *p* = 5.52e − 03) and MCI (*r*=−0.27, *p* = 9.13e − 07) and no association in other brain disorders.

### Enrichment analyses of genes related to PAD difference T-map

**Fig 7:**
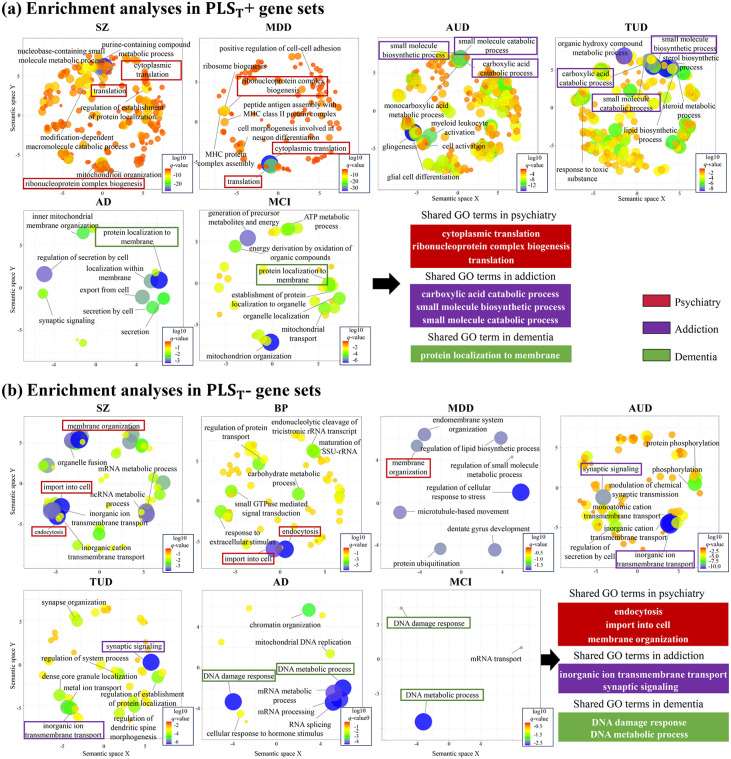
Significant GO terms for biological processes related to PAD difference T-maps. Significant GO terms for biological processes that the PLS_T_+ **(a)** and PLS_T_− **(b)** gene sets in each diagnostic group enriched were plotted in semantic space so that similar terms were represented close to each other. The top 8 significant GO terms were labeled for each analysis with redundant GO terms excluded by REViGO. Markers were scaled based on the log10 *q*-value (*q* represents the *p*-value after FDR correction) for the significance of each GO term. Large blue circles were highly significant, while small red circles were less so. SZ, schizophrenia; BP, bipolar disorder; MDD, major depressive disorder; AUD, alcohol use disorder; TUD, tobacco use disorder; AD, Alzheimer’s disease; MCI, mild cognitive impairment; GO, gene ontology.

The enrichment analyses (details can be found in the “Gene enrichment analyses” section) were conducted to identify the biological function of genes relatively over- or underexpressed in association with PAD difference T-map. The first (the third for MDD) partial least squares (PLS) component explained 8.1%, 5.4%, 5.1%, 7.2%, 7.0%, 6.3%, and 5.3% variance in SZ, BP, MDD, AUD, TUD, AD, and MCI, which were not observed by chance (permutation test, *p* = 0.006 for SZ, *p* = 0.014 for BP, *p* = 0.048 for MDD, *p* = 0.019 for AUD, *p* = 0.046 for TUD, *p* = 0.047 for AD, and *p* = 0.048 for MCI, Fig K in S3 Appendix). PLS1 (PLS3 for MDD) scores were positively correlated with the PAD difference T-map in SZ (spatial permutation tests, *r* = 0.24, *p*_*spin*_ = 0.002), BP (*r* = 0.21, *p*_*spin*_ < 0.001), MDD (*r* = 0.22, *p*_*spin*_ < 0.001), AUD (*r* = 0.21, *p*_*spin*_ = 0.002), TUD (*r* = 0.22, *p*_*spin*_ = 0.001), AD (*r* = 0.23, *p*_*spin*_ < 0.001), and MCI (*r* = 0.22, *p*_*spin*_ < 0.001), respectively (Fig L in S3 Appendix). After correcting for enrichment terms (*p*_*FDR*_ < 0.05) and removing redundant terms, the significant Gene Ontology (GO) biological processes for PLS_T_+ and PLS_T_− gene sets enriched were shown in Fig 7a and 7b, in which no significant GO terms enriched in PLS_T_+ gene set for BP. The top 8 significant GO terms (Tables O and P in S2 Appendix) were labeled for each diagnostic group (only 3 significant GO terms were identified in PLS_T_− gene set for MCI). In PLS_T_ + , the cytoplasmic translation, ribonucleoprotein complex biogenesis, and translation were the shared significant GO terms in psychiatric disorders (SZ and MDD); carboxylic acid catabolic process, small molecule biosynthetic process, and small molecule catabolic process were the shared in addiction (AUD and TUD); protein localization to membrane was the shared in dementia (AD and MCI). For PLS_T_ − , the endocytosis, import into cell and membrane organization were the shared in psychiatric disorders (SZ, BP, and MDD); inorganic ion transmembrane transport and synaptic signaling were the shared in addiction (AUD and TUD); deoxyribonucleic acid (DNA) damage response and DNA metabolic process were the shared in dementia (AD and MCI).

## Discussion

By combining large structural neuroimaging datasets, we found strong evidence that PAD was consistently larger in patients than in HC across diagnostic groups. Different brain disorders exhibited different degrees of aging, with the highest effects in dementia, followed by addiction and psychiatric disorders, but not in developmental disorders (dementia > addiction > psychiatric disorders > developmental disorders). These results were highly replicable across different training sets, brain atlas resolutions, and prediction models. The regional brain differences underlying PAD among psychiatric disorders (PFC, MTC, and ITC), addiction (PFC, PC, CC, insula, putamen, thalamus, and TC), and dementia (PFC, MOC, and FG) were associated with specific spatial brain patterns, but also with a shared region in PFC. Furthermore, we also found that the PAD was correlated with symptom in dementia, but not in addiction and psychiatric disorders. Gene enrichment analyses suggested that the gene sets related to brain patterns associated with the PAD difference in common brain disorders were enriched in different biological processes. This study represents an integrative effort to systematically compare PAD differences across 9 common brain disorders, and identify the brain patterns associated with these differences and the biological function of its associated genes, using relatively large datasets within a unified framework. This approach may help to disentangle the pathophysiological complexity of brain disorders from the perspective of brain aging.

The reliability of brain age prediction is a crucial influence on the validity of results regarding deviations from normal aging in common brain disorders. Lack of model accuracy, along with insufficient data quality or quantity, can introduce bias, causing PAD to reflect noise rather than true physiological variation [32]. In our study, the reliability of brain age prediction was aided by the large dataset, that incorporated several prominent neuroimaging datasets for model training, including HCP, GSP, and UKB. By encompassing a wide range of brain disorders, our investigation is one of the most comprehensive efforts in brain age prediction to date. Additionally, by comparing PAD in patients to age-, sex-, and quantity-matched controls, we minimized the influence of noise from model inaccuracies or data quality, thereby allowing us to better attribute the PAD to biologically relevant differences.

The different degrees of accentuated aging in different brain disorders are largely consistent with meta-analyses considering multiple brain disorders which reported the strongest effects in neurodegenerative disorders, especially AD, followed by psychiatric disorders where relatively more pronounced brain aging was measured in SZ than BP and MDD, and without case-control differences in PAD for developmental disorders (including ADHD and ASD) [32,41,42]. Prior neuroimaging research has reported significant accentuated brain aging in AUD and TUD, reflecting age-related GMV atrophy patterns similar to AD [43–45]. We have extended these reports by documenting the replicability of PAD difference across different datasets, brain atlas resolutions and prediction models, providing powerful supporting evidence for both the universality of accentuated brain aging among brain disorders and the specificity across individual disorders. Notably, the observation that the PAD pattern (dementia > addiction > psychiatric disorders > developmental disorders) largely mirrors the mean group ages of these diagnostic categories may reflect the interaction between aging-related brain vulnerability and disease processes. As the brain ages, age-related structural and physiological changes may reduce neural resilience, such that pathological processes superimposed on an aging neural substrate may exert amplified effects on brain structure [46]. In contrast, developmental disorders emerge during childhood or adolescence, a period characterized by heightened neuroplasticity, ongoing myelination, and robust compensatory mechanisms [47,48]. The developing brain possesses greater synaptic exuberance and more efficient repair and reorganization capabilities, which may buffer against the structural impact of disorder-related pathology. Moreover, fALFF-based features failed to detect PAD difference between patients and HC, suggesting that the brain aging patterns may be more sensitive to structural than to functional alterations. In addition, different age stages in common brain disorders exhibit varying degrees of accentuated aging patterns, suggesting that the effects of brain aging may vary across the stages of disorder progression, emphasizing the heterogeneous aging subgroups in common brain disorders. We found sex difference in brain aging measures associated with brain disorders, as previously reported [49], suggesting the critical importance of considering sex in disease investigations. We also observed significant correlations between PAD and symptom in dementia (including AD and MCI), but not in other disorders, suggesting that the progression of dementia may co-occur with and be influenced by accentuated brain aging, while the development of symptoms related to addiction and psychiatric disorders may be more closely associated with the neuropathology inherent to those conditions.

The shared brain regions associated with accentuated aging were frontotemporal cortex in psychiatric disorders; default mode network (DMN), salience network (SAN) and putamen-thalamus in addiction; and fronto-occipital cortex in dementia. GMV reduction in frontotemporal cortex has been widely reported in psychiatric disorders as well as being associated with usual aging [50–52]. Previous studies investigating the interaction of alcohol/tobacco use and aging on brain morphology have reported whole brain GMV decreases with age among both HC and AUD/TUD, but with greater GMV reductions in core brain regions of DMN and SAN, including PFC, PC, CC, insula, and TC for AUD/TUD [44,53–55]. In addition, aging effects in putamen and thalamus have been consistently reported in AUD and TUD [43,56]. Previous studies have reported local changes in the PFC, MOC, and FG in the early stages of dementia, with these abnormalities progressively extending to encompass broader brain regions as the disease advances, ultimately involving the entire brain [57,58], consistent with our results. Our results suggested that frontotemporal network, DMN-SAN-putamen-thalamus and fronto-occipital network were overlapping aging and disease-related brain variations captured by the premature aging hypothesis, which may serve as potential biomarkers for assessing the risk and progression of psychiatric disorders, addiction, and dementia, respectively. More importantly, when combining all brain disorders, aging, and disease-related brain variations in PFC were consistently identified across psychiatric disorders, addiction, and dementia, demonstrating that PFC was involved in both these processes. This dual sensitivity to aging and disease across brain disorders further indicates that common brain disorders might not be completely independent at the level of neuroanatomical phenotype associated with aging. If so, this may provide valuable insights into the underlying neural mechanisms contributing to the substantial epidemiological comorbidity observed among common brain disorders, from the perspective of brain aging.

Enrichment analyses identified the biological functions of relatively over- or underexpressed genes associated with brain patterns linked to PAD difference in common brain disorders. These functions included: translation and transport in psychiatric disorders; catabolic processes, transmembrane transport and synaptic signaling in addiction; and cellular protein localization and DNA damage response and DNA metabolic process in dementia. These findings are partially consistent with the prior literature. A previous study reported that aberrant dendritic spine morphology and function regulated by translation initiating genes were associated with psychiatric disorders, including SZ, BP, and MDD [59,60]. Moreover, psychiatric disorders are linked to membrane and receptor transport, with endocytosis being the primary mechanism governing membrane organization transport, and abnormalities in these processes have been reported [61]. The catabolic process involves the breakdown of substances to release energy utilized by cells and organisms, however, alcohol and tobacco use can significantly disrupt the homeostasis of energy metabolism at both the cellular and whole-body levels, leading to tissue damage [62,63]. Additionally, alcohol and tobacco use can significantly alter ion channel and receptor functions on neuronal membranes, leading to modified neurotransmitter release and receptor expression, particularly affecting synaptic signaling in dopamine and glutamate pathways [64,65]. These alterations not only reinforce addictive behaviors but also induce changes in neural plasticity, thereby solidifying long-term dependence [64]. The abnormal aggregation and mis-localization of amyloid β and Tau proteins have been reported in dementia, which can lead to neurotoxicity and synaptic dysfunction, thereby affecting neuronal health and cognitive function [66]. Spontaneous DNA damage arise from both endogenous sources (such as DNA metabolism) and exposure to exogenous damage-inducing agents, and the vast majority of these lesions are efficiently removed by conserved DNA repair systems that specifically recognize such damage [67]. However, many studies have reported the accumulation of unrepaired DNA damage during natural aging but also occurs at high levels during neurodegeneration due to defects in DNA damage response, ultimately leading to dementia [68]. Hence, our results suggested that the different biological processes identified in psychiatric disorders, addiction and dementia can explain the accentuated aging phenomenon driven by brain structural abnormalities in corresponding diseases, which may be the shared molecular genetic mechanisms between brain age gaps and brain disorders.

The main finding in our study, that neurodegenerative disorders have the largest PAD is consistent with the post-mortem literature documenting widespread neuronal loss, gliosis, and cortical thinning in dementing illness [69]. Addictions such as AUD and TUD involve toxic agents that result in neuronal injury and brain shrinkage [70], hence their PAD are similarly large. Post-mortem studies of SZ, BP, and MDD do not document universal evidence of neuronal loss/gliosis and their moderately increased PAD values maybe reflect more subtle neuropil retractions limited to fronto-temporal regions [50]. Finally, neuroimaging studies have shown that the differences in cortical structure vary with developmental stage in ADHD and ASD [71,72], which may be a potential reason for minimally changed PAD observed in this study.

Several limitations should be considered. One is that all the datasets included in this study were collected from multiple sites and brain age prediction models were trained on pooled multi-site data without explicit control for site effects at the prediction stage. However, the deconfounding approach, leave-one-site-out (LOSO) validation and site difference analysis of PAD demonstrate that site is not a major confounding factor for brain age prediction in this study, and that the observed PAD differences are unlikely to be driven by site-related acquisition effects (details on deconfounding, LOSO validation, and site difference analysis can be found in Text J in S1 Appendix, Figs M–O in S3 Appendix, and Tables Q–U in S2 Appendix). In addition, site and other confounding factors, including age, age^2^, and sex, were included in all statistical models, testing for group differences and clinical associations to further minimize confounding effects. Second, we processed a substantial number of cross-sectional MRI to evaluate the dynamic PAD difference across various age ranges within each diagnostic group. We acknowledge that using cross-sectional data to analyze a dynamic process is not optimal. However, previous studies demonstrated that cross-sectional and longitudinal approaches produce similar age-related patterns in both normal aging and brain disorders [73]. Third, psychiatric disorders, such as SZ, BP, and MDD have very large co-morbidities with AUD and TUD [74]. We did not account for these potential confounding effects which are well known to affect PAD. Fourth, the age-stratified analyses relied on data-driven subgroups, which may introduce arbitrary age boundaries, reduce statistical power, and complicate biological interpretation. Therefore, these analyses should be interpreted as exploratory. Fifth, although the use of age-range matched HC and disorder-specific training sets may reduce extrapolation error, it may also potentially limit model generalizability and hindering the comparability of PAD estimates across diagnostic groups. However, our site-wise performance analyses indicated excellent cross-site generalizability (Table S in S2 Appendix), and diagnostic group differences were evaluated using standardized effect sizes (Cohen’s *d*) rather than direct comparisons of PAD values. On the other hand, aligning the age range between training and testing sets may, to some extent, bring their distributions closer, which could reduce the strictness of out-of-distribution evaluation and therefore only provides limited assurance that PAD estimates remain unbiased under more heterogeneous clinical samples and acquisition conditions. Although the site difference analysis of PAD provides supporting evidence for a certain degree of out-of-distribution generalization capability, these results should still be interpreted with caution, as it does not fully capture more extreme distribution shifts that may exist in real-world clinical settings. Sixth, although TreeSHAP with interventional perturbation was used to mitigate bias introduced by multicollinearity, and bootstrapping analysis confirmed the robustness of the identified brain regions, considering the inherent spatial dependence among input features, the identified brain regions associated with PAD difference may be exploratory findings for the broad spatial patterns and should be interpreted with caution. Moreover, although demographic-matched data sets and additional covariate adjustments were used to reduce potential confounding effects, other demographic and biological factors, such as ethnicity, non-psychiatric chronic diseases, lifestyle behaviors, and environmental exposures, were not consistently available across all datasets and therefore could not be explicitly modeled in the present study. Consequently, residual confounding effects cannot be fully excluded, and future studies with more comprehensive phenotypic harmonization are needed. Finally, the gene expression analyses were based on post-mortem transcriptomic data from a limited number of donors (*n* = 6), as transcriptomic data for the brain disorder cohorts included in this study were not available, which may limit the generalizability of these findings.

Collectively, the present study utilized large datasets to evaluate PAD difference across multiple brain disorders compared to HC, and identified brain patterns associated with the PAD difference, as well as the biological functions of associated genes. The PAD was increased in common brain disorders, with the greatest effects in dementia, followed by addiction and psychiatric disorders, and no measurable effect in developmental disorders. Each brain disorder showed a specific brain pattern associated with the PAD difference, but with shared involvement of the PFC, whose associated genes were enriched in different biological processes. Our results suggested that the risk factors for different brain disorders may converge on neurological mechanisms related to brain aging processes, and the PFC may underlie the important neural processes with respect to the universality of accentuated brain aging. The biological processes of genes related to identified brain patterns in psychiatric disorders, addiction, and dementia may underlie the molecular substrates of accentuated aging phenomenon driven by brain structural abnormalities, which can help us to better understand brain alterations related to aging at a molecular level. From a clinical perspective, genetically-informed and spatially specific PAD patterns could be tested in future studies for their usefulness as biomarkers to guide critical clinical decision-making such as transitions between MCI and AD; differentiation of SZ and BP during the first episode of psychosis and persistence of ADHD into adulthood.

## Supporting information

S1 Checklist STROBE checklist for case-control study. Checklist reproduced from the STROBE Statement (https://www.strobe-statement.org/; von Elm E, Altman DG, Egger M, Pocock SJ, Gøtzsche PC, Vandenbroucke JP, et al. (2007) The Strengthening the Reporting of Observational Studies in Epidemiology (STROBE) Statement: Guidelines for Reporting Observational Studies. PLoS Med 4(10): e296. https://doi.org/10.1371/journal.pmed.0040296) under the Creative Commons Attribution 4.0 International License (CC BY 4.0).
(DOC)

S1 Appendix Supplementary text. **Text A.** Diagnostic criteria and medication status. **Text B.** MRI parameters and preprocessing. **Text C.** Scanner sensitivity analysis. **Text D.** Transcription data processing. **Text E.** Performance verification of the predictive model. **Text F.** Age interaction effects on PAD difference. **Text G.** Dataset, brain atlas and predictive model configurations. **Text H.** Quantitative validation of PAD difference sequence pattern consistency. **Text I.** Bootstrapping analysis of identified brain regions. **Text J.** Site effect.
(DOCX)

S2 Appendix Supplementary tables. **Table A.** Summary of scanner parameters of sMRI for each cohort. HCP, Human Connectome Project; GSP, Brain Genomics Superstruct Project; UKB, UK Biobank; ADHD-200, attention-deficit/hyperactivity disorder-200 project; ABIDE II, Autism Brain Imaging Data Exchange; BSNIP-I, Bipolar and Schizophrenia Network for Intermediate Phenotypes; MDD, major depressive disorder; AUD, alcohol use disorder; TUD, tobacco use disorder; ADNI, Alzheimer’s Disease Neuroimaging Initiative; TR, Repetition Time; TE, Echo Time; FA, Flip Angle. **Table B.** Comparison of predictive performance using full sample vs. sample excluding 1.5T scanners. ADHD-200, attention-deficit/hyperactivity disorder-200 project; ADNI, Alzheimer’s Disease Neuroimaging Initiative; AD, Alzheimer’s disease; MCI, mild cognitive impairment; HC, healthy control; MAE, mean absolute error; *R*^2^, coefficient of determination. **Table C.** Comparison of PAD difference using full sample vs. sample excluding 1.5T scanners. ADHD, attention-deficit/hyperactivity disorder; AD, Alzheimer’s disease; MCI, mild cognitive impairment. The unadjusted Cohen’s *d* reflects group differences without covariates, while the adjusted Cohen’s *d* accounts for age, age^2^, sex, and site. **Table D.** The sample overlap between the corresponding training sets of different diagnostic groups. ADHD, attention-deficit/hyperactivity disorder; ASD, autism spectrum disorder; SZ, schizophrenia; BP, bipolar disorder; MDD, major depressive disorder; AUD, alcohol use disorder; TUD, tobacco use disorder; A&TUD, AUD and TUD; AD, Alzheimer’s disease; MCI, mild cognitive impairment. **Table E.** Model performance in each fold of the training sets without age correction. ADHD, attention-deficit/hyperactivity disorder; ASD, autism spectrum disorder; SZ, schizophrenia; BP, bipolar disorder; MDD, major depressive disorder; AUD, alcohol use disorder; TUD, tobacco use disorder; A&TUD, AUD and TUD; AD, Alzheimer’s disease; MCI, mild cognitive impairment; MAE, mean absolute error; *R*^2^, coefficient of determination, CV, coefficient of variation. **Table F.** Model performance in each fold of the training sets after age correction. ADHD, attention-deficit/hyperactivity disorder; ASD, autism spectrum disorder; SZ, schizophrenia; BP, bipolar disorder; MDD, major depressive disorder; AUD, alcohol use disorder; TUD, tobacco use disorder; A&TUD, AUD and TUD; AD, Alzheimer’s disease; MCI, mild cognitive impairment; MAE, mean absolute error; *R*^2^, coefficient of determination, CV, coefficient of variation. **Table G.** Demographic information of participants in “young” group of each diagnostic group. ADHD, attention-deficit/hyperactivity disorder; ASD, autism spectrum disorder; SZ, schizophrenia; BP, bipolar disorder; MDD, major depressive disorder; AUD, alcohol use disorder; TUD, tobacco use disorder; A&TUD, AUD and TUD; AD, Alzheimer’s disease; MCI, mild cognitive impairment; HC, healthy control; M/F, male/female; sd, standard deviation. **Table H.** Demographic information of participants in “adult” group of each diagnostic group. ADHD, attention-deficit/hyperactivity disorder; ASD, autism spectrum disorder; SZ, schizophrenia; BP, bipolar disorder; MDD, major depressive disorder; AUD, alcohol use disorder; TUD, tobacco use disorder; A&TUD, AUD and TUD; AD, Alzheimer’s disease; MCI, mild cognitive impairment; HC, healthy control; M/F, male/female; sd, standard deviation. **Table I.** Demographic information of participants in “old” group of each diagnostic group. ADHD, attention-deficit/hyperactivity disorder; ASD, autism spectrum disorder; SZ, schizophrenia; BP, bipolar disorder; MDD, major depressive disorder; AUD, alcohol use disorder; TUD, tobacco use disorder; A&TUD, AUD and TUD; AD, Alzheimer’s disease; MCI, mild cognitive impairment; HC, healthy control; M/F, male/female; sd, standard deviation. **Table J.** Age × group interaction effects on PAD. ADHD, attention-deficit/hyperactivity disorder; ASD, autism spectrum disorder; SZ, schizophrenia; BP, bipolar disorder; MDD, major depressive disorder; AUD, alcohol use disorder; TUD, tobacco use disorder; A&TUD, AUD and TUD; AD, Alzheimer’s disease; MCI, mild cognitive impairment; CI, confidence interval. *P* values are derived from *t*-tests based on linear model. **Table K.** Spearman correlations between PAD difference sequence pattern derived from the main analytical pipeline and each alternative validation scenario. HCP, Human Connectome Project; AS, augmented Schaefer; SVR, support vector regression; BPNN, back propagation neural network; RF, random forest. *P* values for Spearman’s correlation are calculated using exact permutation tests. **Table L.** Anatomical information of the identified brain regions associated with the PAD difference in psychiatric disorders. R/L, Right brain/left brain; SZ, schizophrenia; BP, bipolar disorder; MDD, major depressive disorder. **Table M.** Anatomical information of the identified brain regions associated with the PAD difference in addiction. R/L, Right brain/left brain; AUD, alcohol use disorder; TUD, tobacco use disorder. **Table N.** Anatomical information of the identified brain regions associated with the PAD difference in dementia. R/L, Right brain/left brain; AD, Alzheimer’s disease; MCI, mild cognitive impairment. **Table O.** The top 8 most significant GO terms for PLS_T_+ gene sets in each diagnostic group. SZ, schizophrenia; BP, bipolar disorder; MDD, major depressive disorder; AUD, alcohol use disorder; TUD, tobacco use disorder; AD, Alzheimer’s disease; MCI, mild cognitive impairment; GO, gene ontology. *P* values are calculated using the cumulative hypergeometric distribution (Fisher’s exact test) as implemented in Metascape. **Table P.** The top 8 most significant GO terms for PLS_T_− gene sets in each diagnostic group. SZ, schizophrenia; BP, bipolar disorder; MDD, major depressive disorder; AUD, alcohol use disorder; TUD, tobacco use disorder; AD, Alzheimer’s disease; MCI, mild cognitive impairment; GO, gene ontology. *P* values are calculated using the cumulative hypergeometric distribution (Fisher’s exact test) as implemented in Metascape. **Table Q.** Prediction performance with and without site regression in the training set after age correction. ADHD, attention-deficit/hyperactivity disorder; ASD, autism spectrum disorder; SZ, schizophrenia; BP, bipolar disorder; MDD, major depressive disorder; AUD, alcohol use disorder; TUD, tobacco use disorder; A&TUD, AUD and TUD; AD, Alzheimer’s disease; MCI, mild cognitive impairment; MAE, mean absolute error; *R*^2^, coefficient of determination. **Table R.** Prediction performance with and without site regression in the testing sets after age correction. ADHD, attention-deficit/hyperactivity disorder; ASD, autism spectrum disorder; SZ, schizophrenia; BP, bipolar disorder; MDD, major depressive disorder; AUD, alcohol use disorder; TUD, tobacco use disorder; A&TUD, AUD and TUD; AD, Alzheimer’s disease; MCI, mild cognitive impairment; MAE, mean absolute error; *R*^2^, coefficient of determination. **Table S.** Site-wise prediction performance estimated using LOSO validation in training sets after age correction. ADHD, attention-deficit/hyperactivity disorder; ASD, autism spectrum disorder; SZ, schizophrenia; BP, bipolar disorder; MDD, major depressive disorder; AUD, alcohol use disorder; TUD, tobacco use disorder; A&TUD, AUD and TUD; AD, Alzheimer’s disease; MCI, mild cognitive impairment; MAE, mean absolute error; *R*^2^, coefficient of determination, CV, coefficient of variation. **Table T.** Overall prediction performance estimated using 10-fold cross-validation and LOSO validation in the training sets after age correction. ADHD, attention-deficit/hyperactivity disorder; ASD, autism spectrum disorder; SZ, schizophrenia; BP, bipolar disorder; MDD, major depressive disorder; AUD, alcohol use disorder; TUD, tobacco use disorder; A&TUD, AUD and TUD; AD, Alzheimer’s disease; MCI, mild cognitive impairment; MAE, mean absolute error; *R*^2^, coefficient of determination, LOSO, leave-one-site-out. **Table U.** Cross-site differences in PAD of HC from the testing sets. ADHD, attention-deficit/hyperactivity disorder; ASD, autism spectrum disorder; SZ, schizophrenia; BP, bipolar disorder; MDD, major depressive disorder; AUD, alcohol use disorder; TUD, tobacco use disorder; A&TUD, AUD and TUD; AD, Alzheimer’s disease; MCI, mild cognitive impairment; PAD, predicted age difference. The adjusted/unadjusted *p* values are calculated using Analysis of Covariance (ANCOVA) with/without age, age^2^, and sex as covariates.
(DOCX)

S3 AppendixSupplementary figures.**Fig A.** The scatterplot of ages used in each control subgroup. ADHD, attention-deficit/hyperactivity disorder; ASD, autism spectrum disorder; SZ, schizophrenia; BP, bipolar disorder; MDD, major depressive disorder; AUD, alcohol use disorder; TUD, tobacco use disorder; A&TUD, AUD and TUD; AD, Alzheimer’s disease; MCI, mild cognitive impairment. **Fig B.** Samples construction details for training and testing. HCP, Human Connectome Project; GSP, Brain Genomics Superstruct Project; UKB, UK Biobank; ABIDE II, Autism Brain Imaging Data Exchange; BSNIP-I, Bipolar and Schizophrenia Network for Intermediate Phenotypes; ADNI, Alzheimer’s Disease Neuroimaging Initiative; ADHD, attention-deficit/hyperactivity disorder; ASD, autism spectrum disorder; SZ, schizophrenia; BP, bipolar disorder; MDD, major depressive disorder; AUD, alcohol use disorder; TUD, tobacco use disorder; A&TUD, AUD and TUD; AD, Alzheimer’s disease; MCI, mild cognitive impairment; HCs, healthy controls. **Fig C.** Model performance in training sets. Performance metrics of the brain age prediction model in the training sets, shown (a) without and (b) after age correction, including the Pearson correlation (*r*) between predicted brain age and chronological age, mean absolute error (MAE), and coefficient of determination (*R*^2^). The solid line indicates the linear regression fit. Notably, the training sets for different diagnostic groups were independently constructed by matching the age distribution of each test cohort. Consequently, when multiple diagnostic groups share similar or identical age ranges (e.g., MCI and AD; AUD, TUD, and A&TUD), their corresponding training sets may contain substantially overlapping or even identical samples, leading to visually similar scatter plots. The overlap rates between training sets for each pair of diagnostic groups are provided in Table D in S2 Appendix. ADHD, attention-deficit/hyperactivity disorder; ASD, autism spectrum disorder; SZ, schizophrenia; BP, bipolar disorder; MDD, major depressive disorder; AUD, alcohol use disorder; TUD, tobacco use disorder; A&TUD, AUD and TUD; AD, Alzheimer’s disease; MCI, mild cognitive impairment. **Fig D.** Impact of training sample size on the performance of brain age prediction. We used the training-test set corresponding to AD to evaluate the impact of training sample size on brain age prediction model performance (including *r*, MAE and *R*^2^), owing to its large sample size. A total of 34,308 HCs were included as the full training samples, and 361 HCs from ADNI were included as the test samples (excluding 361 ADs as without ground truth, individuals with brain disorder may be not matched brain age and chronological age, Fig 3a). Fifty random subsets of full training set include 100, 500, 2000, 5,000, 10,000 and 20,000 individuals were drawn, and corresponding models cross-validated and applied to testing samples. We also displayed the results from the full training samples (*n* = 34,308). The maxima, upper quartile, median, lower quartile and minima were displayed in the box plots. Panel (a) shows the results for the training sets, and panel (b) shows the results for the test sets. With increasing training sample size, performance of the models increased, with less variation across runs. In boxplots, the lower, middle, and upper bounds of the box represent the first quartile, median, and third quartile, respectively. The lower and upper whiskers represent the minimum and maximum values, respectively. MAE, mean absolute error; *R*^2^, coefficient of determination. **Fig E.** Age distributions and the PAD difference for each diagnostic group based on fMRI. (a) The sample size and age distribution of training (up) and testing (down) sets for each diagnostic group with fMRI data. The gray shading under each diagnostic group represents its age-, sex- and number-matched control group. *P* value represents the group difference of age between diagnostic group and HC calculated by two sample *t* test. The testing samples comprised 341 ADHDs/341 HCs; 484 ASDs/484 HCs; 152 SZs/152 HCs; 143 BPs/143 HCs; 258 MDDs/258 HCs; 145 AUDs/145 HCs; 139 TUDs/139 HCs; 54 A&TUDs/54 HCs; 97 ADs/97HCs; and 385 MCIs/385 HCs. The training samples comprised 9,307 HCs for ADHD, 11,523 HCs for ASD, 2,169 HCs for SZ, 2,182 HCs for BP, 1917 HCs for MDD, AUD and TUD,1810 HCs for A&TUD, 38,456 HCs for AD and MCI, which selected from HCP, GSP and UKB with matched age-range for each diagnostic group. (b) The PAD difference between patient and HC. Cohen’s *d* effect sizes accounting for age, age^2^, sex and site and two-sided *p* values from linear model-based *t*-tests are provided. ADHD, attention-deficit/hyperactivity disorder; ASD, autism spectrum disorder; SZ, schizophrenia; BP, bipolar disorder; MDD, major depressive disorder; AUD, alcohol use disorder; TUD, tobacco use disorder; A&TUD, AUD and TUD; AD, Alzheimer’s disease; MCI, mild cognitive impairment; HC, healthy control. **Fig F.** An example of criteria for dividing each diagnostic group into different age groups. In any given diagnostic group, consisting of a total of *n* samples, we aim to allocate the sample sizes across different age groups as evenly as possible, while ensuring non-overlapping age ranges between groups. Firstly, sort these samples in ascending order of age and mark sample with age ranked $\frac{n}{\text{3}}$. Then, determine the upper (*n*_1_) and lower (*n*_2_) bounds on the ranking of samples with the same age as the marked $\frac{n}{\text{3}}$-th ranked sample. If $\frac{n_{\text{1}}+n_{\text{2}}}{\text{2}}\;$>$\;\frac{n}{\text{3}}$, the samples ranked 1 to (*n*_1_ − 1) were divided into “young” group (this example, if $\frac{n_{\text{1}}+n_{\text{2}}}{\text{2}}\;\leq \frac{n}{\text{3}}$, the samples ranked 1 to *n*_2_ were divided into “young” group). Then, mark sample with age ranked $\frac{n_{\text{1}}+n}{\text{2}}$, and determine the upper (*n*_3_) and lower (*n*_4_) bounds on the ranking of samples with the same age as the marked $\frac{n_{\text{1}}+n}{\text{2}}$-th ranked sample. If $\frac{n_{\text{3}}+n_{\text{4}}}{\text{2}}\;\leq \;\frac{n_{\text{1}}+n}{\text{2}}$, the samples ranked *n*_1_ to *n*_4_ were divided into “adult” group, and the samples ranked (*n*_4_ − 1) to *n*were divided into “old” group (this example, if $\frac{n_{\text{3}}+n_{\text{4}}}{\text{2}}\;>\;\frac{n_{\text{1}}+n}{\text{2}}$, the samples ranked *n*_1_ to (*n*_3_ − 1) were divided into “adult” group and the samples ranked (*n*_3_) to *n* were divided into “old” group). ADHD, attention-deficit/hyperactivity disorder; ASD, autism spectrum disorder; SZ, schizophrenia; BP, bipolar disorder; MDD, major depressive disorder; AUD, alcohol use disorder; TUD, tobacco use disorder; A&TUD, AUD and TUD; AD, Alzheimer’s disease; MCI, mild cognitive impairment; HC, healthy control. **Fig G.** Age-related trajectories of ΔPAD in AD and MCI. ΔPAD was defined as the mean PAD in patients minus the mean PAD in healthy controls within each age window. Smoothed curves illustrate the age-dependent variation of ΔPAD. The operationally defined divergence age corresponds to local extrema or inflection points in the fitted ΔPAD trajectories. For AD, a divergence point was observed at approximately 57 years. For MCI, divergence points were identified at approximately 68 and 80 years. AD, Alzheimer’s disease; MCI, mild cognitive impairment; PAD, predicted age difference. **Fig H.** Predictive performance validation across datasets, atlases and different prediction models. Ten-fold cross-validation was performed in each corresponding training set for the diagnostic group based on multiple (a) training sets, (b) brain atlas scales and (c) prediction models to test the predictive performance (without age correction). Color bar indicates Pearson correlation. HCP, Human Connectome Project; AS, augmented Schaefer; SVR, support vector regression; BPNN, back propagation neural network; RF, random forest. ADHD, attention-deficit/hyperactivity disorder; ASD, autism spectrum disorder; SZ, schizophrenia; BP, bipolar disorder; MDD, major depressive disorder; AUD, alcohol use disorder; TUD, tobacco use disorder; A&TUD, AUD and TUD; AD, Alzheimer’s disease; MCI, mild cognitive impairment. **Fig I.** Repeatability verification of PAD difference T-map under different brain atlas scales using AD as an example. (a) The AD-PAD difference T-map based on augmented Schaefer brain atlas from 116 ROIs to 1,016 ROIs with intervals of 100. Color bar indicates statistical *t*-values of the interaction coefficients, and red/bule brain regions represent more stronger positive/negative associations between the contribution of identified brain features to predicting brain age and PAD in patient groups than in the HC. (b) The correlation matrix between brain atlas scales. All difference T-maps were unified parcellated into 1,016 ROIs based on augmented Schaefer-1016 atlas and calculated the mean voxel value within each ROI. Therefore, we generated a 1,016 × 1 dimensional vector for each difference T-map and then calculated the Pearson correlation between them. Colors bar indicates Pearson correlation *r* value. AD, Alzheimer’s disease; PAD, predicted age difference. **Fig J.** Bootstrapping analysis of the identified brain regions for each disorder. Selection frequency of features identified in the main analysis across 1,000 bootstrap resamples. In each iteration, 80% of the training dataset was randomly sampled without replacement and the same analytical pipeline was applied. Each dot represents one brain region identified in main analysis, and the y-axis indicates the proportion of bootstrap iterations in which the region was re-identified as contributing to the PAD difference. The boxplots illustrate the distribution of re-selection frequencies, where the lower, middle, and upper bounds of the box represent the first quartile, median, and third quartile, respectively. The lower and upper whiskers represent the minimum and maximum values, respectively. SZ, schizophrenia; BP, bipolar disorder; MDD, major depressive disorder; AUD, alcohol use disorder; TUD, tobacco use disorder; AD, Alzheimer’s disease; MCI, mild cognitive impairment; SD, standard deviation. **Fig K.** Variance in the PAD difference explained by all 15 PLS components in diagnostic group. The * represents the variance was significantly greater than random level for this component (1,000 permutation tests). Within the significance components, the first PLS component (PLS1) explained the most variance for SZ (8.1%), BP (5.4%), AUD (7.2%), TUD (7.0%), AD (6.3%) and MCI (5.3%), and the third PLS (PLS3) component for MDD (5.1%). Therefore, the PLS3 for MDD and PLS1 for other brain disorders were selected for further analyses. SZ, schizophrenia; BP, bipolar disorder; MDD, major depressive disorder; AUD, alcohol use disorder; TUD, tobacco use disorder; AD, Alzheimer’s disease; MCI, mild cognitive impairment; PLS, partial least square. **Fig L.** Expression profiles of genes related to PAD difference T-map in diagnostic group. Cortical maps and correlation scatterplots of PAD difference T value and the corresponding regional PLS1 (PLS3 for MDD) scores. The *p* values were obtained from permutation test (1,000 permutations) with corrected spatial autocorrelation. The solid line indicates the linear regression fit. SZ, schizophrenia; BP, bipolar disorder; MDD, major depressive disorder; AUD, alcohol use disorder; TUD, tobacco use disorder; AD, Alzheimer’s disease; MCI, mild cognitive impairment; PLS, partial least square. **Fig M.** The (a) correlation and (b) individual difference between predicted age with and without site regression in the training sets. The solid line indicates the linear regression fit. The *p* values were calculated using paired *t*-tests. In boxplots, the lower, middle, and upper bounds of the box represent the first quartile, median, and third quartile, respectively. The lower and upper whiskers represent the minimum and maximum values, respectively. ADHD, attention-deficit/hyperactivity disorder; ASD, autism spectrum disorder; SZ, schizophrenia; BP, bipolar disorder; MDD, major depressive disorder; AUD, alcohol use disorder; TUD, tobacco use disorder; A&TUD, AUD and TUD; AD, Alzheimer’s disease; MCI, mild cognitive impairment. **Fig N.** The (a) correlation and (b) individual difference between predicted age with and without site regression in the testing sets. The solid line indicates the linear regression fit. The *p* values were calculated using paired *t*-tests. In boxplots, the lower, middle, and upper bounds of the box represent the first quartile, median, and third quartile, respectively. The lower and upper whiskers represent the minimum and maximum values, respectively. ADHD, attention-deficit/hyperactivity disorder; ASD, autism spectrum disorder; SZ, schizophrenia; BP, bipolar disorder; MDD, major depressive disorder; AUD, alcohol use disorder; TUD, tobacco use disorder; A&TUD, AUD and TUD; AD, Alzheimer’s disease; MCI, mild cognitive impairment. **Fig O.** The (a) correlation and (b) individual difference between predicted age estimated using 10-fold cross-validation and leave-one-site-out validation in training sets. The solid line indicates the linear regression fit. The *p* values were calculated using paired *t*-tests. In boxplots, the lower, middle, and upper bounds of the box represent the first quartile, median, and third quartile, respectively. The lower and upper whiskers represent the minimum and maximum values, respectively. ADHD, attention-deficit/hyperactivity disorder; ASD, autism spectrum disorder; SZ, schizophrenia; BP, bipolar disorder; MDD, major depressive disorder; AUD, alcohol use disorder; TUD, tobacco use disorder; A&TUD, AUD and TUD; AD, Alzheimer’s disease; MCI, mild cognitive impairment; LOSO, leave-one-site-out.(DOCX)

## Abbreviations

ABIDE II: Autism Brain Imaging Data Exchange
ACC: anterior cingulate cortex
ADHD: attention-deficit/hyperactivity disorder
ADNI: Alzheimer’s Disease Neuroimaging Initiative
AHBA: Allen Human Brain Atlas
AIBS: Allen Institute for Brain Science
ASD: autism spectrum disorder
AUD: alcohol use disorder
AUDIT: Alcohol Use Disorder Identification Test
BP: bipolar disorder
BSNIP-1: Bipolar-Schizophrenia Network for Intermediate Phenotypes
CC: cingulate cortex
CI: confidence interval
DMN: default mode network
DNA: deoxyribonucleic acid
DSM-IV: Diagnostic and Statistical Manual of Mental Disorders
ENIGMA: Enhancing Neuro-Imaging Genetics through Meta-analysis
fALFF: fraction amplitude of low-frequency fluctuation
FG: fusiform gyrus
fMRI: functional MRI
FTND: Fagerström Test for Nicotine Dependence
GMV: gray matter volume
GSP: Genomics Superstruct Project
HC: healthy control
HCP: Human Connectome Project
HDRS: Hamilton Depression Rating Scale
IOC: inferior occipital cortex
INDI: International Neuroimaging Data-Sharing Initiative
IRB: Institutional Review Board
KKI: Kennedy Krieger Institute
LOSO: leave-one-site-out
MADRS: Montgomery-Asberg Depression Rating Scale
MAE: mean absolute error
MCI: mild cognitive impairment
MDD: major depressive disorder
MMSE: Mini-Mental State Examination
MOC: middle occipital cortex
MTC: middle temporal cortex
NINCDS/ADRDA: Neurological and Communicative Disorders and Stroke and the Alzheimer’s Disease and Related Disorders Association
NYU: New York University Child Study Center
OHSU: Oregon Health and Science University
PAD: predicted age difference
PANSS: Positive and Negative Syndrome Scale
PC: precentral cortex
PFC: prefrontal cortex
PKU: Peking University
PLS: partial least square
ROIs: regions of interest
SAN: salience network
SHAP: Shapley Additive Explanations
sMRI: structural magnetic resonance imaging
SOC: superior occipital cortex
SPC: superior parietal cortex
SZ: schizophrenia
STROBE: Strengthening the Reporting of Observational Studies in Epidemiology
TC: temporal cortex
TUD: tobacco use disorder
UKB: UK Biobank
YMRS: Young Mania Rating Scale
