# Supplementary material for: Brain aging patterns among nine neurological disorders: A case-control study
Source: PLoS Med. 2026 Jul 21;23(7):e1004860. doi: 10.1371/journal.pmed.1004860 (PMC13387544; doi:10.1371/journal.pmed.1004860)
Supplement: S1 Checklist — Checklist reproduced from the STROBE Statement (https://www.strobe-statement.org/; von Elm E, Altman DG, Egger M, Pocock SJ, Gøtzsche PC, Vandenbroucke JP, et al. (2007) The Strengthening the Reporting of Observational Studies in Epidemiology (STROBE) Statement: Guidelines for Reporting Observational Studies. PLoS Med 4(10): e296. https://doi.org/10.1371/journal.pmed.0040296) under the Creative Commons Attribution 4.0 International License (CC BY 4.0). (DOC) [file pmed.1004860.s001.doc]

**STROBE Statement—Checklist of items that should be included in reports of *case-control studies***

|  | | Item No | Recommendation | | Page No | |
| --- | --- | --- | --- | --- | --- | --- |
| **Title and abstract** | | 1 | (*a*) Indicate the study’s design with a commonly used term in the title or the abstract | Title and paragraph ‘Methods and findings’ in the Abstract | | |
|  |  |  | (*b*) Provide in the abstract an informative and balanced summary of what was done and what was found | Paragraph ‘Methods and Findings’ and ‘Conclusions’ in the Abstract | | |
| Introduction | | | | | | |
| Background/rationale | | 2 | Explain the scientific background and rationale for the investigation being reported | Paragraphs 1-2 in the Introduction | | |
| Objectives | | 3 | State specific objectives, including any prespecified hypotheses | Paragraphs 3-4 in the Introduction | | |
| Methods | | | | | | |
| Study design | | 4 | Present key elements of study design early in the paper | Sections ‘Participants’, ‘Brain age prediction with age correction’, ‘PAD group difference comparison’, ‘Interpretable brain patterns of PAD’, ‘Correlation between PAD and symptoms’, and ‘Gene enrichment analyses’ in the Methods | | |
| Setting | | 5 | Describe the setting, locations, and relevant dates, including periods of recruitment, exposure, follow-up, and data collection | Section ‘Participants’ in the Methods, Section ‘Text A’ in S1 Appendix | | |
| Participants | | 6 | (*a*) Give the eligibility criteria, and the sources and methods of case ascertainment and control selection. Give the rationale for the choice of cases and controls | Section ‘Participants’ in the Methods, Section ‘Text A’ in S1 Appendix | | |
|  |  |  | (*b*) For matched studies, give matching criteria and the number of controls per case | Section ‘Participants’ in the Methods, Section ‘Fig B’ in S3 Appendix | | |
| Variables | | 7 | Clearly define all outcomes, exposures, predictors, potential confounders, and effect modifiers. Give diagnostic criteria, if applicable | Sections ‘Brain age prediction with age correction’, ‘PAD group difference comparison’, ‘Interpretable brain patterns of PAD’, ‘Correlation between PAD and symptoms’, and ‘Gene enrichment analyses’ in the Methods | | |
| Data sources/ measurement | | 8* | For each variable of interest, give sources of data and details of methods of assessment (measurement). Describe comparability of assessment methods if there is more than one group | Sections ‘Participants’, and ‘Correlation between PAD and symptoms’ in the Methods, Section ‘Text B’ in S1 Appendix | | |
| Bias | | 9 | Describe any efforts to address potential sources of bias | Sections ‘Brain age prediction with age correction’, ‘PAD group difference comparison’, ‘Interpretable brain patterns of PAD’, and ‘Correlation between PAD and symptoms’ in the Methods | | |
| Study size | | 10 | Explain how the study size was arrived at | Section ‘Participants’ in the Methods | | |
| Quantitative variables | | 11 | Explain how quantitative variables were handled in the analyses. If applicable, describe which groupings were chosen and why | Sections ‘Brain age prediction with age correction’, ‘PAD group difference comparison’, ‘Interpretable brain patterns of PAD’, and ‘Correlation between PAD and symptoms’ in the Methods | | |
| Statistical methods | | 12 | (*a*) Describe all statistical methods, including those used to control for confounding | Sections ‘Brain age prediction with age correction’, ‘PAD group difference comparison’, ‘Interpretable brain patterns of PAD’, ‘Correlation between PAD and symptoms’, and ‘Gene enrichment analyses’ in the Methods | | |
|  |  |  | (*b*) Describe any methods used to examine subgroups and interactions | Sections ‘Brain age prediction with age correction’, ‘PAD group difference comparison’, and ‘Interpretable brain patterns of PAD’ in the Methods | | |
|  |  |  | (*c*) Explain how missing data were addressed | Section ‘Text A’ in S1 Appendix | | |
|  |  |  | (*d*) If applicable, explain how matching of cases and controls was addressed | Sections ‘Participants’ and ‘Brain age prediction with age correction’ in the Methods | | |
|  |  |  | (*e*) Describe any sensitivity analyses | Sections ‘Text C’ in S1 Appendix and ‘Table B-C’ in S2 Appendix. | | |
| Results | | | | | | |
| Participants | | 13* | (a) Report numbers of individuals at each stage of study—eg numbers potentially eligible, examined for eligibility, confirmed eligible, included in the study, completing follow-up, and analysed | Sections ‘Participants’ in the Methods | | |
|  |  |  | (b) Give reasons for non-participation at each stage | NA | | |
|  |  |  | (c) Consider use of a flow diagram | Section ‘Fig 1’ in the Methods | | |
| Descriptive data | | 14* | (a) Give characteristics of study participants (eg demographic, clinical, social) and information on exposures and potential confounders | Section ‘Participants’ in the Methods, Section ‘The performance of brain age prediction’ in the Results | | |
|  |  |  | (b) Indicate number of participants with missing data for each variable of interest | NA | | |
| Outcome data | | 15* | Report numbers in each exposure category, or summary measures of exposure | Section ‘Participants’ in the Methods | | |
| Main results | | 16 | (*a*) Give unadjusted estimates and, if applicable, confounder-adjusted estimates and their precision (eg, 95% confidence interval). Make clear which confounders were adjusted for and why they were included | Sections ‘The performance of brain age prediction’, ‘PAD difference for each diagnostic group’, ‘PAD difference sequence pattern validations across datasets, atlas resolutions and prediction models’, ‘Relationships between PAD and symptoms’ and ‘Enrichment analyses of genes related to PAD difference T-map’ in the Results. | |  |
|  |  |  | (*b*) Report category boundaries when continuous variables were categorized | NA | |  |
|  |  |  | (*c*) If relevant, consider translating estimates of relative risk into absolute risk for a meaningful time period | NA | |  |
| Other analyses | 17 | Report other analyses done—eg analyses of subgroups and interactions, and sensitivity analyses | | Sections ‘PAD difference for each diagnostic group’ in the Results. | |  |
| Discussion | | | | | |  |
| Key results | 18 | Summarise key results with reference to study objectives | | First paragraph in the Discussion | |  |
| Limitations | 19 | Discuss limitations of the study, taking into account sources of potential bias or imprecision. Discuss both direction and magnitude of any potential bias | | Seventh paragraph in the Discussion | |  |
| Interpretation | 20 | Give a cautious overall interpretation of results considering objectives, limitations, multiplicity of analyses, results from similar studies, and other relevant evidence | | Third to Sixth paragraphs in the Discussion | |  |
| Generalisability | 21 | Discuss the generalisability (external validity) of the study results | | Second and third paragraphs in the Discussion | |  |
| Other information | | | | | |  |
| Funding | 22 | Give the source of funding and the role of the funders for the present study and, if applicable, for the original study on which the present article is based | | Financial disclosure sections | |  |

*Give information separately for cases and controls.

**Note:** An Explanation and Elaboration article discusses each checklist item and gives methodological background and published examples of transparent reporting. The STROBE checklist is best used in conjunction with this article (freely available on the Web sites of PLoS Medicine at http://www.plosmedicine.org/, Annals of Internal Medicine at http://www.annals.org/, and Epidemiology at http://www.epidem.com/). Information on the STROBE Initiative is available at http://www.strobe-statement.org.
