# Supplementary material for: Brain aging patterns among nine neurological disorders: A case-control study
Source: PLoS Med. 2026 Jul 21;23(7):e1004860. doi: 10.1371/journal.pmed.1004860 (PMC13387544; doi:10.1371/journal.pmed.1004860)
Supplement: S1 Appendix — Text A. Diagnostic criteria and medication status. Text B. MRI parameters and preprocessing. Text C. Scanner sensitivity analysis. Text D. Transcription data processing. Text E. Performance verification of the predictive model. Text F. Age interaction effects on PAD difference. Text G. Dataset, brain atlas and predictive model configurations. Text H. Quantitative validation of PAD difference sequence pattern consistency. Text I. Bootstrapping analysis of identified brain regions. Text J. Site effect. (DOCX) [file pmed.1004860.s002.docx]

# **Text A.** **Diagnostic criteria and** **medication status**

All diagnoses for both healthy controls (HCs) and patient groups were established by the original cohorts according to standardized clinical protocols. For healthy control participants, inclusion criteria in the original datasets required the absence of any current or past neurological or psychiatric disorders, as assessed by structured clinical interviews and/or medical record review conducted by trained clinicians. For patients with brain disorders, diagnoses were determined by board-certified psychiatrists or neurologists using established diagnostic criteria (e.g., DSM-IV/DSM-5, depending on the cohort). Specifically, the diagnostic procedures for each dataset are described as follows.

- **Human Connectome Project (HCP)**: Healthy participants were required to have no history of prematurity (birth >3 weeks early, birth weight <5 pounds, or neonatal intensive care unit stay ≥2 days) and to be free from major neurological, medical, endocrine, infectious, or autoimmune conditions; cancer treated with chemotherapy or radiation; long-term steroid or immunosuppressive use; or daily prescription treatment for migraines. Additionally, they had no lifetime history of psychiatric or neurodevelopmental disorders requiring specialist treatment for more than 12 months (e.g., attention-deficit/hyperactivity disorder, autism spectrum disorder, mood or psychotic disorders), no significant head injury or related hospitalization, no prior neurological or psychiatric hospitalization, no receipt of special education services, and no contraindications to magnetic resonance imaging (MRI) scanning [[1](#_ENREF_1)].
- **Brain Genomics Superstruct Project (GSP)**: Healthy participants were required to have no current or past Axis I psychiatric or neurological disorders, and no current use of psychotropic medications or acute physical illness [[2](#_ENREF_2)].
- **UK Biobank (UKB)**: Healthy participants were required to have no International Classification of Diseases, 10th Revision (ICD-10) coded neurological or psychiatric disorders, congenital neurological diseases, or self-reported history of a neurological or psychiatric condition (regardless of ICD-10 coding) [[3](#_ENREF_3)].
- **Attention-deficit/hyperactivity disorder-200 project (ADHD-200)**: ADHD diagnoses were established using standardized clinical interviews such as the Diagnostic Interview for Children and Adolescents (DICA-IV), the Kiddie Schedule for Affective Disorders and Schizophrenia, Present and Lifetime Version (KSADS-PL), or the Computerized Diagnostic Interview Schedule IV (C-DIS-IV), supplemented with parent- and teacher-rated ADHD scales, including the Conners’ Parent Rating Scale (CPRS), the DuPaul ADHD Rating Scale (DuPaul), or the ADHD Rating Scale IV (ADHD-RS IV). Inclusion in the ADHD group generally required meeting Diagnostic and Statistical Manual of Mental Disorders, Fourth or Fifth Edition (DSM-IV/DSM-5) criteria for ADHD, along with elevated scores on the relevant rating scales, while HCs were required to have no current or past psychiatric or neurological disorders, normal cognitive function, and low ADHD rating scores. Additional exclusion criteria included a history of neurological illness, chronic medical conditions, or use of psychotropic medications within a washout period prior to scanning. Intelligence was assessed using standardized instruments such as the Wechsler Abbreviated Scale of Intelligence (WASI) or the Wechsler Intelligence Scale for Children (WISC), depending on the site. More detailed diagnostic criteria and recruitment procedures for the ADHD‑200 dataset are publicly available on the ADHD‑200 project website (<https://fcon_1000.projects.nitrc.org/indi/adhd200/>).
- **Autism Brain Imaging Data Exchange (ABIDE II)**: Autism spectrum disorder (ASD) diagnoses were established using standardized clinical procedures, including the Autism Diagnostic Observation Schedule (ADOS/ADOS-2/ADOS-Generic), the Autism Diagnostic Interview-Revised (ADI-R), structured clinical interviews, and clinical consensus by expert clinicians. Inclusion in the ASD group generally required meeting DSM-IV/DSM-5 criteria for ASD, confirmed through rating scales such as the Social Responsiveness Scale (SRS) or the Social Communication Questionnaire (SCQ), and review of medical or developmental records depending on the site. HCs were required to have no current or past psychiatric or neurological disorders, normal cognitive function, and low scores on ASD-related rating scales. Additional exclusion criteria included MRI incompatibility, significant neurological or medical conditions, or intellectual disability below site-specific thresholds. More detailed information about site-specific diagnostic procedures is publicly available on the ABIDE-II project website (<https://fcon_1000.projects.nitrc.org/indi/abide/abide_II.html>).
- **Bipolar and Schizophrenia Network for Intermediate Phenotypes (BSNIP-1)**: The diagnosis of schizophrenia (SZ) and bipolar disorder (BP) was based on the Structured Clinical Interview for DSM-IV Axis I Disorders, Patient Edition. HCs were required to have no personal history of a psychotic disorder or recurrent depression and no known immediate family history of SZ and BP. The extensive clinical information on each participant was reviewed in a best-estimate diagnostic meeting with at least two experienced research clinicians, to establish the consensus diagnosis. Cross-site diagnostic conference calls were carried out monthly; they were chaired by two senior primary investigators (C.A.T., M.S.K.) and attended by the 2–4 trained clinical assessors at each site. At study start, there was a face-to-face training session for all raters, with a requirement for reliability above 0.85. Each month, diagnostic conferences were held with in-depth diagnostic discussions. Each year, rater training was repeated to reestablish reliability [[4](#_ENREF_4)].
- **Major depressive disorder (MDD)**: All patients with MDD were enrolled based on the Structured Clinical Interview for DSM-IV (SCID) for major depression. Exclusion criteria included patients with concurrent neurological illness, mental retardation, cardiovascular disease, bipolar disorder, schizophrenia, anxiety disorder, alcohol or drug abuse and history of loss of consciousness. The control participants were also interviewed by professional psychiatrists using SCID non-patient edition (SCIDI/NP) to ensure that none of them had a current or past history of depression, other major physical or neurological illnesses, or any substance abuse [[5](#_ENREF_5), [6](#_ENREF_6)].
- **Alcohol use disorder (AUD) and tobacco use disorder (TUD)**: The alcohol and tobacco dependence severity in people who drink alcohol and smoke tobacco was assessed with Alcohol Use Disorder Identification (AUDIT) and Fagerström Test for Nicotine Dependence (FTND), respectively. Individuals with AUDIT>7/FTND>7 were categorized as AUD/TUD, according to previous literature [[7](#_ENREF_7), [8](#_ENREF_8)].  The exclusion criteria of AUD/TUD included injury to the brain, brain-related medical problems, or psychiatric disorders. In addition, the use of illicit drugs confirmed or rejected by urinalysis was also excluded. HCs were required to have no history of alcohol consumption or tobacco use, no history of significant brain injury or brain-related medical conditions, and no evidence of psychotic symptoms as determined by diagnostic interviews [[9](#_ENREF_9)].
- **Alzheimer's Disease Neuroimaging Initiative (ADNI)**: Alzheimer's disease (AD) participants met the National Institute of Neurological and Communicative Disorders and Stroke and the Alzheimer's Disease and Related Disorders Association (NINCDS/ADRDA) criteria for probable AD, exhibited abnormal memory performance, Mini-Mental State Examination (MMSE) scores typically between 20 and 26, and Clinical Dementia Rating (CDR) scores of 0.5 or 1.0. Mild cognitive impairment (MCI) participants were required to report subjective memory concerns, show abnormal memory performance on the cognitive scale based on education-adjusted cutoffs, have largely preserved general cognition and daily functioning, MMSE scores typically between 24 and 30, and a CDR score of 0.5 with a memory box score of at least 0.5, without meeting criteria for dementia. HC participants could have subjective memory complaints or none, but demonstrated normal memory performance on the education-adjusted Logical Memory II subscale of the Wechsler Memory Scale-Revised, MMSE scores between 24 and 30, and a CDR score of 0, with no significant impairment in cognition or activities of daily living. Across all groups, participants with significant neurological, psychiatric, or medical conditions that could affect cognition were excluded. Detailed diagnostic standards for ADNI are publicly accessible through the ADNI documentation portal at: <https://adni.loni.usc.edu/help-faqs/adni-documentation/>.

In the present study, we further applied consistent quality control procedures across datasets, including the exclusion of individuals with missing diagnostic information, poor imaging quality, or incomplete demographic data, to ensure the reliability of diagnostic labels used in our analyses.

Moreover, regarding medication or other treatment status for the diagnostic groups, the currently available information is as follows:

- **ADHD**: 137 individuals had never received medication, 73 had a history of or were currently receiving medication, and for 134 individuals, medication status was unknown.
- **ASD**: 134 individuals were receiving medication, primarily including ASD- or comorbidity-related psychotropic agents such as stimulants, antidepressants, antipsychotics, and mood stabilizers, as well as a smaller number of non-psychiatric medications and supplements. Additionally, 303 individuals with ASD were not receiving medication, and medication information was unavailable for 47 individuals.
- **SZ and BP**: Except for 5 individuals with SZ and 2 individuals with BP whose medication status was unknown, all remaining patients had a history of long-term pharmacological treatment. Medication regimens varied across diagnostic groups, with antipsychotic medications commonly prescribed for individuals with SZ and for a subset of those with BP, whereas lithium was frequently used in individuals with BP.
- **MDD**: Regarding medication or other treatment status for the MDD group, detailed information was unavailable because several contributing cohorts did not systematically record treatment histories.
- **AUD and TUD**: Participants were not undergoing treatment specifically targeting alcohol or tobacco use.
- **AD and MCI**: Although information on participants’ use of antidementia medications was available in the ADNI database, these data were not retrieved or analyzed in the present study.

# **Text B. MRI parameters and preprocessing**

For the sMRI data was normalized to MNI space using the unified segmentation method[[10](#_ENREF_10)] in SPM12, resliced to 3 × 3 × 3 mm, and segmented into gray matter (GM), white matter (WM), and cerebral spinal fluid (CSF) using modulated normalization algorithms, resulting outputs as gray matter volume (GMV). Then the GMV were smoothed using a Gaussian kernel with a full width at half maximum (FWHM) = 6 mm[[11](#_ENREF_11)]. Individual outlier detection was further performed using a spatial Pearson correlation with the template image to ensure that all individuals were properly segmented.

For fMRI, standard preprocessing based on statistical parametric mapping (SPM12, http://www.fil.ion.ucl.ac.uk/spm/) under MATLAB 2019 environment included the following: 1) the first five dummy scans have been discarded to retain only scans when the scanner approach to the steady state 2) slice timing correction; 3) realignment;4) normalization to the EPI template with 3 $\times$ 3 $\times$ 3mm^3^ resolution; 5) spatial smoothing using a 6-mm full width half-maximum Gaussian kernel; After that, nuisance covariates (6 head motions + cerebrospinal fluid [CSF] + white matter [WM]) + global signal were regressed out via a general linear model from the voxel time series, and 7) linear drift was removed through temporal bandpass filtering (0.01–0.08 Hz). The filtered time series was transformed into the frequency domain with a fast Fourier transform, and the power spectrum was obtained. Amplitude of low frequency fluctuation (ALFF) was measured by obtaining the square root of the signal across 0.01–0.08 Hz for each voxel. For standardization purposes, the ALFF of each voxel was divided by the global mean of ALFF values for each individual. The fractional amplitude of low-frequency fluctuations (fALFF) was calculated as a ratio of the power of each frequency at the low-frequency range (0.01–0.08 Hz) to that of the entire frequency range (0–0.25 Hz).

# **Text C. Scanner sensitivity analysis**

A small portion of the data were acquired using 1.5T scanners owing to the technological limitations at the time, including 128 AD, 186 MCI, and 232 HC participants from ADNI, as well as 4 HC and 28 ADHD participants from the ADHD-200 dataset. All remaining neuroimaging data were acquired using 3T scanners. To investigate the potential impact of scanner variability, we conducted a sensitivity analysis restricted to the test datasets that included 1.5T acquisitions (i.e., the ADNI and ADHD-200 datasets). In this analysis, we compared the predictive performance (including Pearson correlation-*r*, mean absolute error-MAE, coefficient of determination-R^2^) and the PAD difference between patients and healthy controls (HC, evaluated using Cohen's *d*) derived from the full test samples with those derived after excluding participants scanned with 1.5T scanners. Results showed negligible differences in both predictive performance (**Table B in S2 Appendix**) and PAD difference between patients and HC (**Table C in S2 Appendix**). In summary, we believe that the very small proportion (<2%) of 1.5T scans is unlikely to materially influence the main results of the study.

# **Text D. Transcription data processing**

Brain gene expression profiles were first obtained from the Allen Human Brain Atlas (AHBA) database[[12](#_ENREF_12)]. The AHBA consists of microarray data for 3702 distinct tissue samples collected from the postmortem brains of six human donors (age: 42.50$\pm$13.38 years; male/female: 5/1). The abagen (https://github.com/netneurolab/abagen) toolbox was used to pre-process the data with the following steps. First, microarray probes were reannotated to remove probes that cannot be reliably matched to genes. Following previously published guidelines for probe-to-gene mappings and intensity-based filtering[[13](#_ENREF_13)], the reannotated probes were filtered based on their intensity relative to the background noise level; probes with intensity less than background in ≥50% of samples were discarded. Second, a single probe with the highest differential stability was selected to represent each gene. This procedure retained 15632 probes, each representing a unique gene. Third, tissue samples were assigned to brain regions using their corrected MNI coordinates (https://github.com/chrisfilo/alleninf) by finding the nearest region within a radius of 2 mm. Samples assigned to the same brain region were averaged separately for each donor. Finally, a scaled robust sigmoid (SRS) method was used to normalize expression values for each sample and donor across all genes, and for each gene and donor across all samples. As a result, a gene expression matrix (1016 samples$\times$​15632 genes) was used for subsequent analyses.

# **Text E. Performance verification of the predictive model**

To validate the effectiveness of the brain age prediction model, we evaluated the overall predictive performance by aggregating the predictions from all cross-validation folds and computing the correlation between predicted brain age and chronological age, as well as the mean absolute error (MAE) and coefficient of determination (R²), across the entire healthy training sets. Results showed high correlation between the predicted brain age and chronological age (without age correction: *r*=0.68$\sim$0.88; after age correction: *r*=0.89$\sim$0.93), accompanied by low MAE (without age correction: MAE=1.65$\sim$4.97; after age correction: MAE=1.15$\sim$3.75) and high R^2^ (without age correction: R^2^=0.45$\sim$0.76; after age correction: R^2^=0.76$\sim$0.84, **Fig C in S3 Appendix**), confirming the excellent predictive performance of the model and highlighting the necessity of age-bias correction. Moreover, we also reported the predictive performance for each cross-validation fold and calculated the coefficient of variation (CV) to evaluate the stability of the brain age prediction model. Results (**Table E in S2 Appendix**) showed consistently high performance across folds, with CV values ranged from 0.9% to 9.9% for *r*, 1.1% to 10.6% for MAE, and 1.8% to 12.1% for R² across diagnostic groups without age correction, all below a commonly used empirical threshold of 15% for acceptable stability [[14](#_ENREF_14)], and were further reduced after age correction (0.5% to 2.4% for *r*, 1.1% to 10.5% for MAE, and 0.7% to 6.0% for R², **Table F in S2 Appendix**). Together, these results indicated that the brain age prediction model exhibits stable and reliable generalization ability, providing strong support for interpreting PAD in patient groups.

# **Text F. Age interaction effects on PAD difference**

we estimated a multiple linear regression model with PAD as the dependent variable, and age, group, sex, site, and the interaction term age $\times$group as independent variables to evaluate the age × group interaction effects on the PAD. Results showed (**Table J in S2 Appendix**) that significant age × group interaction effects were observed for AD ($\beta$ = −0.26, 95% CI [−0.32, −0.20]; *p* < 0.001) and MCI ($\beta$ = −0.10, 95% CI [−0.14, −0.05]; *p* < 0.001), indicating that age influences PAD differently in these brain disorders compared with HC. No significant interaction effects were detected for ADHD, ASD, SZ, BP, MDD, AUD, TUD, or A&TUD (all *p* > 0.05). Moreover, to further characterize how the patient (AD and MCI)-HC difference varied across age, we computed ΔPAD (defined as mean PAD in patients minus mean PAD in HCs within each age window) and assessed its age-related trajectory to capture patterns of divergence between groups. Here, we operationally defined the “divergence age” as the point which the ΔPAD-age trajectory changed its direction of variation, corresponding to a local extremum or inflection point in the fitted ΔPAD trajectory. This definition captures the point at which the pattern of PAD difference between patient and HC shifts, reflecting the age at which group differences in brain age trajectories begin to alter their trend. Results (**Fig G in S3 Appendix**) showed that the ΔPAD trajectory in AD exhibited a directional change around 57 years, whereas in MCI, the trajectory demonstrated turning points at approximately 68 and 80 years.

# **Text G. Dataset, brain atlas and predictive model configurations**

For the cross-dataset robustness validation (**Fig 4a**), age-range matched healthy individuals from the HCP dataset were used as the training set for each diagnostic group. The trained model was subsequently applied to predict brain age in each diagnostic group, and PAD values were calculated accordingly. Moreover, all other steps of the analysis pipeline were maintained identical to those used in the main analysis.

The Schaefer Atlas [[15](#_ENREF_15)] provides cortical parcellations at 10 spatial resolutions ranging from 100 to 1000 parcels in steps of 100, allowing flexible control of atlas granularity. For the cross-atlas robustness validation (**Fig 4b**), we selected three representative resolutions (200, 500, and 800 parcels) from the Schaefer Atlas and combined each with the same Melbourne subcortical brain atlas [[16](#_ENREF_16)] (16 subcortical regions of interest (ROIs)) to construct corresponding augmented atlases (augmented Schaefer-216/516/816). Averaged GMV extracted from these 216/516/816 ROIs were used as features for brain age prediction, while all other steps of the analysis pipeline were kept identical to those used in the main analysis.

For the cross-model robustness validation (**Fig 4c**), we implemented three alternative prediction models, including support vector regression (SVR), back-propagation neural network (BPNN), and random forest (RF), using the same input features and training samples as in the main analysis. Specifically, SVR employed a radial basis function (RBF) kernel to capture nonlinear relationships between input features and the target variable, with hyperparameters set as follows: penalty parameter $C=1.0$, kernel width $\gamma=0.01$, and $\varepsilon$-insensitive loss parameter $\varepsilon=0.01$, while all other parameters were set to default. BPNN was implemented with a single hidden layer of 10 neurons using a sigmoid activation function, trained with a learning rate of 0.1 for a maximum of 200 epochs to minimize mean squared error. RF was implemented as an ensemble of 10 regression trees, with bootstrapped sampling for each tree, a minimum of 4 samples per leaf, a maximum tree depth of 5, and mean squared error as the split criterion, all other parameters were kept at their default values. All models used input features (GMV) and target variables (chronological age) normalized to the range [0,1] prior to training and inverse-transformed after prediction to ensure comparable scales across all dimensions. Besides, all other steps of the analysis pipeline remained identical to those used in the main analysis.

# **Text H. Quantitative validation of PAD difference sequence pattern consistency**

The Spearman rank correlation coefficient $\beta$ was used to evaluate the consistency of the PAD difference sequence pattern among diagnostic categories across datasets, brain atlases and prediction models. Specifically, we calculated Spearman rank correlations between the vector of effect sizes (Cohen’s *d*, one value per diagnostic category) derived from the main analytical pipeline with the corresponding vectors derived from each alternative validation scenario. This approach evaluates whether the relative ordering pattern of PAD differences across diagnostic categories are preserved across analytical settings. A high and statistically significant $\beta$ was interpreted as evidence that the relative order of PAD difference among diagnostic categories is robust. Results (**Table K in S2 Appendix**) showed that the PAD difference sequence pattern among diagnostic categories was highly consistent to the main result when using the HCP training set ($\beta$=0.90, *p*=8.80e-04), different atlases resolution ($\beta$=1.00/0.98/0.99 and all *p*<1.00e-16 for AS-216/516/816), and alternative prediction models ($\beta$=0.83/0.91/0.88 and *p*=3.18e-03/3.07e-04/1.98e-03 for SVR/BPNN/RF).

# **Text I. Bootstrapping analysis of identified brain regions**

To further assess the robustness of the identified brain features, we conducted the bootstrapping analysis. Specifically, 1000 bootstrap iterations were performed, in each of which 80% of the entire training dataset was randomly sampled without replacement to form a training subset, and the same analytical pipeline as in the main analysis was applied to identify the brain features contributing to the PAD difference for each disorder. For each feature identified in the main analysis, we then calculated its selection frequency, defined as the number of bootstrap iterations in which the feature was re-identified as contributing to the PAD difference divided by the total number of bootstrap iterations. Higher selection frequency indicates greater stability of the identified feature. Results (**Fig J in S3 Appendix**) showed that the identified brain features exhibited high re-selection frequencies across 1000 resampling conditions (for each disorder, the average re-selection frequency across identified brain regions exceeded 80%), although the degree of stability varied somewhat across diagnostic groups, indicating the overall robustness of the identified features.

# **Text J. Site effect**

To evaluate the potential impact of site effects on model performance, we controlled for site as covariate using a deconfounding approach prior to the prediction analysis. Deconfounding refers to modeling the relationship between brain features (***X***) and covariates (***Z***) using a linear regression model, subsequently removing the part of the signal captured by ***Z*** from ***X*** to eliminate the influence of confounding factors on brain features, as shown in equation (1):

$$\begin{aligned} \boldsymbol{X}_{clean}=\boldsymbol{X}-f\left( \boldsymbol{Z} \right), \#\left( 1 \right) \end{aligned}$$

where ***X*** is the original brain features, $f$ is a predictive model regressing ***X*** on ***Z*** such that $f\left( \boldsymbol{Z} \right)=E(\mathbf{X}|\mathbf{Z})$ and $\boldsymbol{X}_{clean}$ is the regression residual, *i.e.,* the portion of the feature variable whose variance is not explained by the covariates. These residuals are then used in lieu of the original features as the prediction input. This approach helps to minimize the potential influence of site effects on the subsequent prediction analysis. Then, we conducted additional analyses comparing the prediction performance with and without site regression in both the training and testing sets after age correction. Specifically, we trained brain age prediction models using the original features (without site regression) and the deconfounded features (with site regression), respectively. We then compared the prediction performance between the two approaches by evaluating the correlation between predicted brain age and chronological age, as well as the mean absolute error (MAE), and coefficient of determination (R²). In addition, to quantify the consistency between the two strategies, we calculated the Pearson correlation between the predicted brain age obtained from models with and without site regression. Furthermore, we performed paired t-tests to assess whether there were significant differences between the predicted brain age generated by the two models across individuals.

Results showed that the prediction performance after regressing out site as a covariate was comparable to that obtained without site correction in both the training (**Table Q in S2 Appendix**) and testing sets (**Table R in S2 Appendix**).

Moreover, the predicted brain ages derived from the two approaches were highly correlated (*r*=0.90$\sim$0.98 in training sets, *r*=0.91$\sim$0.98 in testing sets), and the paired t-tests revealed no significant differences between them (*p*>0.05 in both training and testing sets, **Fig M-N in S3 Appendix**).

The above results indicate that site is not a major confounding factor for brain age prediction in this study, and that the observed PAD differences are unlikely to be driven by site-related acquisition effects.

Furthermore, we performed leave-one-site-out (LOSO) validation to evaluate the robustness of the brain age prediction model across different sites. In this validation framework, data from one site were iteratively left out as an independent test set, while the model was trained on data from all remaining sites. This procedure was repeated for each site, such that every site served once as the held-out test set. We then evaluated the predictive performance for each site and calculated the coefficient of variation (CV).

Results showed consistently high performance across sites, with CV values ranging from 0.2% to 2.7% for *r*, 1.1% to 11.3% for MAE, and 0.6% to 6.0% for R² across diagnostic groups after age correction (**Table S in S2 Appendix**), all below a commonly used empirical threshold of 15% for acceptable stability [[14](#_ENREF_14)].

We then also compared the prediction results obtained from the ten-fold cross-validation and the LOSO validation in the training sets. Specifically, we evaluated the overall prediction performance of the two validation strategies and calculated the Pearson correlation between the predicted brain age derived from the two approaches. In addition, paired t-tests were performed to assess whether there was significant difference between the predicted brain ages estimated by the ten-fold cross-validation and those obtained from the LOSO validation across individuals.

Results showed that the prediction performance of the LOSO validation was comparable to that obtained from the ten-fold cross-validation (**Table T in S2 Appendix**). In addition, the predicted brain ages derived from the two strategies were highly correlated (*r* = 0.96$\sim$0.98), and paired t-tests revealed no significant differences between them (*p* > 0.05, **Fig O in S3 Appendix**), indicating that the brain age prediction models are robust to site-specific variability.

Moreover, we also directly examined whether residual site-related variance was present at the level of PAD in HC from the testing sets. First, we reported the mean PAD for HC at each site in **Table U in S2 Appendix**, including only sites with at least ten individuals to ensure reliable estimation. We then performed an Analysis of Covariance (ANCOVA) to test whether PAD differed significantly across sites, with site included as the independent variable and age, age², and sex included as covariates. Results (**Table U in S2 Appendix**) showed that, in HC participants across all testing sets, the site effect on PAD did not reach statistical significance (ADHD: *p* = 0.85, ASD: *p* = 0.49, SZ: *p* = 0.09, BP: *p* = 0.31, MDD: *p* = 0.23, AUD: *p* = 0.19, TUD: *p* = 0.75, A&TUD: *p* = 0.98, AD: *p* = 0.29, and MCI: *p* = 0.51). Moreover, when we repeated the analysis without covariates, the site effect also remained non-significant for all datasets. Overall, these findings indicate that, regardless of whether age, age², and sex are controlled for, there is no strong evidence that PAD differs systematically across sites. Therefore, residual site related offsets in predicted age are unlikely to substantially bias the patient-control PAD comparisons reported in our main analyses.

Collectively, these results indicate that the brain age prediction models are robust to site effects and that site is not a major confounding factor, and thus the observed PAD differences reflect biological rather than acquisition-related effects.

**REFERENCES**

1. Somerville LH, Bookheimer SY, Buckner RL, Burgess GC, Curtiss SW, Dapretto M, et al. The Lifespan Human Connectome Project in Development: A large-scale study of brain connectivity development in 5–21 year olds. Neuroimage. 2018;183:456-68.

2. Holmes AJ, Hollinshead MO, O’keefe TM, Petrov VI, Fariello GR, Wald LL, et al. Brain Genomics Superstruct Project initial data release with structural, functional, and behavioral measures. Scientific data. 2015;2(1):1-16.

3. Qi S, Sui J, Pearlson G, Bustillo J, Perrone-Bizzozero NI, Kochunov P, et al. Derivation and utility of schizophrenia polygenic risk associated multimodal MRI frontotemporal network. Nature communications. 2022;13(1):4929.

4. Tamminga CA, Ivleva EI, Keshavan MS, Pearlson GD, Clementz BA, Witte B, et al. Clinical phenotypes of psychosis in the Bipolar-Schizophrenia Network on Intermediate Phenotypes (B-SNIP). American Journal of psychiatry. 2013;170(11):1263-74.

5. Qi S, Yang X, Zhao L, Calhoun VD, Perrone-Bizzozero N, Liu S, et al. MicroRNA132 associated multimodal neuroimaging patterns in unmedicated major depressive disorder. Brain. 2018;141(3):916-26.

6. Zhi D, Calhoun VD, Lv L, Ma X, Ke Q, Fu Z, et al. Aberrant dynamic functional network connectivity and graph properties in major depressive disorder. Frontiers in psychiatry. 2018;9:339.

7. Meneses-Gaya ICd, Zuardi AW, Loureiro SR, Crippa JAdS. Psychometric properties of the Fagerström test for nicotine dependence. Jornal brasileiro de pneumologia. 2009;35:73-82.

8. Saad-Hussein A, Mohammed AM, Hafez SF, El-Tahlawy E, Shaheen W, Helmy MA, et al. Environmental and Social factors influencing in nicotine dependence detected through using Fagerström Test for Nicotine Dependence. Egyptian Journal of Environmental Research EJER. 2017;6:68-76.

9. Qiu L, Liang C, Kochunov P, Hutchison KE, Sui J, Jiang R, et al. Associations of alcohol and tobacco use with psychotic, depressive and developmental disorders revealed via multimodal neuroimaging. Translational Psychiatry. 2024;14(1):326.

10. Ashburner J, Friston KJ. Unified segmentation. neuroimage. 2005;26(3):839-51.

11. Ashburner J. A fast diffeomorphic image registration algorithm. Neuroimage. 2007;38(1):95-113.

12. Hawrylycz MJ, Lein ES, Guillozet-Bongaarts AL, Shen EH, Ng L, Miller JA, et al. An anatomically comprehensive atlas of the adult human brain transcriptome. Nature. 2012;489(7416):391-9.

13. Arnatkevic̆iūtė A, Fulcher BD, Fornito A. A practical guide to linking brain-wide gene expression and neuroimaging data. Neuroimage. 2019;189:353-67.

14. Shechtman O. The coefficient of variation as an index of measurement reliability. Methods of clinical epidemiology: Springer; 2013. p. 39-49.

15. Schaefer A, Kong R, Gordon EM, Laumann TO, Zuo X-N, Holmes AJ, et al. Local-global parcellation of the human cerebral cortex from intrinsic functional connectivity MRI. Cerebral cortex. 2018;28(9):3095-114.

16. Tian Y, Margulies DS, Breakspear M, Zalesky A. Topographic organization of the human subcortex unveiled with functional connectivity gradients. Nature neuroscience. 2020;23(11):1421-32.
