# Supplementary material for: Brain aging patterns among nine neurological disorders: A case-control study
Source: PLoS Med. 2026 Jul 21;23(7):e1004860. doi: 10.1371/journal.pmed.1004860 (PMC13387544; doi:10.1371/journal.pmed.1004860)
Supplement: S2 Appendix — Table A. Summary of scanner parameters of sMRI for each cohort. HCP, Human Connectome Project; GSP, Brain Genomics Superstruct Project; UKB, UK Biobank; ADHD-200, attention-deficit/hyperactivity disorder-200 project; ABIDE II, Autism Brain Imaging Data Exchange; BSNIP-I, Bipolar and Schizophrenia Network for Intermediate Phenotypes; MDD, major depressive disorder; AUD, alcohol use disorder; TUD, tobacco use disorder; ADNI, Alzheimer’s Disease Neuroimaging Initiative; TR, Repetition Time; TE, Echo Time; FA, Flip Angle. Table B. Comparison of predictive performance using full sample vs. sample excluding 1.5T scanners. ADHD-200, attention-deficit/hyperactivity disorder-200 project; ADNI, Alzheimer’s Disease Neuroimaging Initiative; AD, Alzheimer’s disease; MCI, mild cognitive impairment; HC, healthy control; MAE, mean absolute error; R2, coefficient of determination. Table C. Comparison of PAD difference using full sample vs. sample excluding 1.5T scanners. ADHD, attention-deficit/hyperactivity disorder; AD, Alzheimer’s disease; MCI, mild cognitive impairment. The unadjusted Cohen’s d reflects group differences without covariates, while the adjusted Cohen’s d accounts for age, age2, sex, and site. Table D. The sample overlap between the corresponding training sets of different diagnostic groups. ADHD, attention-deficit/hyperactivity disorder; ASD, autism spectrum disorder; SZ, schizophrenia; BP, bipolar disorder; MDD, major depressive disorder; AUD, alcohol use disorder; TUD, tobacco use disorder; A&TUD, AUD and TUD; AD, Alzheimer’s disease; MCI, mild cognitive impairment. Table E. Model performance in each fold of the training sets without age correction. ADHD, attention-deficit/hyperactivity disorder; ASD, autism spectrum disorder; SZ, schizophrenia; BP, bipolar disorder; MDD, major depressive disorder; AUD, alcohol use disorder; TUD, tobacco use disorder; A&TUD, AUD and TUD; AD, Alzheimer’s disease; MCI, mild cognitive impairment; MAE, mean absolute error; R2, coeff [file pmed.1004860.s003.docx]

**Table A.** Summary of scanner parameters of sMRI for each cohort.

| **Cohort** | **Parameters** |
| --- | --- |
| HCP | <https://www.humanconnectome.org/hcp-protocols> |
| GSP | TR=2200ms; TE=1.5/3.4/5.2/7.0ms; FA=7^。^; Slices=144; Vendor: Siemens |
| UKB | <https://www.fmrib.ox.ac.uk/ukbiobank/protocol/> |
| ADHD-200 | <https://fcon_1000.projects.nitrc.org/indi/adhd200/> |
| ABIDE II | <https://fcon_1000.projects.nitrc.org/indi/abide/scan_params/> |
| BSNIP-1 | **Baltimore:** TR=2300ms; TE=2.91ms; FA=9^。^; Slices=160; Vendor: Siemens  **Boston:** TR=6.98ms; TE=2.84ms; FA=8^。^; Slices=166; Vendor: GE  **Chicago:** TR=6.98ms; TE=2.84ms; FA=8^。^; Slices=166; Vendor: GE  **Dallas:** TR=6.6ms; TE=2.8ms; FA=8^。^; Slices=170; Vendor: Philips  **Hartford:** TR=2300ms; TE=2.91ms; FA=9^。^; Slices=160; Vendor: Siemens |
| MDD | TR=8.4ms; TE=3.9ms; FA=7^。^; Slices=188; Vendor: Philips |
| AUD | TR=2530ms; TE= 1.64/3.5/5.36/7.22/9.08ms; FA=7^。^; Vendor: Philips |
| TUD | TR=2530ms; TE= 1.64/3.5/5.36/7.22/9.08ms; FA=7^。^; Vendor: Philips |
| ADNI | <http://adni.loni.usc.edu/methods/mri-tool/mri-analysis/> |

HCP: Human Connectome Project; GSP: Brain Genomics Superstruct Project; UKB: UK Biobank; ADHD-200: attention-deficit/hyperactivity disorder-200 project; ABIDE II: Autism Brain Imaging Data Exchange; BSNIP-I: Bipolar and Schizophrenia Network for Intermediate Phenotypes; MDD: major depressive disorder; AUD: alcohol use disorder; TUD: tobacco use disorder; ADNI: Alzheimer's Disease Neuroimaging Initiative; TR: Repetition Time; TE: Echo Time; FA: Flip Angle.

**Table B.** Comparison of predictive performance using full sample vs. sample excluding 1.5T scanners.

| **Dataset** | **ADHD-200** | | | | | | **ADNI** | | | | | | | | |
| --- | --- | --- | --- | --- | --- | --- | --- | --- | --- | --- | --- | --- | --- | --- | --- |
|  | **ADHD** | | | **HC** | | | **AD** | | | **MCI** | | | **HC** | | |
|  | *r* | MAE | R² | *r* | MAE | R² | *r* | MAE | R² | *r* | MAE | R² | *r* | MAE | R² |
| **Full sample** | 0.75 | 1.56 | 0.57 | 0.78 | 1.37 | 0.60 | 0.88 | 3.36 | 0.71 | 0.89 | 2.81 | 0.70 | 0.91 | 2.59 | 0.78 |
| **Exclude 1.5T scanners** | 0.74 | 1.58 | 0.55 | 0.76 | 1.42 | 0.58 | 0.89 | 3.29 | 0.73 | 0.89 | 2.78 | 0.72 | 0.91 | 2.66 | 0.77 |

ADHD-200: attention-deficit/hyperactivity disorder-200 project; ADNI: Alzheimer's Disease Neuroimaging Initiative; AD: Alzheimer's disease; MCI: mild cognitive impairment; HC: healthy control; MAE: mean absolute error; R²: coefficient of determination.

**Table C.** Comparison of PAD difference using full sample vs. sample excluding 1.5T scanners.

|  | **ADHD** | **AD** | **MCI** |
| --- | --- | --- | --- |
| **Full sample (**adjusted/ unadjusted**)** | *d*=0.01/ *d*=0.04 | *d*=0.97/ *d*=0.88 | *d*=0.45/ *d*=0.45 |
| **Exclude 1.5T scanners (**adjusted/ unadjusted**)** | *d*=0.03/ *d*=0.06 | *d*=0.92/ *d*=0.83 | *d*=0.47/ *d*=0.46 |

ADHD: attention-deficit/hyperactivity disorder; AD: Alzheimer's disease; MCI: mild cognitive impairment. The unadjusted Cohen's *d* reflects group differences without covariates, while the adjusted Cohen's *d* accounts for age, age², sex, and site.

**Table D.** The sample overlap between the corresponding training sets of different diagnostic groups.

| **Diagnostic group** | **ADHD** | **ASD** | **SZ** | **BP** | **MDD** | **AUD** | **TUD** | **A&TUD** | **AD** | **MCI** |
| --- | --- | --- | --- | --- | --- | --- | --- | --- | --- | --- |
| **ADHD** | 100% | 54.1% | 7.8% | 7.7% | 8.3% | 3.1% | 3.1% | 0.3% | 0% | 0% |
| **ASD** | 54.1% | 100% | 26.8% | 26.6% | 28.5% | 22.2% | 22.2% | 17.8% | 0.1% | 0.1% |
| **SZ** | 7.8% | 26.8% | 100% | 99.4% | 92.7% | 88.4% | 88.4% | 83.4% | 0.4% | 0.4% |
| **BP** | 7.7% | 26.6% | 99.4% | 100% | 92.1% | 87.8% | 87.8% | 82.9% | 0.4% | 0.4% |
| **MDD** | 8.3% | 28.5% | 92.7% | 92.1% | 100% | 81.1% | 81.1% | 76.1% | 0.4% | 0.4% |
| **AUD** | 3.1% | 22.2% | 88.4% | 87.8% | 81.1% | 100% | 100% | 94.4% | 0% | 0% |
| **TUD** | 3.1% | 22.2% | 88.4% | 87.8% | 81.1% | 100% | 100% | 94.4% | 0% | 0% |
| **A&TUD** | 0.3% | 17.8% | 83.4% | 82.9% | 76.1% | 94.4% | 94.4% | 100% | 0% | 0% |
| **AD** | 0% | 0.1% | 0.4% | 0.4% | 0.4% | 0% | 0% | 0% | 100% | 100% |
| **MCI** | 0% | 0.1% | 0.4% | 0.4% | 0.4% | 0% | 0% | 0% | 100% | 100% |

ADHD: attention-deficit/hyperactivity disorder; ASD: autism spectrum disorder; SZ: schizophrenia; BP: bipolar disorder; MDD: major depressive disorder; AUD: alcohol use disorder; TUD: tobacco use disorder; A&TUD: AUD and TUD; AD: Alzheimer's disease; MCI: mild cognitive impairment.

**Table E.** Model performance in each fold of the training sets without age correction.

| **Fold** | **ADHD** | | | **ASD** | | | **SZ** | | | **BP** | | | **MDD** | | |
| --- | --- | --- | --- | --- | --- | --- | --- | --- | --- | --- | --- | --- | --- | --- | --- |
|  | *r* | MAE | R² | *r* | MAE | R² | *r* | MAE | R² | *r* | MAE | R² | *r* | MAE | R² |
| **Fold1** | 0.88 | 1.43 | 0.73 | 0.88 | 4.06 | 0.77 | 0.81 | 4.73 | 0.66 | 0.78 | 4.98 | 0.60 | 0.79 | 4.77 | 0.62 |
| **Fold2** | 0.75 | 1.82 | 0.55 | 0.89 | 3.99 | 0.74 | 0.78 | 4.80 | 0.60 | 0.83 | 4.88 | 0.67 | 0.83 | 5.18 | 0.66 |
| **Fold3** | 0.85 | 1.54 | 0.67 | 0.90 | 3.26 | 0.80 | 0.82 | 4.95 | 0.64 | 0.83 | 4.52 | 0.67 | 0.79 | 4.96 | 0.62 |
| **Fold4** | 0.76 | 1.86 | 0.56 | 0.85 | 3.23 | 0.73 | 0.77 | 5.17 | 0.58 | 0.82 | 5.02 | 0.66 | 0.77 | 5.01 | 0.60 |
| **Fold5** | 0.76 | 1.88 | 0.57 | 0.93 | 3.31 | 0.85 | 0.78 | 4.91 | 0.61 | 0.77 | 4.65 | 0.59 | 0.83 | 4.49 | 0.69 |
| **Fold6** | 0.86 | 1.46 | 0.73 | 0.90 | 3.06 | 0.80 | 0.80 | 4.96 | 0.64 | 0.74 | 5.32 | 0.54 | 0.80 | 4.87 | 0.62 |
| **Fold7** | 0.88 | 1.57 | 0.73 | 0.80 | 3.97 | 0.63 | 0.80 | 5.08 | 0.63 | 0.84 | 4.95 | 0.69 | 0.79 | 5.28 | 0.63 |
| **Fold8** | 0.81 | 1.69 | 0.64 | 0.87 | 3.27 | 0.75 | 0.75 | 4.82 | 0.55 | 0.82 | 5.01 | 0.64 | 0.82 | 4.62 | 0.66 |
| **Fold9** | 0.80 | 1.47 | 0.63 | 0.85 | 3.77 | 0.71 | 0.76 | 4.98 | 0.58 | 0.76 | 5.54 | 0.56 | 0.81 | 4.44 | 0.65 |
| **Fold10** | 0.86 | 1.71 | 0.68 | 0.90 | 3.42 | 0.79 | 0.80 | 4.92 | 0.63 | 0.79 | 4.80 | 0.62 | 0.84 | 4.83 | 0.69 |
| **CV(%)** | **6.3** | **10.4** | **10.9** | **4.1** | **10.6** | **8.0** | **3.0** | **2.6** | **5.6** | **4.4** | **6.0** | **8.1** | **2.8** | **5.7** | **4.8** |
| **Fold** | **AUD** | | | **TUD** | | | **A&TUD** | | | **AD** | | | **MCI** | | |
|  | *r* | MAE | R² | *r* | MAE | R² | *r* | MAE | R² | *r* | MAE | R² | *r* | MAE | R² |
| **Fold1** | 0.66 | 4.70 | 0.43 | 0.69 | 4.43 | 0.47 | 0.65 | 4.29 | 0.43 | 0.75 | 3.26 | 0.57 | 0.75 | 3.28 | 0.57 |
| **Fold2** | 0.55 | 4.78 | 0.35 | 0.75 | 4.36 | 0.53 | 0.60 | 4.65 | 0.39 | 0.75 | 3.31 | 0.57 | 0.75 | 3.35 | 0.56 |
| **Fold3** | 0.60 | 4.63 | 0.40 | 0.73 | 4.23 | 0.51 | 0.71 | 4.47 | 0.46 | 0.74 | 3.38 | 0.55 | 0.74 | 3.37 | 0.54 |
| **Fold4** | 0.66 | 4.40 | 0.43 | 0.66 | 5.00 | 0.42 | 0.65 | 4.74 | 0.43 | 0.74 | 3.27 | 0.55 | 0.76 | 3.28 | 0.55 |
| **Fold5** | 0.68 | 4.42 | 0.46 | 0.72 | 4.34 | 0.51 | 0.69 | 4.11 | 0.46 | 0.76 | 3.24 | 0.57 | 0.75 | 3.26 | 0.57 |
| **Fold6** | 0.73 | 4.98 | 0.45 | 0.63 | 4.84 | 0.39 | 0.61 | 4.86 | 0.40 | 0.74 | 3.32 | 0.55 | 0.74 | 3.32 | 0.55 |
| **Fold7** | 0.71 | 3.94 | 0.45 | 0.58 | 4.51 | 0.38 | 0.69 | 4.43 | 0.47 | 0.74 | 3.29 | 0.55 | 0.74 | 3.30 | 0.54 |
| **Fold8** | 0.66 | 4.36 | 0.43 | 0.76 | 4.22 | 0.51 | 0.73 | 4.22 | 0.50 | 0.75 | 3.25 | 0.57 | 0.75 | 3.28 | 0.56 |
| **Fold9** | 0.80 | 4.46 | 0.54 | 0.65 | 4.54 | 0.41 | 0.75 | 3.81 | 0.52 | 0.76 | 3.27 | 0.57 | 0.74 | 3.28 | 0.57 |
| **Fold10** | 0.66 | 4.31 | 0.48 | 0.69 | 4.11 | 0.47 | 0.78 | 4.21 | 0.56 | 0.75 | 3.27 | 0.56 | 0.75 | 3.29 | 0.56 |
| **CV(%)** | **9.9** | **6.4** | **11.2** | **8.3** | **6.3** | **12.1** | **8.6** | **7.2** | **11.5** | **0.9** | **1.3** | **1.8** | **0.9** | **1.1** | **2.1** |

ADHD: attention-deficit/hyperactivity disorder; ASD: autism spectrum disorder; SZ: schizophrenia; BP: bipolar disorder; MDD: major depressive disorder; AUD: alcohol use disorder; TUD: tobacco use disorder; A&TUD: AUD and TUD; AD: Alzheimer's disease; MCI: mild cognitive impairment; MAE: mean absolute error; R²: coefficient of determination, CV: coefficient of variation.

**Table F.** Model performance in each fold of the training sets after age correction.

| **Fold** | **ADHD** | | | **ASD** | | | **SZ** | | | **BP** | | | **MDD** | | |
| --- | --- | --- | --- | --- | --- | --- | --- | --- | --- | --- | --- | --- | --- | --- | --- |
|  | *r* | MAE | R² | *r* | MAE | R² | *r* | MAE | R² | *r* | MAE | R² | *r* | MAE | R² |
| **Fold1** | 0.95 | 1.05 | 0.86 | 0.93 | 3.55 | 0.85 | 0.92 | 3.58 | 0.81 | 0.90 | 4.08 | 0.75 | 0.90 | 3.59 | 0.80 |
| **Fold2** | 0.89 | 1.35 | 0.75 | 0.93 | 2.95 | 0.75 | 0.91 | 3.41 | 0.78 | 0.93 | 3.43 | 0.85 | 0.93 | 3.34 | 0.86 |
| **Fold3** | 0.94 | 0.98 | 0.86 | 0.94 | 2.88 | 0.86 | 0.93 | 3.32 | 0.85 | 0.93 | 3.75 | 0.79 | 0.91 | 3.92 | 0.78 |
| **Fold4** | 0.91 | 1.15 | 0.82 | 0.91 | 2.99 | 0.79 | 0.91 | 3.54 | 0.80 | 0.92 | 3.75 | 0.78 | 0.90 | 3.64 | 0.80 |
| **Fold5** | 0.90 | 1.29 | 0.78 | 0.96 | 2.83 | 0.89 | 0.91 | 3.59 | 0.80 | 0.90 | 3.68 | 0.76 | 0.93 | 3.48 | 0.82 |
| **Fold6** | 0.94 | 1.15 | 0.83 | 0.94 | 3.08 | 0.85 | 0.92 | 3.77 | 0.81 | 0.88 | 4.11 | 0.74 | 0.91 | 3.96 | 0.78 |
| **Fold7** | 0.95 | 1.06 | 0.88 | 0.89 | 3.31 | 0.79 | 0.92 | 3.81 | 0.79 | 0.93 | 3.68 | 0.84 | 0.91 | 3.92 | 0.80 |
| **Fold8** | 0.93 | 1.19 | 0.84 | 0.93 | 2.95 | 0.84 | 0.89 | 3.67 | 0.74 | 0.93 | 3.50 | 0.85 | 0.92 | 3.73 | 0.79 |
| **Fold9** | 0.91 | 1.25 | 0.75 | 0.92 | 3.73 | 0.83 | 0.90 | 3.63 | 0.77 | 0.90 | 3.84 | 0.80 | 0.91 | 3.78 | 0.77 |
| **Fold10** | 0.95 | 1.04 | 0.88 | 0.94 | 2.97 | 0.85 | 0.92 | 3.67 | 0.82 | 0.91 | 3.69 | 0.77 | 0.93 | 3.50 | 0.83 |
| **CV(%)** | **2.4** | **10.5** | **6.0** | **2.1** | **9.7** | **5.0** | **1.3** | **4.2** | **3.7** | **1.9** | **5.8** | **5.2** | **1.3** | **5.7** | **3.4** |
| **Fold** | **AUD** | | | **TUD** | | | **A&TUD** | | | **AD** | | | **MCI** | | |
|  | *r* | MAE | R² | *r* | MAE | R² | *r* | MAE | R² | *r* | MAE | R² | *r* | MAE | R² |
| **Fold1** | 0.91 | 2.73 | 0.78 | 0.91 | 2.76 | 0.79 | 0.90 | 2.60 | 0.76 | 0.90 | 2.41 | 0.76 | 0.90 | 2.39 | 0.76 |
| **Fold2** | 0.88 | 2.98 | 0.74 | 0.94 | 2.37 | 0.85 | 0.88 | 3.10 | 0.74 | 0.90 | 2.37 | 0.77 | 0.90 | 2.40 | 0.77 |
| **Fold3** | 0.89 | 3.02 | 0.75 | 0.93 | 2.41 | 0.84 | 0.92 | 2.80 | 0.80 | 0.89 | 2.43 | 0.76 | 0.89 | 2.44 | 0.76 |
| **Fold4** | 0.90 | 2.82 | 0.77 | 0.92 | 2.88 | 0.83 | 0.90 | 2.87 | 0.77 | 0.89 | 2.41 | 0.76 | 0.90 | 2.43 | 0.75 |
| **Fold5** | 0.91 | 2.67 | 0.79 | 0.92 | 2.69 | 0.80 | 0.91 | 2.69 | 0.76 | 0.90 | 2.40 | 0.76 | 0.90 | 2.38 | 0.76 |
| **Fold6** | 0.93 | 2.80 | 0.84 | 0.90 | 2.86 | 0.78 | 0.90 | 2.75 | 0.79 | 0.89 | 2.47 | 0.75 | 0.89 | 2.47 | 0.75 |
| **Fold7** | 0.91 | 2.77 | 0.76 | 0.88 | 2.76 | 0.75 | 0.92 | 2.56 | 0.81 | 0.89 | 2.45 | 0.75 | 0.89 | 2.48 | 0.74 |
| **Fold8** | 0.90 | 2.76 | 0.78 | 0.93 | 2.98 | 0.78 | 0.93 | 2.52 | 0.83 | 0.90 | 2.42 | 0.76 | 0.90 | 2.43 | 0.76 |
| **Fold9** | 0.95 | 2.50 | 0.88 | 0.90 | 2.82 | 0.76 | 0.93 | 2.48 | 0.80 | 0.90 | 2.44 | 0.76 | 0.90 | 2.44 | 0.76 |
| **Fold10** | 0.91 | 2.77 | 0.78 | 0.92 | 2.66 | 0.81 | 0.94 | 2.59 | 0.82 | 0.90 | 2.41 | 0.76 | 0.90 | 2.42 | 0.76 |
| **CV(%)** | **2.2** | **5.3** | **5.4** | **1.9** | **7.2** | **4.2** | **2.0** | **7.0** | **3.7** | **0.6** | **1.1** | **0.7** | **0.5** | **1.3** | **1.1** |

ADHD: attention-deficit/hyperactivity disorder; ASD: autism spectrum disorder; SZ: schizophrenia; BP: bipolar disorder; MDD: major depressive disorder; AUD: alcohol use disorder; TUD: tobacco use disorder; A&TUD: AUD and TUD; AD: Alzheimer's disease; MCI: mild cognitive impairment; MAE: mean absolute error; R²: coefficient of determination, CV: coefficient of variation.

**Table G.** Demographic information of individuals in “young” group of each diagnostic group.

| **Testing set** | **Number** | **Age rang (year)** | **Age (mean**$\pm$**sd)** | **Gender (M/F)** |
| --- | --- | --- | --- | --- |
| **ADHD/HC** | 131/118 | <10 | 8.80$\pm$0.72 | 164/85 |
| **ASD/HC** | 156/171 | <11 | 8.45$\pm$1.16 | 250/77 |
| **SZ/HC** | 51/46 | <27 | 22.86$\pm$3.02 | 57/40 |
| **BP/HC** | 46/43 | <27 | 22.48$\pm$3.06 | 34/55 |
| **MDD/HC** | 88/99 | <26 | 22.11$\pm$2.35 | 81/106 |
| **AUD/HC** | 47/50 | <25 | 22.29$\pm$1.61 | 52/45 |
| **TUD/HC** | 46/47 | <27 | 23.61$\pm$1.92 | 56/37 |
| **A&TUD/HC** | 19/14 | <27 | 24.00$\pm$1.71 | 21/12 |
| **AD/HC** | 114/120 | <73 | 67.51$\pm$4.24 | 115/119 |
| **MCI/HC** | 209/208 | <71 | 65.55$\pm$3.82 | 281/136 |

ADHD: attention-deficit/hyperactivity disorder; ASD: autism spectrum disorder; SZ: schizophrenia; BP: bipolar disorder; MDD: major depressive disorder; AUD: alcohol use disorder; TUD: tobacco use disorder; A&TUD: AUD and TUD; AD: Alzheimer's disease; MCI: mild cognitive impairment; HC: healthy control; M/F: male/female; sd: standard deviation.

**Table H.** Demographic information of individuals in “adult” group of each diagnostic group.

| **Testing set** | **Number** | **Age rang (year)** | **Age (mean**$\pm$**sd)** | **Gender (M/F)** |
| --- | --- | --- | --- | --- |
| **ADHD/HC** | 82/107 | 10-12 | 10.99$\pm$0.59 | 150/39 |
| **ASD/HC** | 147/176 | 11-14 | 11.72$\pm$1.04 | 269/54 |
| **SZ/HC** | 44/61 | 27-39 | 32.00$\pm$4.14 | 60/45 |
| **BP/HC** | 45/51 | 27-41 | 33.47$\pm$4.56 | 36/60 |
| **MDD/HC** | 88/80 | 26-37 | 30.90$\pm$3.61 | 67/101 |
| **AUD/HC** | 58/49 | 25-31 | 27.65$\pm$2.08 | 71/36 |
| **TUD/HC** | 45/53 | 27-35 | 30.93$\pm$2.69 | 72/26 |
| **A&TUD/HC** | 15/21 | 27-34 | 30.25$\pm$2.43 | 26/10 |
| **AD/HC** | 120/129 | 73-79 | 76.16$\pm$1.97 | 130/119 |
| **MCI/HC** | 224/236 | 71-77 | 73.71$\pm$1.98 | 239/221 |

ADHD: attention-deficit/hyperactivity disorder; ASD: autism spectrum disorder; SZ: schizophrenia; BP: bipolar disorder; MDD: major depressive disorder; AUD: alcohol use disorder; TUD: tobacco use disorder; A&TUD: AUD and TUD; AD: Alzheimer's disease; MCI: mild cognitive impairment; HC: healthy control; M/F: male/female; sd: standard deviation.

**Table I.** Demographic information of individuals in “old” group of each diagnostic group.

| **Testing set** | **Number** | **Age rang (year)** | **Age (mean**$\pm$**sd)** | **Gender (M/F)** |
| --- | --- | --- | --- | --- |
| **ADHD/HC** | 131/119 | >12 | 14.51$\pm$2.05 | 225/25 |
| **ASD/HC** | 181/137 | >14 | 23.65$\pm$10.20 | 290/28 |
| **SZ/HC** | 57/45 | >39 | 49.46$\pm$5.29 | 73/29 |
| **BP/HC** | 52/49 | >41 | 50.68$\pm$5.74 | 25/76 |
| **MDD/HC** | 82/79 | >37 | 45.96$\pm$5.66 | 47/114 |
| **AUD/HC** | 50/56 | >31 | 40.40$\pm$6.70 | 93/13 |
| **TUD/HC** | 53/44 | >35 | 44.41$\pm$5.83 | 64/33 |
| **A&TUD/HC** | 20/19 | >34 | 41.67$\pm$6.00 | 33/6 |
| **AD/HC** | 127/112 | >79 | 83.86$\pm3$.09 | 141/98 |
| **MCI/HC** | 224/213 | >77 | 82.24$\pm3$.45 | 202/235 |

ADHD: attention-deficit/hyperactivity disorder; ASD: autism spectrum disorder; SZ: schizophrenia; BP: bipolar disorder; MDD: major depressive disorder; AUD: alcohol use disorder; TUD: tobacco use disorder; A&TUD: AUD and TUD; AD: Alzheimer's disease; MCI: mild cognitive impairment; HC: healthy control; M/F: male/female; sd: standard deviation.

**Table J.** Age $\times$group interaction effects on PAD.

| **Indicator** | **ADHD** | **ASD** | **SZ** | **BP** | **MDD** |
| --- | --- | --- | --- | --- | --- |
| **Interaction coef** | $\beta$=-0.07 | $\beta$=-0.04 | $\beta$=0.01 | $\beta$=0.02 | $\beta$=-0.05 |
| **95% CI** | [-0.14, 0.01] | [-0.09, 0.01] | [-0.12, 0.13] | [-0.10, 0.14] | [-0.15, 0.05] |
| ***P*-value** | *p*=0.06 | *p*=0.13 | *p*=0.93 | *p*=0.79 | *p*=0.29 |
| **Indicator** | **AUD** | **TUD** | **A&TUD** | **AD** | **MCI** |
| **Interaction coef** | $\beta$=-0.04 | $\beta$=-3.00e-3 | $\beta$=0.05 | $\beta$=-0.26 | $\beta$=-0.10 |
| **95% CI** | [-0.15, 0.08] | [-0.12, 0.11] | [-0.15, 0.24] | [-0.32, -0.20] | [-0.14, -0.05] |
| ***P*-value** | *p*=0.54 | *p*=0.96 | *p*=0.63 | *p*<0.001 | *p*<0.001 |

ADHD: attention-deficit/hyperactivity disorder; ASD: autism spectrum disorder; SZ: schizophrenia; BP: bipolar disorder; MDD: major depressive disorder; AUD: alcohol use disorder; TUD: tobacco use disorder; A&TUD: AUD and TUD; AD: Alzheimer's disease; MCI: mild cognitive impairment; CI: confidence interval. *P* values are derived from *t*-tests based on linear model.

**Table K.** Spearman correlations between PAD difference sequence pattern derived from the main analytical pipeline and each alternative validation scenario.

|  | **Training sets** | **Atlas resolution** | | | **Prediction models** | | |
| --- | --- | --- | --- | --- | --- | --- | --- |
|  | **HCP** | **AS-216** | **AS-516** | **AS-816** | **SVR** | **BPNN** | **RF** |
| **Spearman correlation** | 0.90 | 1.00 | 0.98 | 0.99 | 0.83 | 0.91 | 0.88 |
| ***p*-value** | 8.80e-04 | <e-16 | <e-16 | <e-16 | 3.18e-03 | 3.07e-04 | 1.98e-03 |

HCP: Human Connectome Project; AS: augmented Schaefer; SVR: support vector regression; BPNN: back propagation neural network; RF: random forest. *P* values for Spearman's correlation are calculated using exact permutation tests.

**Table L.** Anatomical information of the identified brain regions associated with the PAD difference in psychiatric disorders.

|  | **Brodmann Area** | **volume (cc)** | **random effects: Max Value (x, y, z) R/L** |
| --- | --- | --- | --- |
| **SZ** |  |  |  |
| Prefrontal cortex | 6, 8, 9, 10, 11, 13, 25, 47 | 12.7/4.5 | 4.9 (-36, 61, 2)/3.5 (33, 38, -7) |
| Superior/middle/inferior temporal cortex | 20, 21, 22, 37, 38, 39 | 5.9/3.0 | 6.0 (-53, -30, -11)/4.1 (53, -69, 28) |
| Superior/middle occipital cortex | 18, 19 | 0.9/0.1 | 3.8 (-33, -90, 7)/4.1 (42, -77, 31) |
| Thalamus |  | 4.9/0.0 | 3.4 (-9, -11, 17)/NaN (0, 0, 0) |
| Insula | 13 | 1.4/0.0 | 4.0 (-39, 0, 8)/NaN (0, 0, 0) |
| **BP** |  |  |  |
| Prefrontal cortex | 10 | 1.4/0.0 | 4.4 (-27, 59, 22)/NaN (0, 0, 0) |
| Middle/inferior temporal cortex | 19, 20, 37, 39 | 1.0/0.6 | 5.3 (-48, -60, 17)/3.8 (53, -44, -8) |
| Lingual gyrus | 18, 19 | 0.5/0.0 | 4.1 (-15, -52, 5)/NaN (0, 0, 0) |
| **MDD** |  |  |  |
| Prefrontal cortex | 8, 9, 10, 13 | 1.1/3.8 | 4.5 (-53, 16, 27)/4.0 (36, 61, 5) |
| Middle/inferior temporal cortex | 20, 21, 37 | 0.0/3.8 | NaN/5.8 (62, -9, -12) |
| Middle occipital cortex | 19, 37 | 0.0/0.8 | NaN/5.9 (30, -83, 21) |

R/L: Right brain/left brain; SZ: schizophrenia; BP: bipolar disorder; MDD: major depressive disorder.

**Table M.** Anatomical information of the identified brain regions associated with the PAD difference in addiction.

|  | **Brodmann Area** | **volume (cc)** | **random effects: Max Value (x, y, z) R/L** |
| --- | --- | --- | --- |
| **AUD** |  |  |  |
| Prefrontal cortex | 6, 8, 9, 10, 11, 13, 25, 32, 44, 45, 46, 47 | 41.0/42.6 | 6.1 (-45, 50, 3)/8.5 (39, 48, 28) |
| Precentral cortex | 4, 6, 9, 43, 44 | 8.9/8.2 | 5.2 (-42, 25, 37)/6.7 (59, -4, 42) |
| Cingulate cortex | 23, 24, 31, 32 | 5.7/6.3 | 4.9 (-6, 10, 27)/4.7 (6, -45, 33) |
| Insula | 13, 41 | 2.4/2.4 | 6.8 (-45, -9, 3)/4.4 (33, 15, 8) |
| Putamen |  | 0.0/3.8 | NaN (0, 0, 0)/6.4 (24, 3, 11) |
| Thalamus |  | 2.1/7.6 | 2.8 (-12, -20, 9)/5.7 (21, -20, 9) |
| Superior/middle/inferior temporal cortex | 19, 20, 21, 22, 36, 37, 38, 39, 41, 42 | 20.1/9.4 | 6.8 (-45, -9, 3)/6.0 (53, -64, 1) |
| Middle/inferior occipital cortex | 18, 19 | 1.5/2.6 | 3.1 (-42, -78, 12)/6.0 (50, -73, 1) |
| Lingual gyrus | 17, 18, 19 | 1.6/1.2 | 3.0 (-3, -81, 4)/3.5 (12, -49, 2) |
| Superior parietal cortex | 7 | 0.6/1.0 | 4.2 (-33, -49, 61)/5.4 (33, -47, 52) |
| Fusiform gyrus | 18, 19, 20, 36, 37 | 0.8/2.9 | 4.0 (-48, -16, -24)/3.8 (36, -50, -8) |
| **TUD** |  |  |  |
| Prefrontal cortex | 6, 8, 9, 10, 11, 13, 25, 32, 44, 45, 46, 47 | 42.4/44.1 | 7.3 (-53, 16, 27)/ 8.7 (18, 55, -13) |
| Precentral cortex | 6, 9, 44 | 2.9/4.5 | 7.3 (-48, 9, 11)/5.8 (56, -4, 42) |
| Cingulate cortex | 9, 23, 24, 31, 32 | 4.4/5.2 | 6.2 (-18, 55, -5)/8.7 (12, 63, -16) |
| insula | 13, 40, 41, 47 | 3.8/2.8 | 7.3 (-45, 9, 13)/4.8 (33, 15, 8) |
| Putamen |  | 0.0/3.8 | NaN (0, 0, 0)/5.6 (24, 3, 11) |
| Thalamus |  | 4.9/7.2 | 4.5 (-9, -11, 17)/5.4 (21, -20, 9) |
| Superior/middle/inferior temporal cortex | 13, 19, 20, 21, 22, 37, 38, 39, 41, 42 | 12.7/14.6 | 6.9 (-53, 3, 5)/5.0 (33, 14, -21) |

R/L: Right brain/left brain; AUD: alcohol use disorder; TUD: tobacco use disorder.

**Table N.** Anatomical information of the identified brain regions associated with the PAD difference in dementia.

|  | **Brodmann Area** | **volume (cc)** | **random effects: Max Value (x, y, z) R/L** |
| --- | --- | --- | --- |
| **AD** |  |  |  |
| Prefrontal cortex | 6, 8, 9, 10, 11, 13, 25, 44, 45, 46, 47 | 43.8/26.0 | 4.4 (-56, 2, 39)/4.6 (50, 44, 6) |
| Superior/middle occipital cortex | 18, 19, 37 | 6.0/1.7 | 3.9 (-39, -73, 6)/4.4 (15, -90, 16) |
| Fusiform gyrus | 18, 19, 20, 36, 37 | 5.6/2.2 | 5.0 (-33, -59, -7)/4.9 (27, -79, -11) |
| Para-hippocampus | 19, 27, 28, 30, 34, 35, 36, 37 | 5.1/5.3 | 4.3 (-27, -44, -10)/4.1 (12, -44, 0) |
| Middle/inferior temporal cortex | 19, 20, 21, 22, 37, 38, 39 | 4.6/8.2 | 3.7 (-33, -38, -3)/3.9 (39, 7, -31) |
| Precentral cortex | 4, 6, 9, 43, 44 | 14.9/8.1 | 5.3 (-50, -10, 42)/4.6 (30, -29, 51) |
| Anterior cingulate cortex | 24, 25, 32 | 3.4/1.4 | 3.8 (-6, 47, 9)/2.8 (3, 49, -2) |
| Amygdala |  | 5.1/5.3 | 4.3 (-27, -44, -10)/4.1 (12, -44, 0) |
| Caudate |  | 2.5/0.0 | 3.1 (-12, 7, 19)/NaN (0, 0, 0) |
| Insula | 13, 41, 47 | 9.1/4.3 | 4.3 (-39, -8, 11)/4.3 (33, 24, 10) |
| Thalamus |  | 2.1/0.0 | 2.7 (-12, -20, 9)/NaN (0, 0, 0) |
| **MCI** |  |  |  |
| Prefrontal cortex | 10, 45, 46 | 0.0/1.6 | NaN (0, 0, 0)/7.5 (53, 38, 12) |
| Middle occipital cortex | 18, 19 | 1.9/0.1 | 3.8 (-39, -73, 6)/ 4.0 (27, -81, 21) |
| Fusiform gyrus | 37 | 1.3/0.0 | 4.0 (-33, -50, -8)/NaN (0, 0, 0) |

R/L: Right brain/left brain; AD: Alzheimer's disease; MCI: mild cognitive impairment.

**Table O.** The top 8 most significant GO terms for $\mathrm{PLS}_{T}$+ gene sets in each diagnostic group.

| **GO term** | **Description** | | **Log10 (*p*-value)** | **Log10(*q* value)** |
| --- | --- | --- | --- | --- |
| **SZ** | |  |  |  |
| GO:0002181 | | cytoplasmic translation | -32.02 | -27.85 |
| GO:0006412 | | translation | -27.69 | -23.81 |
| GO:0022613 | | ribonucleoprotein complex biogenesis | -16.06 | -12.66 |
| GO:0007005 | | mitochondrion organization | -13.97 | -10.64 |
| GO:0055086 | | nucleobase-containing small molecule metabolic process | -12.77 | -9.49 |
| GO:0072521 | | purine-containing compound metabolic process | -12.28 | -9.14 |
| GO:0070201 | | regulation of establishment of protein localization | -11.26 | -8.23 |
| GO:0043632 | | modification-dependent macromolecule catabolic process | -11.02 | -8.02 |
| **MDD** | |  |  |  |
| GO:0002181 | | cytoplasmic translation | -37.84 | -33.66 |
| GO:0006412 | | translation | -28.44 | -24.73 |
| GO:0042254 | | ribosome biogenesis | -12.55 | -9.15 |
| GO:0048667 | | cell morphogenesis involved in neuron differentiation | -9.59 | -6.32 |
| GO:0022613 | | ribonucleoprotein complex biogenesis | -8.79 | -5.61 |
| GO:0002503 | | peptide antigen assembly with MHC class II protein complex | -8.75 | -5.61 |
| GO:0002396 | | MHC protein complex assembly | -7.80 | -4.87 |
| GO:0022409 | | positive regulation of cell-cell adhesion | -7.70 | -4.80 |
| **AUD** | |  |  |  |
| GO:0042063 | | gliogenesis | -20.08 | -15.90 |
| GO:0010001 | | glial cell differentiation | -16.79 | -12.91 |
| GO:0044282 | | small molecule catabolic process | -16.19 | -12.49 |
| GO:0046395 | | carboxylic acid catabolic process | -14.80 | -11.47 |
| GO:0002274 | | myeloid leukocyte activation | -14.95 | -11.47 |
| GO:0044283 | | small molecule biosynthetic process | -14.88 | -11.47 |
| GO:0001775 | | cell activation | -14.72 | -11.45 |
| GO:0032787 | | monocarboxylic acid metabolic process | -13.74 | -10.51 |
| **TUD** | |  |  |  |
| GO:0016126 | | sterol biosynthetic process | -12.85 | -8.80 |
| GO:0044283 | | small molecule biosynthetic process | -12.67 | -8.80 |
| GO:1901615 | | organic hydroxy compound metabolic process | -11.97 | -8.39 |
| GO:0008202 | | steroid metabolic process | -11.51 | -8.18 |
| GO:0044282 | | small molecule catabolic process | -10.88 | -7.60 |
| GO:0046395 | | carboxylic acid catabolic process | -10.24 | -7.10 |
| GO:0008610 | | lipid biosynthetic process | -9.44 | -6.38 |
| GO:0009636 | | response to toxic substance | -9.41 | -6.38 |
| **AD** | |  |  |  |
| GO:0072657 | | protein localization to membrane | -7.49 | -3.31 |
| GO:1903530 | | regulation of secretion by cell | -6.86 | -2.99 |
| GO:0051668 | | localization within membrane | -6.35 | -2.65 |
| GO:0140352 | | export from cell | -6.12 | -2.6 |
| GO:0007007 | | inner mitochondrial membrane organization | -5.70 | -2.30 |
| GO:0032940 | | secretion by cell | -5.62 | -2.29 |
| GO:0046903 | | secretion | -5.45 | -2.22 |
| GO:0099536 | | synaptic signaling | -5.02 | -1.88 |
| **MCI** | |  |  |  |
| GO:0007005 | | mitochondrion organization | -10.80 | -6.62 |
| GO:0006091 | | generation of precursor metabolites and energy | -9.81 | -5.94 |
| GO:0015980 | | energy derivation by oxidation of organic compounds | -7.22 | -3.88 |
| GO:0006839 | | mitochondrial transport | -6.98 | -3.86 |
| GO:0072594 | | establishment of protein localization to organelle | -6.81 | -3.86 |
| GO:0046034 | | ATP metabolic process | -6.80 | -3.86 |
| GO:0051640 | | organelle localization | -6.77 | -3.86 |
| GO:0072657 | | protein localization to membrane | -6.58 | -3.78 |

SZ: schizophrenia; BP: bipolar disorder; MDD: major depressive disorder; AUD: alcohol use disorder; TUD: tobacco use disorder; AD: Alzheimer's disease; MCI: mild cognitive impairment; GO: gene ontology. *P* values are calculated using the cumulative hypergeometric distribution (Fisher's exact test) as implemented in Metascape.

**Table P.** The top 8 most significant GO terms for $\mathrm{PLS}_{T}$- gene sets in each diagnostic group.

| **GO term** | **Description** | **Log10 (*p*-value)** | **Log10(*q* value)** |
| --- | --- | --- | --- |
| **SZ** |  |  |  |
| GO:0061024 | membrane organization | -7.90 | -3.78 |
| GO:0006897 | endocytosis | -7.57 | -3.78 |
| GO:0098657 | import into cell | -7.48 | -3.78 |
| GO:0016071 | mRNA metabolic process | -6.93 | -3.45 |
| GO:0048284 | organelle fusion | -6.79 | -3.45 |
| GO:0098660 | inorganic ion transmembrane transport | -6.78 | -3.45 |
| GO:0098662 | inorganic cation transmembrane transport | -6.62 | -3.35 |
| GO:0034660 | ncRNA metabolic process | -6.51 | -3.33 |
| **BP** |  |  |  |
| GO:0006897 | endocytosis | -10.06 | -5.88 |
| GO:0098657 | import into cell | -9.47 | -5.59 |
| GO:0051223 | regulation of protein transport | -7.14 | -3.49 |
| GO:0005975 | carbohydrate metabolic process | -7.07 | -3.49 |
| GO:0007264 | small GTPase mediated signal transduction | -6.70 | -3.34 |
| GO:0000479 | endonucleolytic cleavage of tricistronic rRNA transcript | -6.61 | -3.34 |
| GO:0030490 | maturation of SSU-rRNA | -6.56 | -3.34 |
| GO:0009991 | response to extracellular stimulus | -6.56 | -3.34 |
| **MDD** |  |  |  |
| GO:0080135 | regulation of cellular response to stress | -6.09602 | -1.92 |
| GO:0021542 | dentate gyrus development | -5.19016 | -1.67 |
| GO:0010256 | endomembrane system organization | -5.04499 | -1.67 |
| GO:0007018 | microtubule-based movement | -4.95971 | -1.67 |
| GO:0046890 | regulation of lipid biosynthetic process | -4.93953 | -1.67 |
| GO:0061024 | membrane organization | -4.80646 | -1.67 |
| GO:0016567 | protein ubiquitination | -4.79672 | -1.67 |
| GO:0062012 | regulation of small molecule metabolic process | -4.50674 | -1.41 |
| **AUD** |  |  |  |
| GO:0098660 | inorganic ion transmembrane transport | -15.28 | -11.10 |
| GO:0098662 | inorganic cation transmembrane transport | -14.79 | -11.08 |
| GO:0098655 | monoatomic cation transmembrane transport | -14.78 | -11.08 |
| GO:0099536 | synaptic signaling | -12.58 | -9.30 |
| GO:0016310 | phosphorylation | -10.15 | -7.05 |
| GO:0006468 | protein phosphorylation | -8.99 | -5.93 |
| GO:1903530 | regulation of secretion by cell | -8.74 | -5.70 |
| GO:0050804 | modulation of chemical synaptic transmission | -8.00 | -5.03 |
| **TUD** |  |  |  |
| GO:0099536 | synaptic signaling | -10.81 | -6.63496 |
| GO:0030001 | metal ion transport | -8.05 | -4.71351 |
| GO:0098660 | inorganic ion transmembrane transport | -7.50 | -4.27869 |
| GO:0070201 | regulation of establishment of protein localization | -6.88 | -3.7807 |
| GO:0061001 | regulation of dendritic spine morphogenesis | -6.67 | -3.60462 |
| GO:0032253 | dense core granule localization | -6.46 | -3.49042 |
| GO:0044057 | regulation of system process | -6.44 | -3.49042 |
| GO:0050808 | synapse organization | -6.08 | -3.20019 |
| **AD** |  |  |  |
| GO:0008380 | RNA splicing | -8.58173 | -4.74 |
| GO:0006974 | DNA damage response | -8.51464 | -4.74 |
| GO:0006259 | DNA metabolic process | -8.44185 | -4.74 |
| GO:0006397 | mRNA processing | -8.27954 | -4.70 |
| GO:0016071 | mRNA metabolic process | -7.99157 | -4.51 |
| GO:0006325 | chromatin organization | -6.56159 | -3.23 |
| GO:0006264 | mitochondrial DNA replication | -5.0537 | -1.96 |
| GO:0032870 | cellular response to hormone stimulus | -4.72676 | -1.70 |
| **MCI** |  |  |  |
| GO:0006259 | DNA metabolic process | -6.81 | -2.63 |
| GO:0006974 | DNA damage response | -5.79 | -2.09 |
| GO:0051028 | mRNA transport | -5.82 | -2.09 |

SZ: schizophrenia; BP: bipolar disorder; MDD: major depressive disorder; AUD: alcohol use disorder; TUD: tobacco use disorder; AD: Alzheimer's disease; MCI: mild cognitive impairment; GO: gene ontology. *P* values are calculated using the cumulative hypergeometric distribution (Fisher's exact test) as implemented in Metascape.

**Table Q.** Prediction performance with and without site regression in the training set after age correction.

| **Site effect** | **ADHD** | | | **ASD** | | | **SZ** | | | **BP** | | | **MDD** | | |
| --- | --- | --- | --- | --- | --- | --- | --- | --- | --- | --- | --- | --- | --- | --- | --- |
|  | *r* | MAE | R² | *r* | MAE | R² | *r* | MAE | R² | *r* | MAE | R² | *r* | MAE | R² |
| **Without site regression** | 0.92 | 1.15 | 0.83 | 0.93 | 3.10 | 0.84 | 0.91 | 3.60 | 0.80 | 0.91 | 3.75 | 0.80 | 0.92 | 3.69 | 0.81 |
| **With site regression** | 0.92 | 1.17 | 0.82 | 0.92 | 3.21 | 0.83 | 0.92 | 3.61 | 0.80 | 0.91 | 3.72 | 0.79 | 0.91 | 3.76 | 0.80 |
| **Site effect** | **AUD** | | | **TUD** | | | **A&TUD** | | | **AD** | | | **MCI** | | |
|  | *r* | MAE | R² | *r* | MAE | R² | *r* | MAE | R² | *r* | MAE | R² | *r* | MAE | R² |
| **Without site regression** | 0.89 | 2.78 | 0.79 | 0.89 | 2.72 | 0.80 | 0.90 | 2.70 | 0.79 | 0.90 | 2.40 | 0.76 | 0.90 | 2.43 | 0.76 |
| **With site regression** | 0.87 | 2.97 | 0.72 | 0.87 | 2.85 | 0.74 | 0.91 | 2.76 | 0.78 | 0.89 | 2.42 | 0.75 | 0.89 | 2.44 | 0.75 |

ADHD: attention-deficit/hyperactivity disorder; ASD: autism spectrum disorder; SZ: schizophrenia; BP: bipolar disorder; MDD: major depressive disorder; AUD: alcohol use disorder; TUD: tobacco use disorder; A&TUD: AUD and TUD; AD: Alzheimer's disease; MCI: mild cognitive impairment; MAE: mean absolute error; R²: coefficient of determination.

**Table R.** Prediction performance with and without site regression in the testing sets after age correction.

| **Site effect** | **ADHD** | | | **ASD** | | | **SZ** | | | **BP** | | | **MDD** | | |
| --- | --- | --- | --- | --- | --- | --- | --- | --- | --- | --- | --- | --- | --- | --- | --- |
|  | *r* | MAE | R² | *r* | MAE | R² | *r* | MAE | R² | *r* | MAE | R² | *r* | MAE | R² |
| **Without site regression** | 0.76 | 1.45 | 0.58 | 0.83 | 3.18 | 0.65 | 0.86 | 5.20 | 0.67 | 0.88 | 4.88 | 0.73 | 0.81 | 4.94 | 0.62 |
| **With site regression** | 0.73 | 1.47 | 0.59 | 0.84 | 3.22 | 0.65 | 0.85 | 5.63 | 0.62 | 0.87 | 5.08 | 0.71 | 0.81 | 4.97 | 0.61 |
| **Site effect** | **AUD** | | | **TUD** | | | **A&TUD** | | | **AD** | | | **MCI** | | |
|  | *r* | MAE | R² | *r* | MAE | R² | *r* | MAE | R² | *r* | MAE | R² | *r* | MAE | R² |
| **Without site regression** | 0.81 | 4.06 | 0.64 | 0.82 | 4.46 | 0.64 | 0.79 | 3.98 | 0.60 | 0.86 | 2.98 | 0.73 | 0.89 | 2.70 | 0.79 |
| **With site regression** | 0.80 | 4.26 | 0.62 | 0.83 | 4.24 | 0.66 | 0.77 | 4.67 | 0.54 | 0.86 | 3.01 | 0.73 | 0.89 | 2.85 | 0.77 |

ADHD: attention-deficit/hyperactivity disorder; ASD: autism spectrum disorder; SZ: schizophrenia; BP: bipolar disorder; MDD: major depressive disorder; AUD: alcohol use disorder; TUD: tobacco use disorder; A&TUD: AUD and TUD; AD: Alzheimer's disease; MCI: mild cognitive impairment; MAE: mean absolute error; R²: coefficient of determination.

**Table S.** Site-wise prediction performance estimated using LOSO validation in training sets after age correction.

| **Site index** | **ADHD** | | | **ASD** | | | **SZ** | | | **BP** | | | **MDD** | | |
| --- | --- | --- | --- | --- | --- | --- | --- | --- | --- | --- | --- | --- | --- | --- | --- |
|  | *r* | MAE | R² | *r* | MAE | R² | *r* | MAE | R² | *r* | MAE | R² | *r* | MAE | R² |
| **Site1** | 0.93 | 1.17 | 0.84 | 0.95 | 2.75 | 0.88 | 0.94 | 3.46 | 0.84 | 0.87 | 4.26 | 0.69 | 0.92 | 3.51 | 0.83 |
| **Site2** | 0.89 | 1.34 | 0.74 | 0.93 | 3.01 | 0.85 | 0.92 | 3.76 | 0.79 | 0.93 | 3.78 | 0.83 | 0.90 | 3.65 | 0.80 |
| **Site3** | 0.91 | 1.22 | 0.79 | 0.93 | 2.95 | 0.80 | 0.91 | 3.76 | 0.79 | 0.92 | 3.60 | 0.82 | 0.92 | 3.39 | 0.77 |
| **Site4** | 0.92 | 1.15 | 0.82 | 0.91 | 3.35 | 0.81 | 0.88 | 3.88 | 0.76 | 0.92 | 4.08 | 0.80 | 0.92 | 3.77 | 0.82 |
| **Site5** | 0.93 | 1.29 | 0.85 | 0.93 | 3.36 | 0.85 | 0.91 | 3.91 | 0.81 | 0.91 | 3.60 | 0.80 | 0.92 | 3.52 | 0.81 |
| **Site6** | / | / | / | 0.89 | 3.55 | 0.78 | 0.91 | 3.69 | 0.79 | 0.94 | 3.28 | 0.83 | 0.91 | 4.37 | 0.79 |
| **Site7** | / | / | / | 0.95 | 2.95 | 0.87 | 0.91 | 3.69 | 0.80 | 0.90 | 4.00 | 0.76 | 0.92 | 4.32 | 0.81 |
| **Site8** | / | / | / | 0.93 | 2.98 | 0.45 | 0.91 | 3.24 | 0.80 | 0.93 | 3.64 | 0.85 | 0.93 | 3.82 | 0.84 |
| **Site9** | / | / | / | 0.95 | 2.95 | 0.88 | 0.90 | 3.94 | 0.75 | 0.90 | 3.92 | 0.78 | 0.93 | 3.57 | 0.81 |
| **Site10** | / | / | / | 0.88 | 3.92 | 0.73 | 0.91 | 3.51 | 0.80 | 0.91 | 3.59 | 0.81 | 0.91 | 3.36 | 0.75 |
| **Site11** | / | / | / | / | / | / | 0.92 | 3.36 | 0.81 | 0.91 | 3.72 | 0.78 | 0.90 | 4.02 | 0.78 |
| **Site12** | / | / | / | / | / | / | 0.93 | 3.13 | 0.83 | 0.90 | 3.68 | 0.78 | 0.90 | 4.16 | 0.77 |
| **Site13** | / | / | / | / | / | / | 0.90 | 3.67 | 0.78 | 0.92 | 3.65 | 0.80 | 0.89 | 3.84 | 0.74 |
| **CV(%)** | **1.7** | **6.6** | **5.8** | **2.7** | **11.3** | **6.0** | **1.5** | **7.2** | **3.2** | **2.0** | **6.8** | **5.0** | **1.3** | **8.9** | **3.9** |
| **Site index** | **AUD** | | | **TUD** | | | **A&TUD** | | | **AD** | | | **MCI** | | |
|  | *r* | MAE | R² | *r* | MAE | R² | *r* | MAE | R² | *r* | MAE | R² | *r* | MAE | R² |
| **Site1** | 0.89 | 3.02 | 0.75 | 0.92 | 2.74 | 0.82 | 0.92 | 2.74 | 0.82 | 0.90 | 2.41 | 0.76 | 0.90 | 2.41 | 0.76 |
| **Site2** | 0.92 | 2.90 | 0.72 | 0.91 | 3.14 | 0.75 | 0.91 | 3.14 | 0.75 | 0.90 | 2.42 | 0.76 | 0.90 | 2.42 | 0.76 |
| **Site3** | 0.90 | 2.94 | 0.77 | 0.89 | 2.77 | 0.77 | 0.89 | 2.77 | 0.77 | 0.89 | 2.48 | 0.75 | 0.89 | 2.48 | 0.75 |
| **Site4** | 0.91 | 2.66 | 0.80 | 0.90 | 2.74 | 0.76 | 0.90 | 2.74 | 0.76 | 0.90 | 2.42 | 0.76 | 0.90 | 2.42 | 0.76 |
| **Site5** | 0.90 | 2.89 | 0.78 | 0.89 | 2.45 | 0.75 | 0.89 | 2.45 | 0.75 | 0.89 | 2.44 | 0.75 | 0.89 | 2.44 | 0.75 |
| **Site6** | 0.90 | 2.58 | 0.79 | 0.89 | 2.74 | 0.76 | 0.89 | 2.74 | 0.76 | 0.90 | 2.46 | 0.76 | 0.90 | 2.46 | 0.76 |
| **Site7** | 0.92 | 2.72 | 0.83 | 0.91 | 2.89 | 0.79 | 0.91 | 2.89 | 0.79 | / | / | / | / | / | / |
| **Site8** | 0.90 | 2.84 | 0.76 | 0.94 | 2.76 | 0.81 | 0.94 | 2.76 | 0.81 | / | / | / | / | / | / |
| **Site9** | 0.92 | 2.84 | 0.80 | 0.92 | 3.07 | 0.78 | 0.92 | 3.07 | 0.78 | / | / | / | / | / | / |
| **Site10** | 0.91 | 3.11 | 0.80 | 0.88 | 3.21 | 0.76 | 0.88 | 3.21 | 0.76 | / | / | / | / | / | / |
| **Site11** | 0.92 | 2.96 | 0.81 | 0.89 | 3.09 | 0.73 | 0.89 | 3.09 | 0.73 | / | / | / | / | / | / |
| **Site12** | 0.90 | 2.91 | 0.75 | 0.92 | 2.76 | 0.82 | 0.92 | 2.76 | 0.82 | / | / | / | / | / | / |
| **Site13** | 0.91 | 2.51 | 0.80 | 0.92 | 2.69 | 0.80 | 0.92 | 2.69 | 0.80 | / | / | / | / | / | / |
| **CV(%)** | **1.1** | **6.1** | **3.8** | **1.9** | **7.6** | **3.9** | **1.9** | **7.6** | **3.9** | **0.2** | **1.1** | **0.6** | **0.2** | **1.1** | **0.6** |

ADHD: attention-deficit/hyperactivity disorder; ASD: autism spectrum disorder; SZ: schizophrenia; BP: bipolar disorder; MDD: major depressive disorder; AUD: alcohol use disorder; TUD: tobacco use disorder; A&TUD: AUD and TUD; AD: Alzheimer's disease; MCI: mild cognitive impairment; MAE: mean absolute error; R²: coefficient of determination, CV: coefficient of variation.

**Table T.** Overall prediction performance estimated using 10-fold cross-validation and LOSO validation in the training sets after age correction.

| **Validation strategy** | **ADHD** | | | **ASD** | | | **SZ** | | | **BP** | | | **MDD** | | |
| --- | --- | --- | --- | --- | --- | --- | --- | --- | --- | --- | --- | --- | --- | --- | --- |
|  | *r* | MAE | R² | *r* | MAE | R² | *r* | MAE | R² | *r* | MAE | R² | *r* | MAE | R² |
| **10-Fold** | 0.92 | 1.15 | 0.83 | 0.93 | 3.10 | 0.84 | 0.91 | 3.60 | 0.80 | 0.91 | 3.75 | 0.80 | 0.92 | 3.69 | 0.81 |
| **LOSO** | 0.92 | 1.23 | 0.81 | 0.92 | 3.16 | 0.84 | 0.91 | 3.61 | 0.80 | 0.91 | 3.74 | 0.80 | 0.91 | 3.79 | 0.79 |
| **Validation strategy** | **AUD** | | | **TUD** | | | **A&TUD** | | | **AD** | | | **MCI** | | |
|  | *r* | MAE | R² | *r* | MAE | R² | *r* | MAE | R² | *r* | MAE | R² | *r* | MAE | R² |
| **10-Fold** | 0.89 | 2.78 | 0.79 | 0.89 | 2.72 | 0.80 | 0.90 | 2.70 | 0.79 | 0.90 | 2.40 | 0.76 | 0.90 | 2.43 | 0.76 |
| **LOSO** | 0.91 | 2.86 | 0.78 | 0.91 | 2.84 | 0.78 | 0.91 | 2.75 | 0.79 | 0.90 | 2.44 | 0.76 | 0.90 | 2.44 | 0.75 |

ADHD: attention-deficit/hyperactivity disorder; ASD: autism spectrum disorder; SZ: schizophrenia; BP: bipolar disorder; MDD: major depressive disorder; AUD: alcohol use disorder; TUD: tobacco use disorder; A&TUD: AUD and TUD; AD: Alzheimer's disease; MCI: mild cognitive impairment; MAE: mean absolute error; R²: coefficient of determination, LOSO: leave-one-site-out.

**Table U.** Cross-site differences in PAD of HC from the testing sets.

| **Site index** | **ADHD** | **ASD** | **SZ** | **BP** | **MDD** | **AUD** | **TUD** | **A&TUD** | **AD** | **MCI** |
| --- | --- | --- | --- | --- | --- | --- | --- | --- | --- | --- |
|  | Mean PAD | Mean PAD | Mean PAD | Mean PAD | Mean PAD | Mean PAD | Mean PAD | Mean PAD | Mean PAD | Mean PAD |
| **Site1** | 0.32 | 0.55 | 1.24 | 0.10 | -1.16 | -1.80 | -3.01 | -1.07 | -1.31 | -0.99 |
| **Site2** | 0.48 | 0.72 | -0.98 | -1.33 | -0.22 | -1.40 | -2.96 | -0.54 | -1.43 | -0.97 |
| **Site3** | 0.50 | -0.53 | -1.09 | -0.34 | -1.12 | / | / | / | -2.09 | -1.51 |
| **Site4** | 0.30 | -0.12 | 0.85 | 1.26 | 0.78 | / | / | / | -3.24 | -0.91 |
| **Site5** | 0.30 | -0.70 | / | / | / | / | / | / | -0.53 | -0.62 |
| **Site6** | 0.18 | -0.77 | / | / | / | / | / | / | / | -2.44 |
| **Site7** | / | 0.21 | / | / | / | / | / | / | / | -2.49 |
| **Site8** | / | -1.13 | / | / | / | / | / | / | / | -0.13 |
| **Site9** | / | 0.31 | / | / | / | / | / | / | / | -0.86 |
| **Site10** | / | -0.18 | / | / | / | / | / | / | / | -0.07 |
| **Site11** | / | -0.68 | / | / | / | / | / | / | / | -0.94 |
| **Site12** | / | 3.30 | / | / | / | / | / | / | / | -0.67 |
| **Site13** | / | -0.19 | / | / | / | / | / | / | / | -0.80 |
| **Site14** | / | -2.31 | / | / | / | / | / | / | / | -1.91 |
| **Site15** | / | 1.33 | / | / | / | / | / | / | / | -1.56 |
| **Site16** | / | -1.34 | / | / | / | / | / | / | / | -0.38 |
| **Site17** | / | -2.94 | / | / | / | / | / | / | / | -2.17 |
| **Site18** | / | / | / | / | / | / | / | / | / | -1.11 |
| **Site19** | / | / | / | / | / | / | / | / | / | -1.14 |
| **Site20** | / | / | / | / | / | / | / | / | / | -1.12 |
| **P value (unadjusted)** | **0.96** | **0.21** | **0.16** | **0.38** | **0.40** | **0.61** | **0.94** | **0.66** | **0.23** | **0.49** |
| **P value (adjusted)** | **0.85** | **0.49** | **0.09** | **0.31** | **0.23** | **0.19** | **0.75** | **0.98** | **0.29** | **0.51** |

ADHD: attention-deficit/hyperactivity disorder; ASD: autism spectrum disorder; SZ: schizophrenia; BP: bipolar disorder; MDD: major depressive disorder; AUD: alcohol use disorder; TUD: tobacco use disorder; A&TUD: AUD and TUD; AD: Alzheimer's disease; MCI: mild cognitive impairment; PAD: predicted age difference. The adjusted/unadjusted *p* values are calculated using Analysis of Covariance **(**ANCOVA) with/without age, age², and sex as covariates.
