# Supplementary material for: Brain aging patterns among nine neurological disorders: A case-control study
Source: PLoS Med. 2026 Jul 21;23(7):e1004860. doi: 10.1371/journal.pmed.1004860 (PMC13387544; doi:10.1371/journal.pmed.1004860)
Supplement: S3 Appendix — Fig A. The scatterplot of ages used in each control subgroup. ADHD, attention-deficit/hyperactivity disorder; ASD, autism spectrum disorder; SZ, schizophrenia; BP, bipolar disorder; MDD, major depressive disorder; AUD, alcohol use disorder; TUD, tobacco use disorder; A&TUD, AUD and TUD; AD, Alzheimer’s disease; MCI, mild cognitive impairment. Fig B. Samples construction details for training and testing. HCP, Human Connectome Project; GSP, Brain Genomics Superstruct Project; UKB, UK Biobank; ABIDE II, Autism Brain Imaging Data Exchange; BSNIP-I, Bipolar and Schizophrenia Network for Intermediate Phenotypes; ADNI, Alzheimer’s Disease Neuroimaging Initiative; ADHD, attention-deficit/hyperactivity disorder; ASD, autism spectrum disorder; SZ, schizophrenia; BP, bipolar disorder; MDD, major depressive disorder; AUD, alcohol use disorder; TUD, tobacco use disorder; A&TUD, AUD and TUD; AD, Alzheimer’s disease; MCI, mild cognitive impairment; HCs, healthy controls. Fig C. Model performance in training sets. Performance metrics of the brain age prediction model in the training sets, shown (a) without and (b) after age correction, including the Pearson correlation (r) between predicted brain age and chronological age, mean absolute error (MAE), and coefficient of determination (R2). The solid line indicates the linear regression fit. Notably, the training sets for different diagnostic groups were independently constructed by matching the age distribution of each test cohort. Consequently, when multiple diagnostic groups share similar or identical age ranges (e.g., MCI and AD; AUD, TUD, and A&TUD), their corresponding training sets may contain substantially overlapping or even identical samples, leading to visually similar scatter plots. The overlap rates between training sets for each pair of diagnostic groups are provided in Table D in S2 Appendix. ADHD, attention-deficit/hyperactivity disorder; ASD, autism spectrum disorder; SZ, schizophrenia; BP, bipolar disorder; MDD, majo [file pmed.1004860.s004.docx]

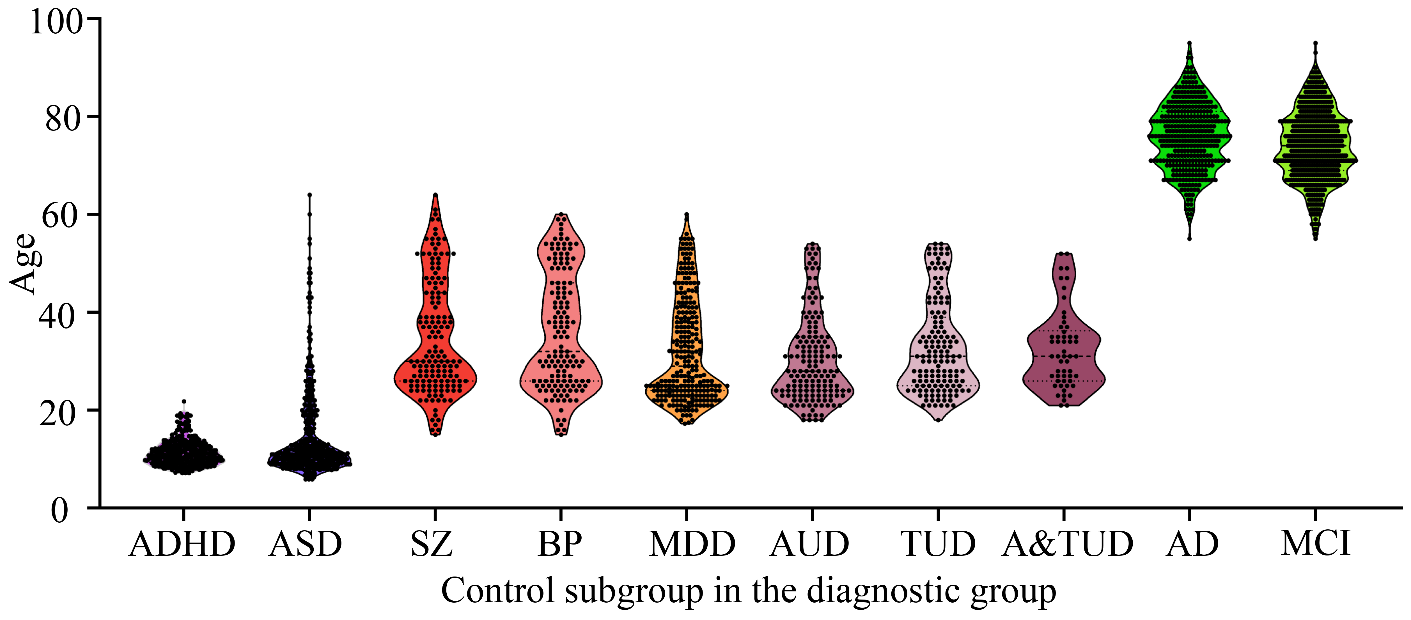


**Figure A.** **The scatterplot of ages used in each control subgroup.** ADHD: attention-deficit/hyperactivity disorder; ASD: autism spectrum disorder; SZ: schizophrenia; BP: bipolar disorder; MDD: major depressive disorder; AUD: alcohol use disorder; TUD: tobacco use disorder; A&TUD: AUD and TUD; AD: Alzheimer's disease; MCI: mild cognitive impairment.


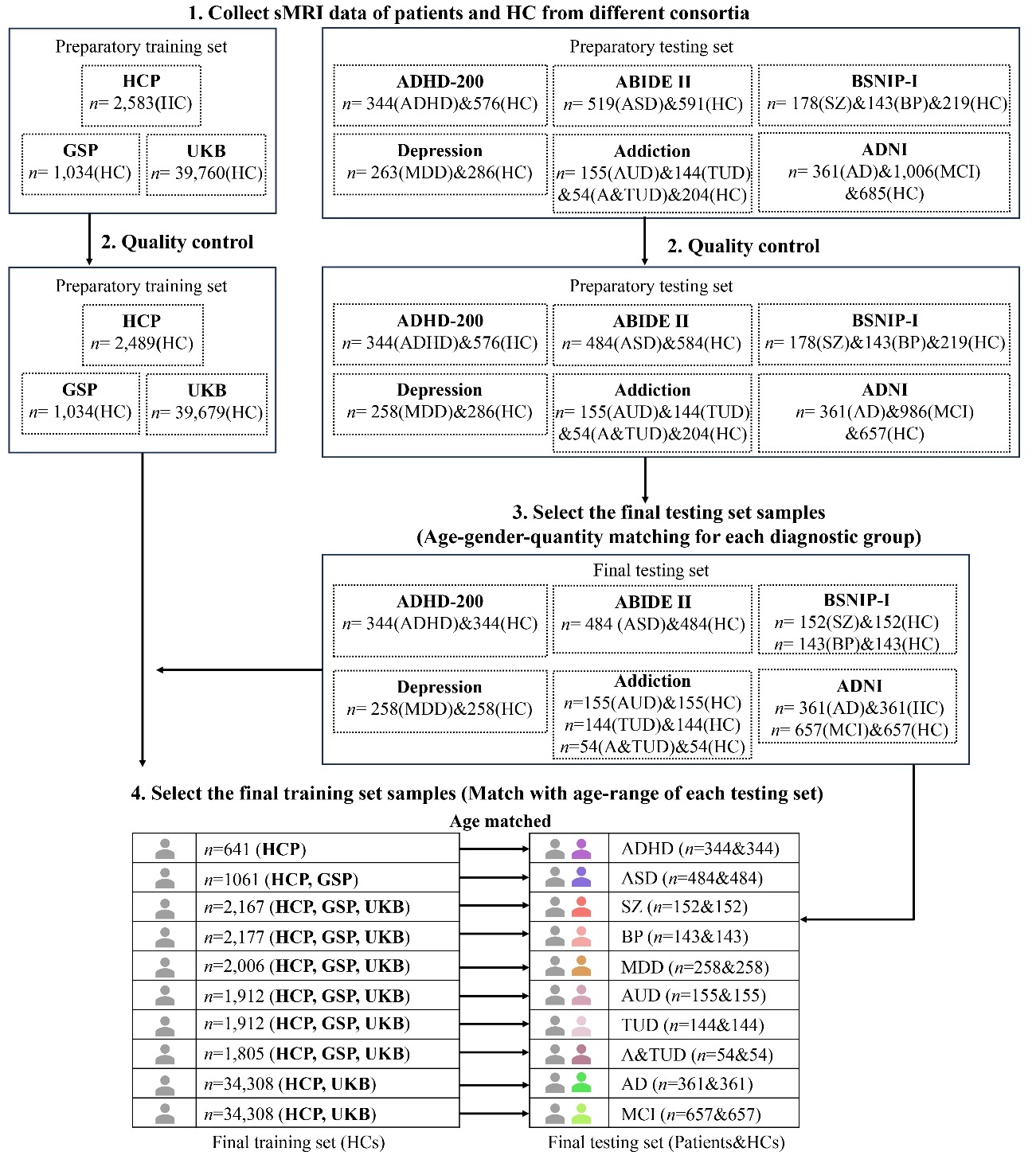


**Figure B. Samples construction details for training and testing**. HCP: Human Connectome Project; GSP: Brain Genomics Superstruct Project; UKB: UK Biobank; ABIDE II: Autism Brain Imaging Data Exchange; BSNIP-I: Bipolar and Schizophrenia Network for Intermediate Phenotypes; ADNI: Alzheimer's Disease Neuroimaging Initiative; ADHD: attention-deficit/hyperactivity disorder; ASD: autism spectrum disorder; SZ: schizophrenia; BP: bipolar disorder; MDD: major depressive disorder; AUD: alcohol use disorder; TUD: tobacco use disorder; A&TUD: AUD and TUD; AD: Alzheimer's disease; MCI: mild cognitive impairment; HCs: healthy controls.

**
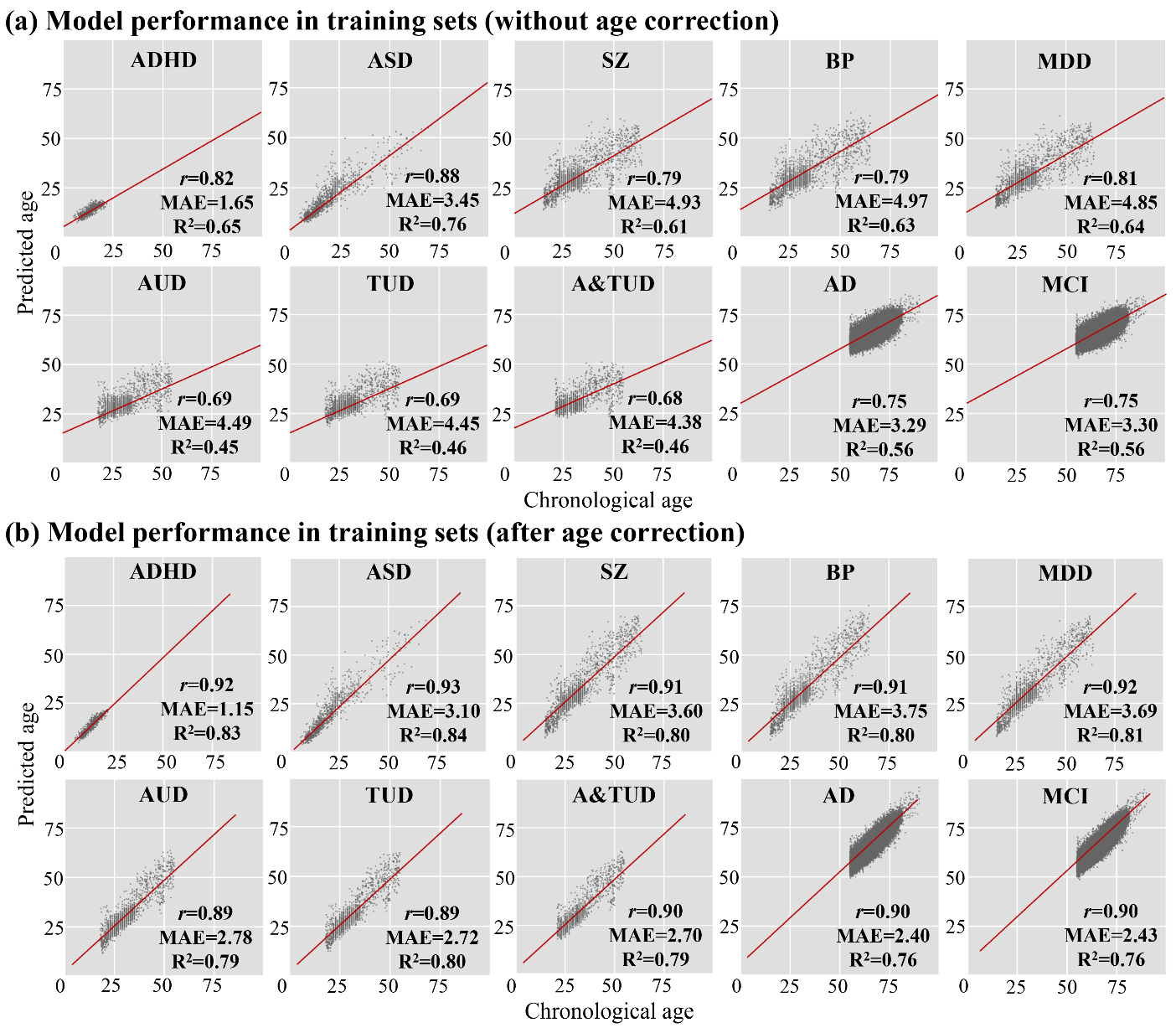
**

**Figure C. Model performance in training sets**. Performance metrics of the brain age prediction model in the training sets, shown **(a)** without and **(b)** after age correction, including the Pearson correlation (*r*) between predicted brain age and chronological age, mean absolute error (MAE), and coefficient of determination (R²). The solid line indicates the linear regression fit. Notably, the training sets for different diagnostic groups were independently constructed by matching the age distribution of each test cohort. Consequently, when multiple diagnostic groups share similar or identical age ranges (e.g., MCI and AD; AUD, TUD, and A&TUD), their corresponding training sets may contain substantially overlapping or even identical samples, leading to visually similar scatter plots. The overlap rates between training sets for each pair of diagnostic groups are provided in **Table D in S2 Appendix.** ADHD: attention-deficit/hyperactivity disorder; ASD: autism spectrum disorder; SZ: schizophrenia; BP: bipolar disorder; MDD: major depressive disorder; AUD: alcohol use disorder; TUD: tobacco use disorder; A&TUD: AUD and TUD; AD: Alzheimer's disease; MCI: mild cognitive impairment.

**
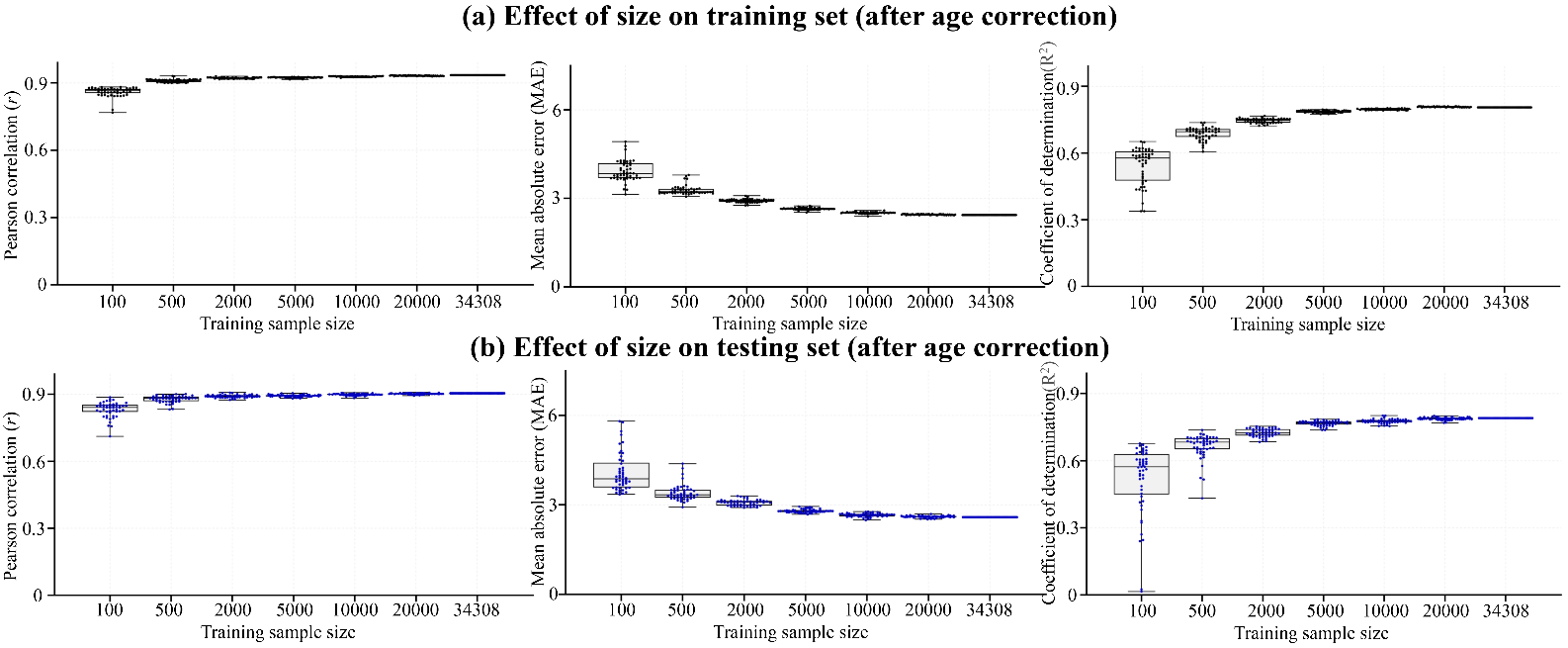
**

**Figure D. Impact of training sample size on the performance of brain age prediction**. We used the training-test set corresponding to AD to evaluate the impact of training sample size on brain age prediction model performance (including *r*, MAE and R^2^), owing to its large sample size. A total of 34308 HCs were included as the full training samples, and 361 HCs from ADNI were included as the test samples (excluding 361 ADs as without ground truth: individuals with brain disorder may be not matched brain age and chronological age, **Fig 3a**). Fifty random subsets of full training set include 100, 500, 2000, 5000, 10000 and 20000 individuals were drawn, and corresponding models cross-validated and applied to testing samples. We also displayed the results from the full training samples (*n*=34308). The maxima, upper quartile, median, lower quartile and minima were displayed in the box plots. Panel **(a)** shows the results for the training sets, and panel **(b)** shows the results for the test sets. With increasing training sample size, performance of the models increased, with less variation across runs. In boxplots, the lower, middle, and upper bounds of the box represent the first quartile, median, and third quartile, respectively. The lower and upper whiskers represent the minimum and maximum values, respectively. MAE: mean absolute error; R^2^: coefficient of determination.


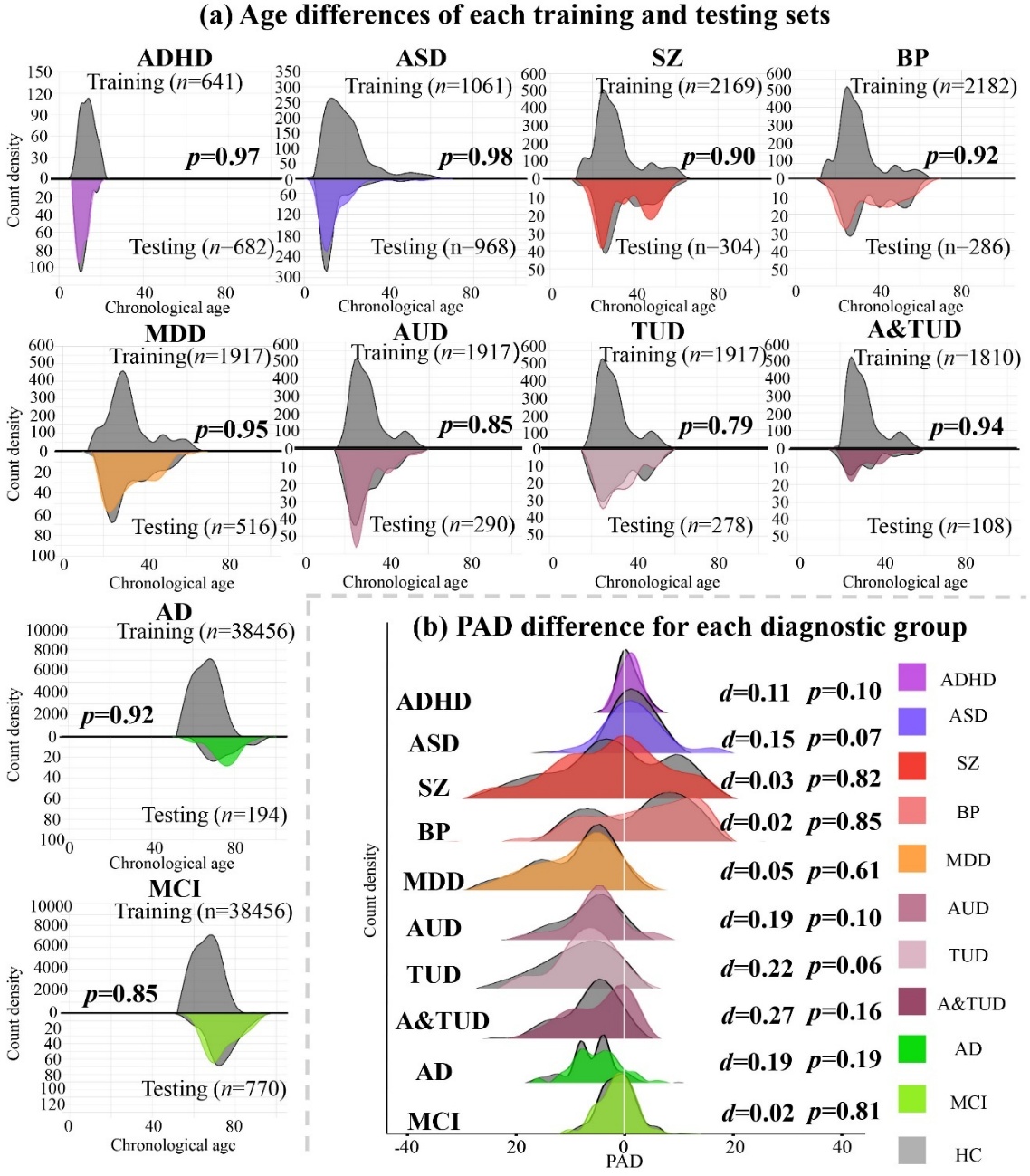


**Figure E. Age distributions and the PAD difference for each diagnostic group based on fMRI**. **(a)** The sample size and age distribution of training (up) and testing (down) sets for each diagnostic group with fMRI data. The gray shading under each diagnostic group represents its age-, sex- and number-matched control group. *P* value represents the group difference of age between diagnostic group and HC calculated by two sample *t*-test. The testing samples comprised 341 ADHDs/341 HCs; 484 ASDs/484 HCs; 152 SZs/152 HCs; 143 BPs/143 HCs; 258 MDDs/258 HCs; 145 AUDs/145 HCs; 139 TUDs/139 HCs; 54 A&TUDs/54 HCs; 97 ADs/97HCs; and 385 MCIs/385 HCs. The training samples comprised 9307 HCs for ADHD, 11523 HCs for ASD, 2169 HCs for SZ, 2182 HCs for BP, 1917 HCs for MDD, AUD and TUD,1810 HCs for A&TUD, 38456 HCs for AD and MCI, which selected from HCP, GSP and UKB with matched age-range for each diagnostic group. **(b)** The PAD difference between patient and HC. Cohen’s *d* effect sizes accounting for age, age^2^, sex and site and two-sided *p* values from linear model-based *t*-tests are provided. ADHD: attention-deficit/hyperactivity disorder; ASD: autism spectrum disorder; SZ: schizophrenia; BP: bipolar disorder; MDD: major depressive disorder; AUD: alcohol use disorder; TUD: tobacco use disorder; A&TUD: AUD and TUD; AD: Alzheimer's disease; MCI: mild cognitive impairment; HC: healthy control.

**
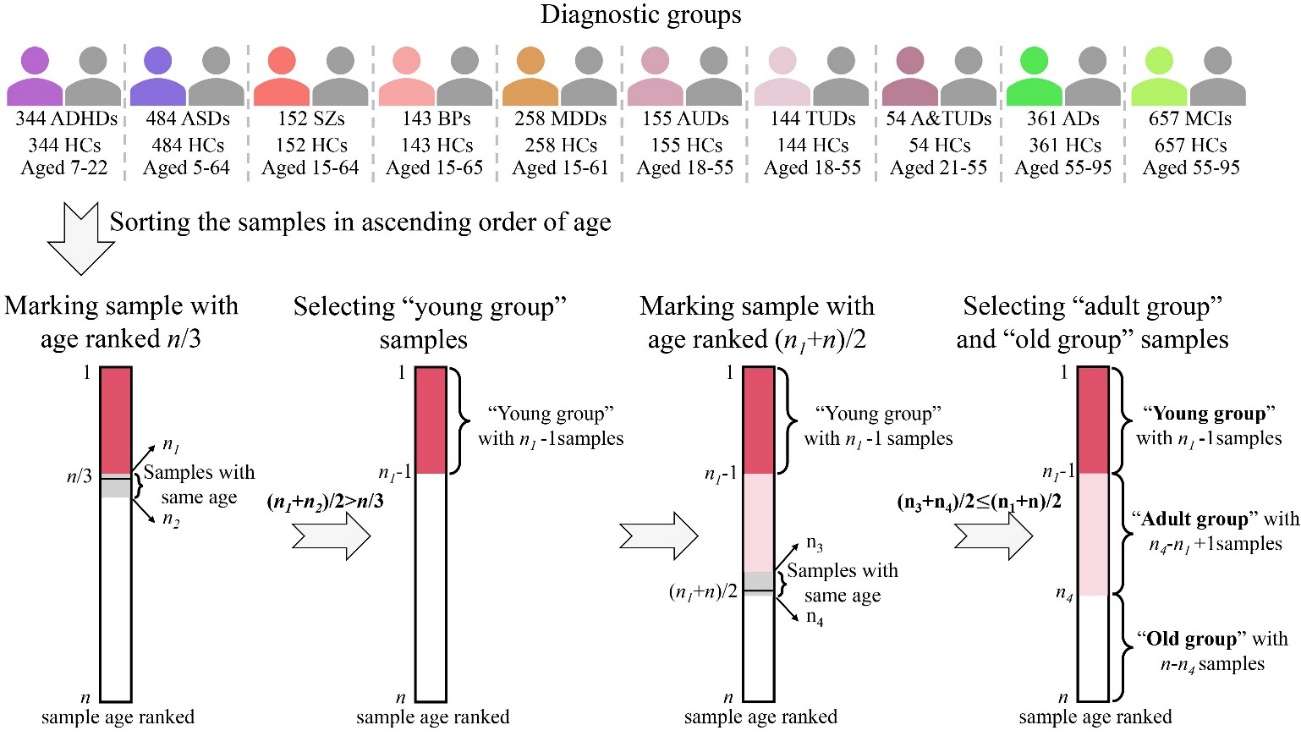
**

**Figure F. An example of criteria for dividing each diagnostic group into different age groups**. In any given diagnostic group, consisting of a total of *n* samples, we aim to allocate the sample sizes across different age groups as evenly as possible, while ensuring non-overlapping age ranges between groups. Firstly, sort these samples in ascending order of age and mark sample with age ranked $\frac{n}{3}$. Then, determine the upper ($n_{1}$) and lower ($n_{2}$) bounds on the ranking of samples with the same age as the marked $\frac{n}{3}$-th ranked sample. If $\frac{n_{1}+n_{2}}{2}$>$\frac{n}{3}$, the samples ranked 1 to ($n_{1}$-1) were divided into “young” group (this example, if $\frac{n_{1}+n_{2}}{2} \leq\frac{n}{3}$, the samples ranked 1 to $n_{2}$ were divided into “young” group). Then, mark sample with age ranked $\frac{n_{1}+n}{2}$, and determine the upper ($n_{3}$) and lower ($n_{4}$) bounds on the ranking of samples with the same age as the marked $\frac{n_{1}+n}{2}$-th ranked sample. If $\frac{n_{3}+n_{4}}{2} \leq\frac{n_{1}+n}{2}$, the samples ranked $n_{1}$ to $n_{4}$ were divided into “adult” group, and the samples ranked ($n_{4}$-1) to $n$ were divided into “old” group (this example, if $\frac{n_{3}+n_{4}}{2} > \frac{n_{1}+n}{2}$, the samples ranked $n_{1}$ to ($n_{3}$-1) were divided into “adult” group and the samples ranked ($n_{3}$) to $n$ were divided into “old” group). ADHD: attention-deficit/hyperactivity disorder; ASD: autism spectrum disorder; SZ: schizophrenia; BP: bipolar disorder; MDD: major depressive disorder; AUD: alcohol use disorder; TUD: tobacco use disorder; A&TUD: AUD and TUD; AD: Alzheimer's disease; MCI: mild cognitive impairment; HC: healthy control.


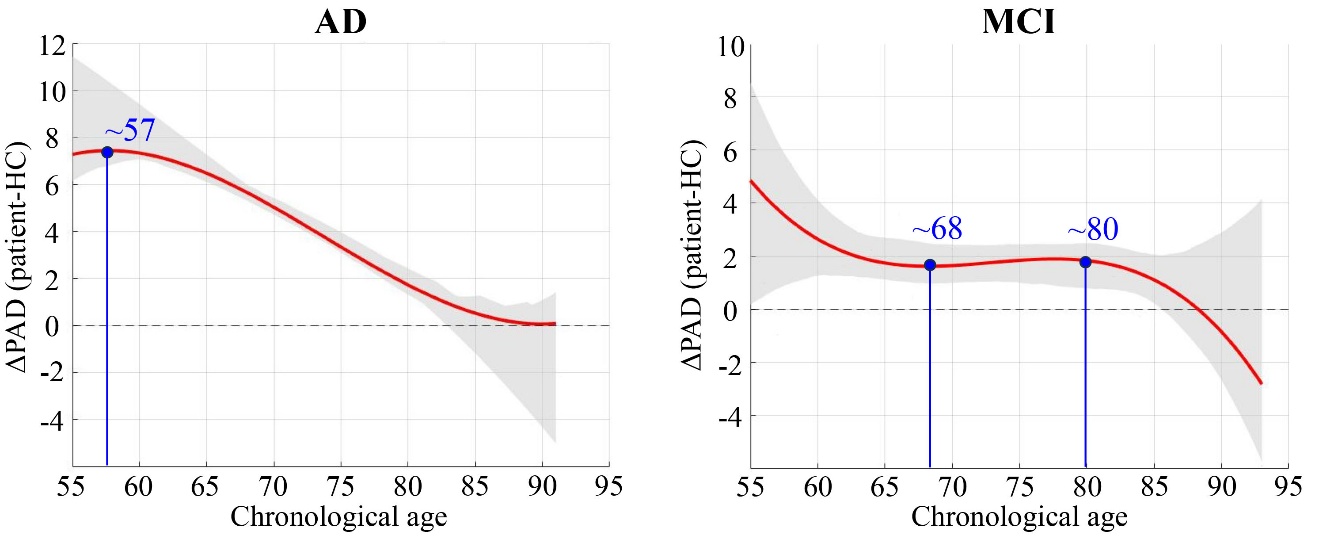


**Figure G. Age-related trajectories of ΔPAD in AD and MCI**. ΔPAD was defined as the mean PAD in patients minus the mean PAD in healthy controls within each age window. Smoothed curves illustrate the age-dependent variation of ΔPAD. The operationally defined divergence age corresponds to local extrema or inflection points in the fitted ΔPAD trajectories. For AD, a divergence point was observed at approximately 57 years. For MCI, divergence points were identified at approximately 68 and 80 years. AD: Alzheimer's disease; MCI: mild cognitive impairment; PAD: predicted age difference.

**
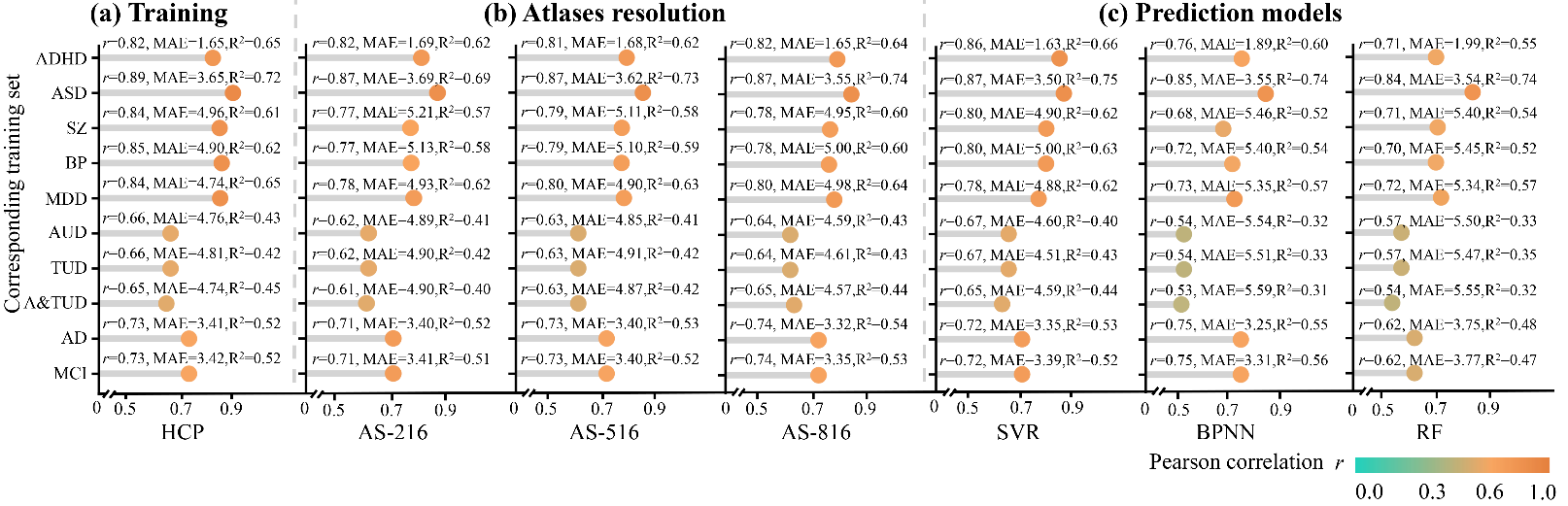
**

**Figure H. Predictive performance validation across datasets, atlases and different prediction models**. Ten-fold cross-validation was performed in each corresponding training set for the diagnostic group based on multiple **(a)** training sets, **(b)** brain atlas scales and **(c)** prediction models to test the predictive performance (without age correction). Color bar indicates Pearson correlation. HCP: Human Connectome Project; AS: augmented Schaefer; SVR: support vector regression; BPNN: back propagation neural network; RF: random forest. ADHD: attention-deficit/hyperactivity disorder; ASD: autism spectrum disorder; SZ: schizophrenia; BP: bipolar disorder; MDD: major depressive disorder; AUD: alcohol use disorder; TUD: tobacco use disorder; A&TUD: AUD and TUD; AD: Alzheimer's disease; MCI: mild cognitive impairment.

**
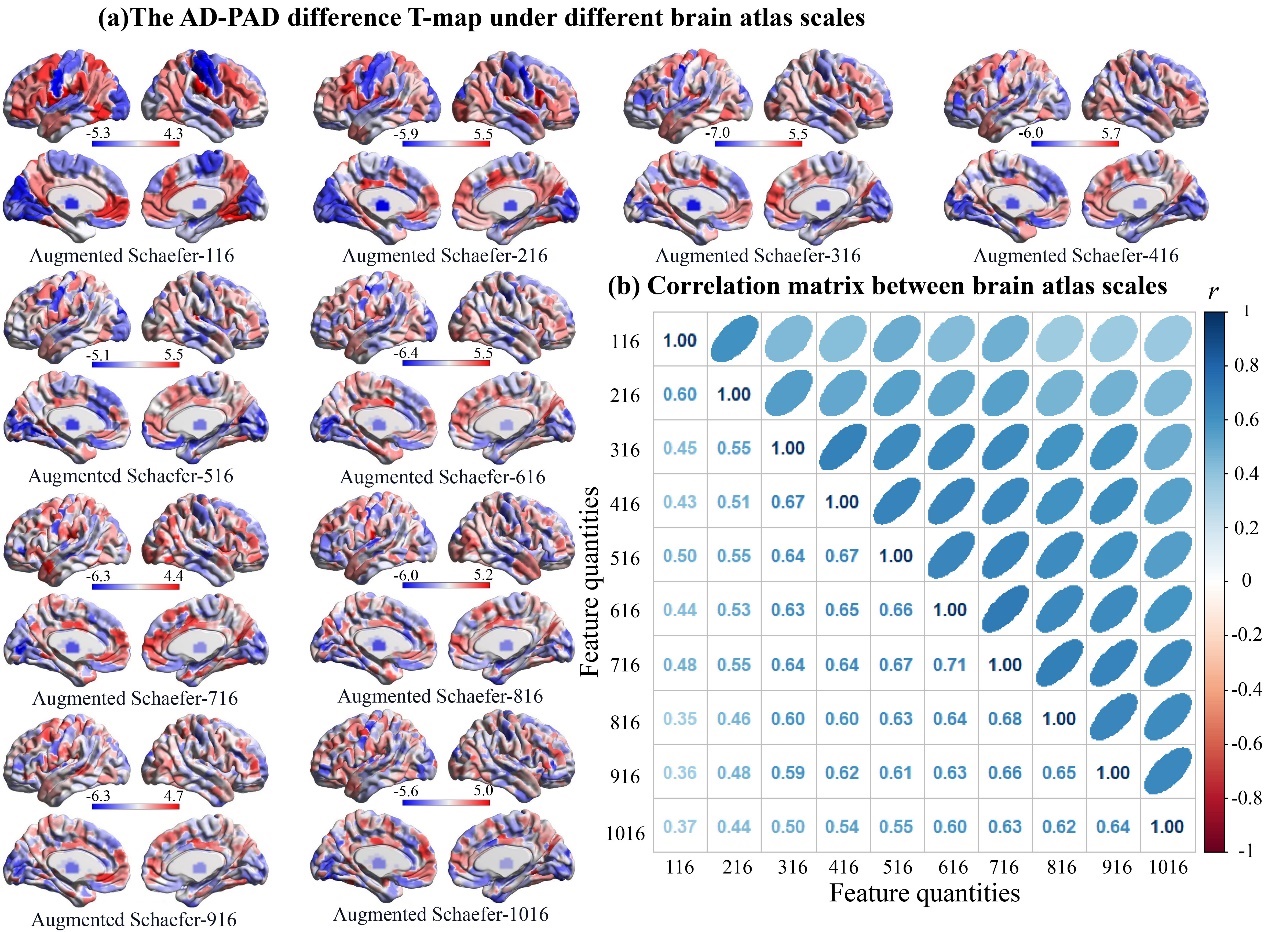
**

**Figure I. Repeatability verification of PAD difference T-map under different brain atlas scales using AD as an example**. **(a)** The AD-PAD difference T-map based on augmented Schaefer brain atlas from 116 ROIs to 1016 ROIs with intervals of 100. Color bar indicates statistical *t*-values of the interaction coefficients, and red/bule brain regions represent more stronger positive/negative associations between the contribution of identified brain features to predicting brain age and PAD in patient groups than in the HC. **(b)** The correlation matrix between brain atlas scales. All difference T-maps were unified parcellated into 1016 ROIs based on augmented Schaefer-1016 atlas and calculated the mean voxel value within each ROI. Therefore, we generated a 1016 $\times$ 1 dimensional vector for each difference T-map and then calculated the Pearson correlation between them. Colors bar indicates Pearson correlation *r* value. AD: Alzheimer's disease; PAD: predicted age difference.


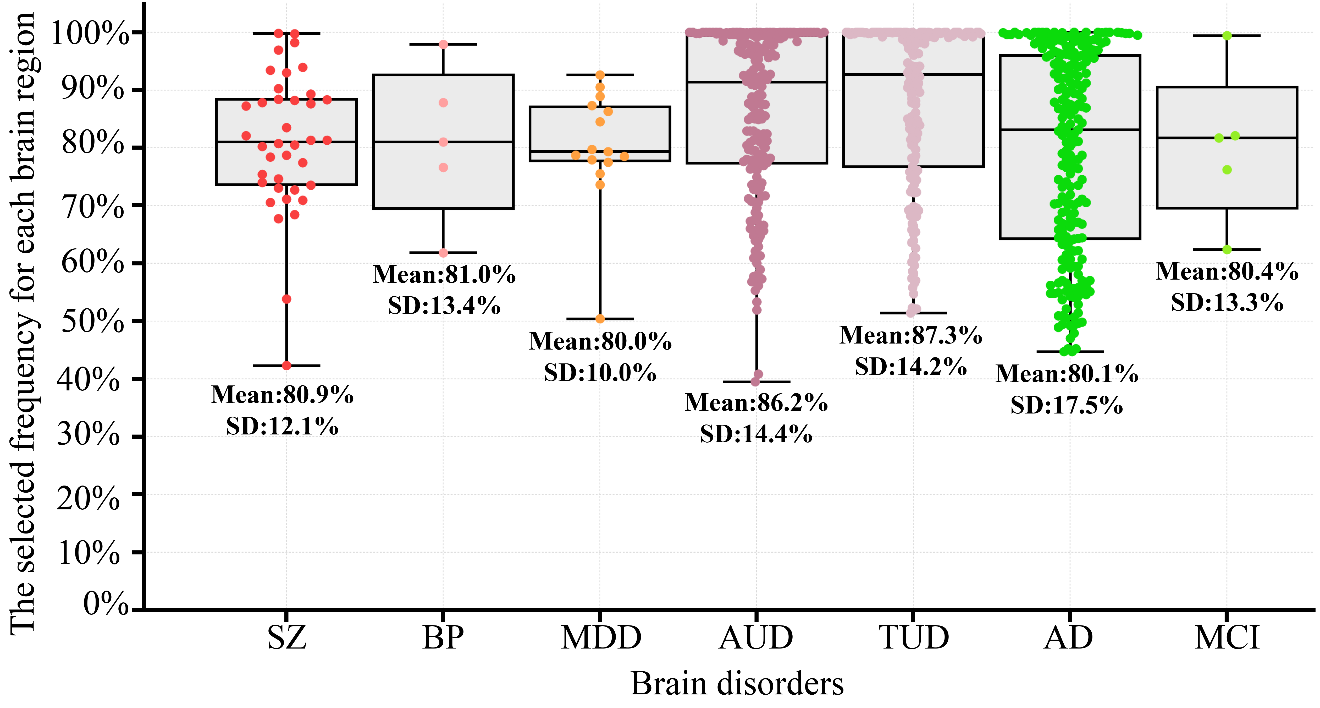


**Figure J. Bootstrapping analysis of the identified brain regions for each disorder.** Selection frequency of features identified in the main analysis across 1000 bootstrap resamples. In each iteration, 80% of the training dataset was randomly sampled without replacement and the same analytical pipeline was applied. Each dot represents one brain region identified in main analysis, and the y-axis indicates the proportion of bootstrap iterations in which the region was re-identified as contributing to the PAD difference. The boxplots illustrate the distribution of re-selection frequencies, where the lower, middle, and upper bounds of the box represent the first quartile, median, and third quartile, respectively. The lower and upper whiskers represent the minimum and maximum values, respectively. SZ: schizophrenia; BP: bipolar disorder; MDD: major depressive disorder; AUD: alcohol use disorder; TUD: tobacco use disorder; AD: Alzheimer's disease; MCI: mild cognitive impairment; SD: standard deviation.


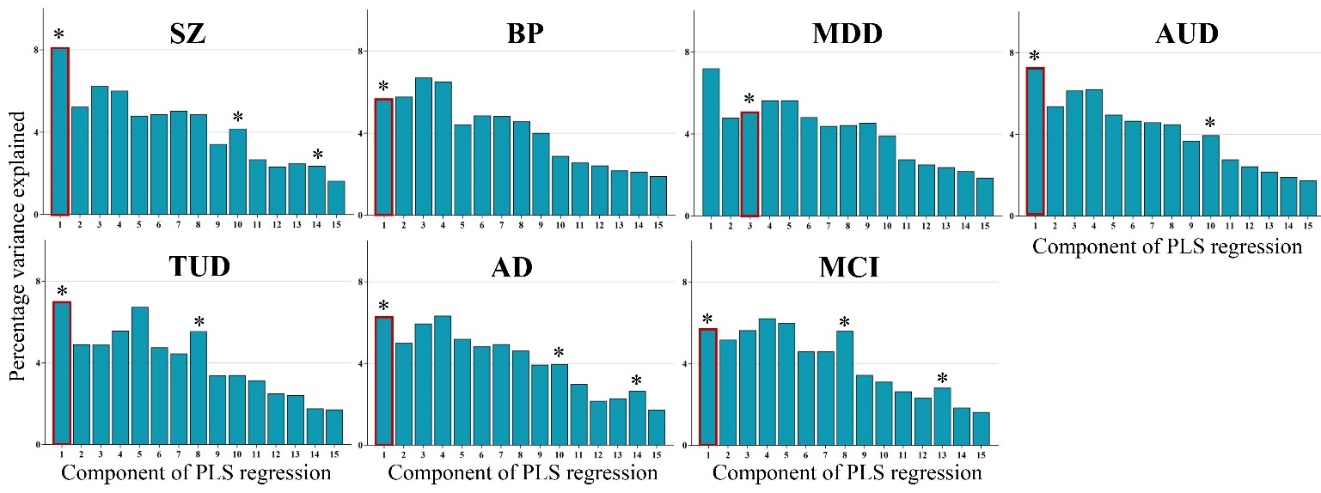


**Figure K. Variance in the PAD difference explained by all 15 PLS components in diagnostic group.** The * represents the variance was significantly greater than random level for this component (1000 permutation tests). Within the significance components, the first PLS component (PLS1) explained the most variance for SZ (8.1%), BP (5.4%), AUD (7.2%), TUD (7.0%), AD (6.3%) and MCI (5.3%), and the third PLS (PLS3) component for MDD (5.1%). Therefore, the PLS3 for MDD and PLS1 for other brain disorders were selected for further analyses. SZ: schizophrenia; BP: bipolar disorder; MDD: major depressive disorder; AUD: alcohol use disorder; TUD: tobacco use disorder; AD: Alzheimer's disease; MCI: mild cognitive impairment; PLS: partial least square.


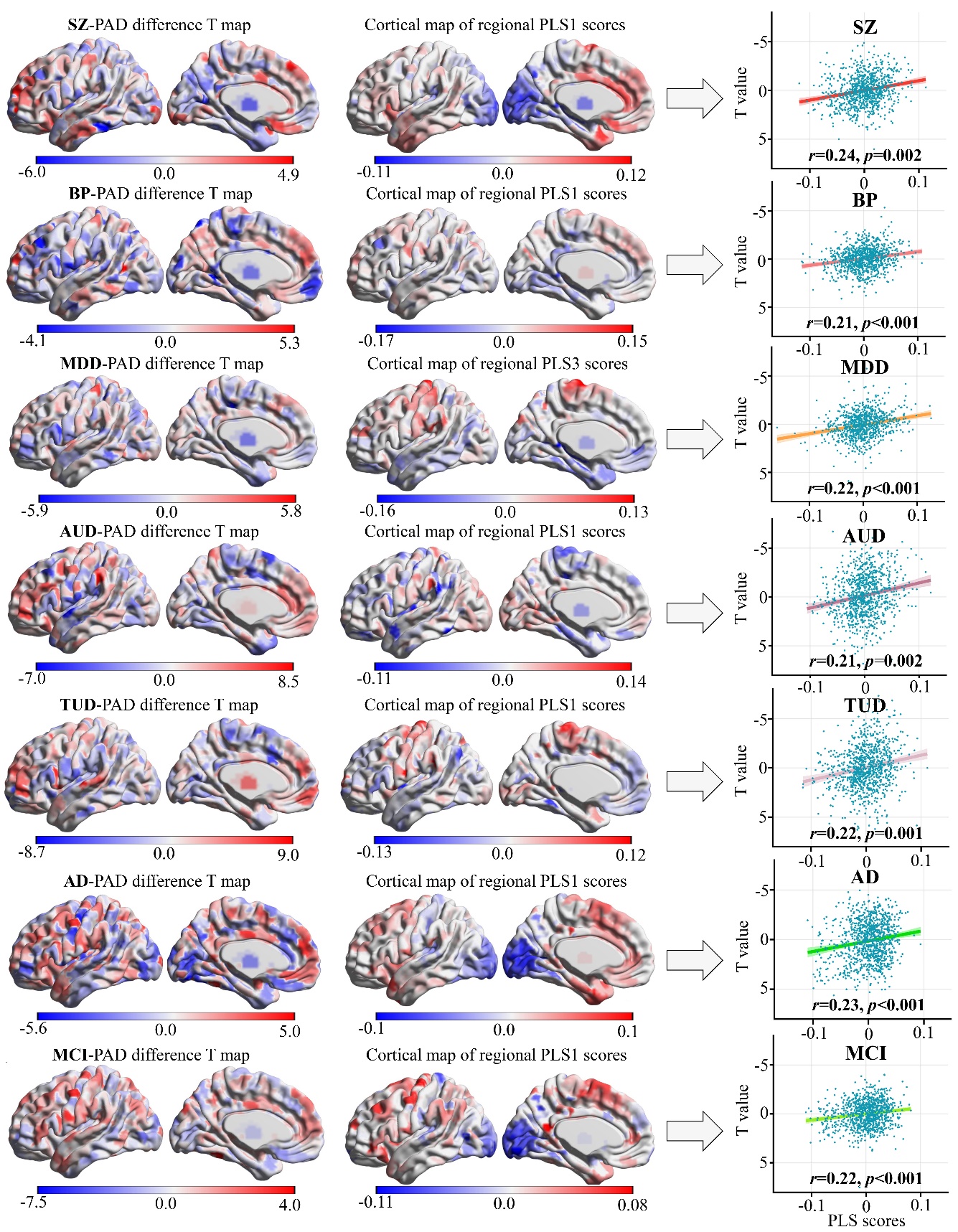


**Figure L. Expression profiles of genes related to PAD difference T-map in diagnostic group**. Cortical maps and correlation scatterplots of PAD difference T value and the corresponding regional PLS1 (PLS3 for MDD) scores. The *p* values were obtained from permutation test (1000 permutations) with corrected spatial autocorrelation. The solid line indicates the linear regression fit. SZ: schizophrenia; BP: bipolar disorder; MDD: major depressive disorder; AUD: alcohol use disorder; TUD: tobacco use disorder; AD: Alzheimer's disease; MCI: mild cognitive impairment; PLS: partial least square.


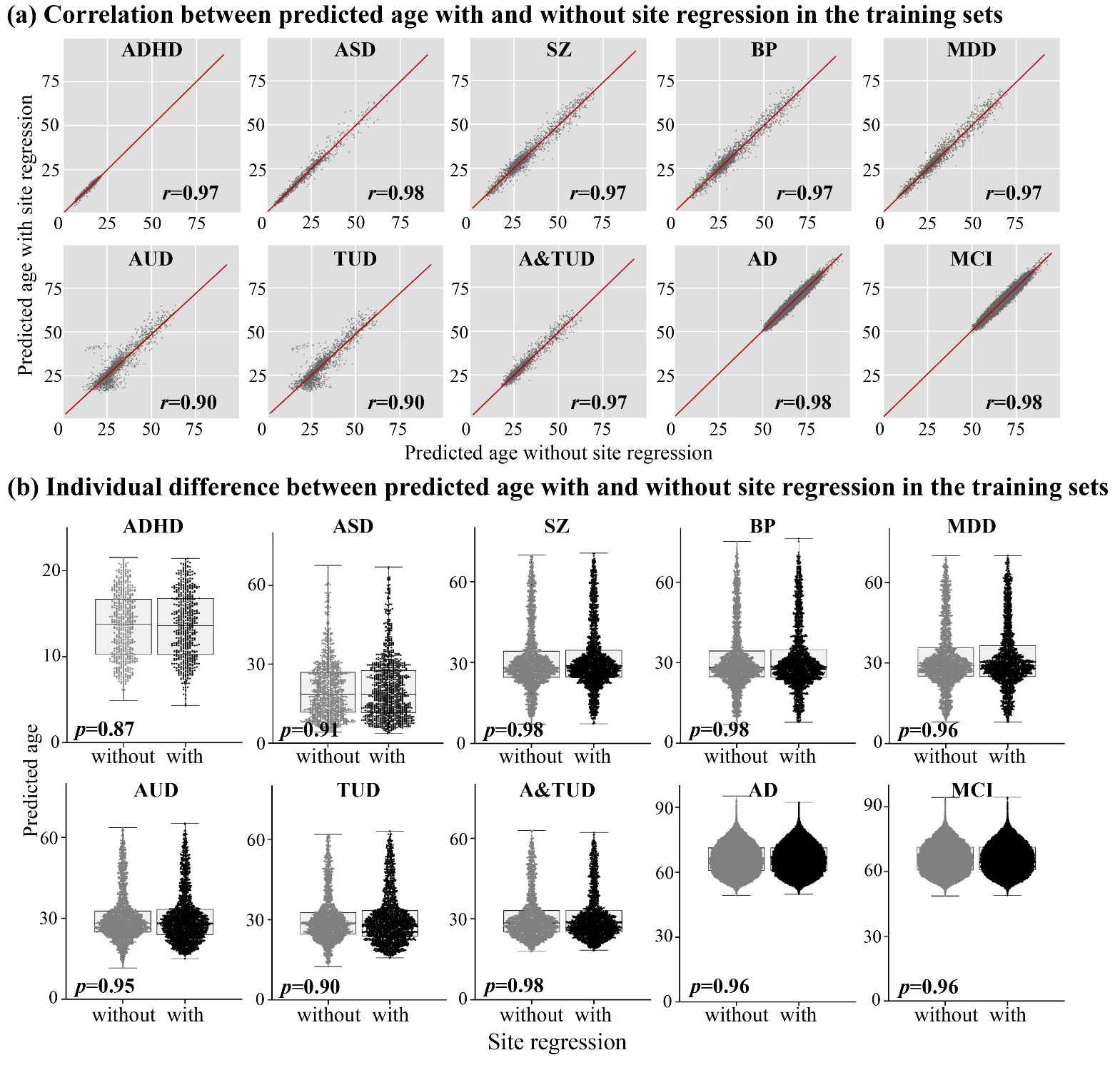


**Figure M. The (a) correlation and (b) individual difference between predicted age with and without site regression in the training sets.** The solid line indicates the linear regression fit. The *p* values were calculated using paired *t*-tests. In boxplots, the lower, middle, and upper bounds of the box represent the first quartile, median, and third quartile, respectively. The lower and upper whiskers represent the minimum and maximum values, respectively. ADHD: attention-deficit/hyperactivity disorder; ASD: autism spectrum disorder; SZ: schizophrenia; BP: bipolar disorder; MDD: major depressive disorder; AUD: alcohol use disorder; TUD: tobacco use disorder; A&TUD: AUD and TUD; AD: Alzheimer's disease; MCI: mild cognitive impairment.


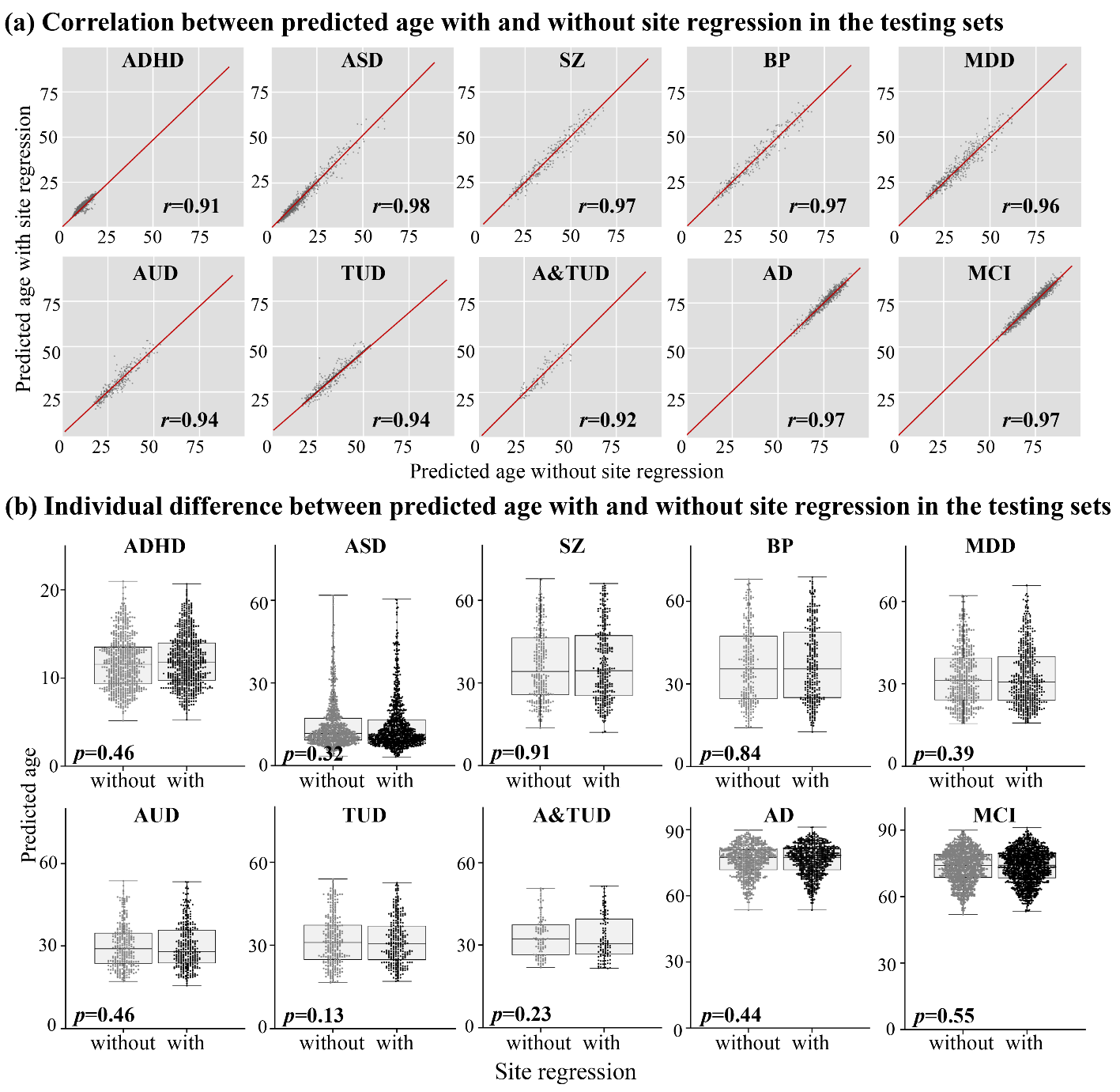


**Figure N. The (a) correlation and (b) individual difference between predicted age with and without site regression in the testing sets.** The solid line indicates the linear regression fit. The *p* values were calculated using paired *t*-tests. In boxplots, the lower, middle, and upper bounds of the box represent the first quartile, median, and third quartile, respectively. The lower and upper whiskers represent the minimum and maximum values, respectively. ADHD: attention-deficit/hyperactivity disorder; ASD: autism spectrum disorder; SZ: schizophrenia; BP: bipolar disorder; MDD: major depressive disorder; AUD: alcohol use disorder; TUD: tobacco use disorder; A&TUD: AUD and TUD; AD: Alzheimer's disease; MCI: mild cognitive impairment.


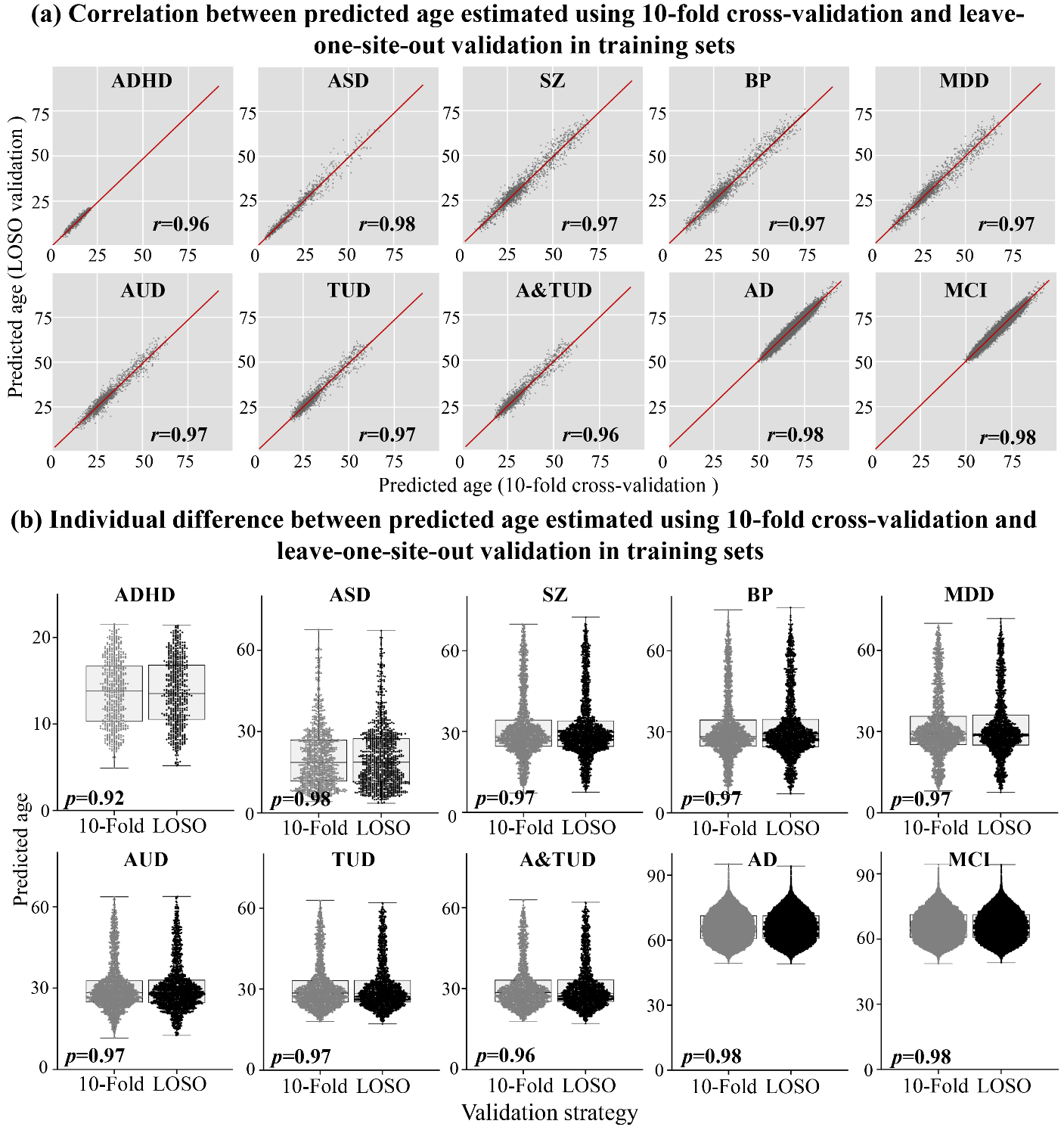


**Figure O. The (a) correlation and (b) individual difference between predicted age estimated using 10-fold cross-validation and leave-one-site-out validation in training sets.** The solid line indicates the linear regression fit. The *p* values were calculated using paired *t*-tests. In boxplots, the lower, middle, and upper bounds of the box represent the first quartile, median, and third quartile, respectively. The lower and upper whiskers represent the minimum and maximum values, respectively. ADHD: attention-deficit/hyperactivity disorder; ASD: autism spectrum disorder; SZ: schizophrenia; BP: bipolar disorder; MDD: major depressive disorder; AUD: alcohol use disorder; TUD: tobacco use disorder; A&TUD: AUD and TUD; AD: Alzheimer's disease; MCI: mild cognitive impairment; LOSO: leave-one-site-out.
